# Transcriptomic insight into terpenoid and carbazole alkaloid biosynthesis, and functional characterization of two terpene synthases in curry tree (*Murraya koenigii*)

**DOI:** 10.1038/srep44126

**Published:** 2017-03-08

**Authors:** Seema Meena, Sarma Rajeev Kumar, Varun Dwivedi, Anup Kumar Singh, Chandan S. Chanotiya, Md. Qussen Akhtar, Krishna Kumar, Ajit Kumar Shasany, Dinesh A. Nagegowda

**Affiliations:** 1 Molecular Plant Biology and Biotechnology Laboratory, CSIR-Central Institute of Medicinal and Aromatic Plants Research Centre, GKVK Post, Bengaluru - 560065, India; 2 Laboratory of Aromatic Plants and Chiral Separation, CSIR-Central Institute of Medicinal and Aromatic Plants, P.O. CIMAP, Lucknow - 226015, India; 3 Biotechnology Division, CSIR-Central Institute of Medicinal and Aromatic Plants, P.O. CIMAP, Lucknow - 226015, India

## Abstract

Curry tree (*Murraya koenigii* L.) is a rich source of aromatic terpenes and pharmacologically important carbazole alkaloids. Here, *M. koenigii* leaf transcriptome was generated to gain insight into terpenoid and alkaloid biosynthesis. Analysis of *de novo* assembled contigs yielded genes for terpene backbone biosynthesis and terpene synthases. Also, gene families possibly involved in carbazole alkaloid formation were identified that included polyketide synthases, prenyltransferases, methyltransferases and cytochrome P450s. Further, two genes encoding terpene synthases (MkTPS1 and MkTPS2) with highest *in silico* transcript abundance were cloned and functionally characterized to determine their involvement in leaf volatile formation. Subcellular localization using GFP fusions revealed the plastidial and cytosolic localization of MkTPS1 and MkTPS2, respectively. Enzymatic characterization demonstrated the monoterpene synthase activity of recombinant MkTPS1, which produced primarily (−)-sabinene from geranyl diphosphate (GPP). Recombinant MkTPS2 exhibited sesquiterpene synthase activity and formed (*E,E*)-α-farnesene as the major product from farnesyl diphosphate (FPP). Moreover, mRNA expression and leaf volatile analyses indicated that MkTPS1 accounts for (−)-sabinene emitted by *M. koenigii* leaves. Overall, the transcriptome data generated in this study will be a great resource and the start point for characterizing genes involved in the biosynthetic pathway of medicinally important carbazole alkaloids.

*Murraya koenigii* (L.) Spreng. popularly known as “Curry Tree” belongs to the genus *Murraya* of Rutaceae family and is cultivated for its aromatic leaves, which are widely used in Indian cuisine. The genus consists of about 11 species globally and is distributed from tropical to subtropical regions of Asia^1^. The essential oil of *M. koenigii* is highly valued and mainly used in flavor and fragrance, cosmetics, soap industry, and aromatherapy^2^. In addition to its aromatic importance, *M. koenigii* is a rich source of bioactive carbazole alkaloids like girinimbine, mahanimbine, murrayafoline, and pyrafoline-D that possess numerous pharmacological activities such as anticancer, antidiabetic, antimicrobial and antioxidant properties^3^ (Table S1). *M. koenigii* is also ethnopharmacologically important as different parts of the plant are used in traditional medicine to cure various ailments and for the treatment of asthma, prostate cancer and obesity^4^,^5^,^6^. Traditionally, extracts from different parts of *M. koenigii* plant are also used as analgesic, febrifuge, stomachic, carminative, and for the treatment of diarrhoea, dysentery, and skin eruptions.

In plants, terpenoids are derived from isopentenyl diphosphate (IDP) and dimethylallyl diphosphate (DMADP) which are biosynthesized via the plastidial MEP (2-*C*-methylerythritol-4-phosphate) pathway and cytosolic/peroxisomal MVA (mevalonate) pathway (Fig. 1)^7^,^8^. Later these starter molecules are converted to immediate prenyl diphosphate precursors such as geranyl diphosphate (GDP), farnesyl diphosphate (FDP), neryl diphosphate (NDP), and geranylgeranyl diphosphate (GGDP) by the action of respective prenyl diphosphate synthases. The prenyl diphosphates in respective compartments are then utilized by various terpene synthases (TPSs) resulting in highly diverse class of compounds that include mono-, sesqui-, di-, tri- and tetra- terpenes. Unlike terpenoids, the biosynthesis of carbazole alkaloids in higher plants is not well understood. They are proposed to be derived from shikimic acid that proceeds via chorismic acid and isochorismic acid to give anthranilic acid, which further gets converted to quinolone^3^ (Fig. 1). Prenylation of quinolone with prenyl pyrophosphate (PP) derived either from MVA or MEP pathway could form 3-prenylquinolone and 2-prenylindole, finally resulting in 3-methylcarbazole. At later stages of the biosynthetic pathway, 3-methylcarbazole may undergo different modification steps to yield array of carbazole alkaloids^3^,^9^.

Although *M. koenigii* is an important aromatic and medicinal plant, molecular studies with respect to the biosynthesis of different types of secondary metabolites have not been explored till date. In this work, leaf transcriptome sequencing and analysis was carried out as the first step towards understanding the biosynthesis of specialized terpenoids and carbazole alkaloids in *M. koenigii*. Putative genes involved in the formation of volatile terpenes and carbazole alkaloids were mined and analyzed. Moreover, genes encoding (−)-sabinene synthase and (*E,E*)-α-farnesene synthase were functionally characterized.

## Results

### Transcriptome sequencing, assembly and annotation

*M. koenigii* leaf cDNA library was prepared (Table S2) and sequenced by Illumina HiSeq2000 platform. Obtained sequences/raw reads were deposited at NCBI Short Read Archive (SRA) and Transcriptome Shotgun Assembly (TSA) databases under the accession numbers SRR2970920 (Bioproject PRJNA304945 and Biosample SAMN04311782) and GFCO01000000, respectively. *De novo* assembled contig lengths were distributed between <150 bp to >4000 bp (Fig. S1a). The average transcript length and mean GC% of assembled contigs were 1163.43 bp and 40.79%, respectively (Table S2 and Fig. S2a). Contigs having <150 bp length were excluded from downstream analysis. Analysis of FPKM (Fragments Per Kilobase of transcript per Million Mapped reads) showed that 102857 contigs, representing 90.28% of total contigs, were having ≥1 FPKM (Fig. S1b). Annotation of these contigs against public databases showed that 87417 (84.98%) contigs had at least one significant hit against NCBI database (Table S3 and Fig. S2b,c). The top BLASTX hit of each 87417 contigs against NCBI nr database revealed similarity with sequences of 650 organisms, of which only six were having occurrence frequency of >1000 transcripts. Among the six organisms, *Citrus clementia* exhibited the highest occurrence frequency of 47.8% followed by *Citrus sinensis* with 35.5% (Fig. S1c). In total, 35812 (40.97%) annotated contigs representing 2636 “GO” terms were distributed among three main ontologies comprising “biological processes” with maximum number of 1265 terms followed by “molecular function” and “cellular components” with 1035 and 336 terms, respectively (Fig. S3).

### Secondary metabolic pathways in curry leaf

**Figure 2:**
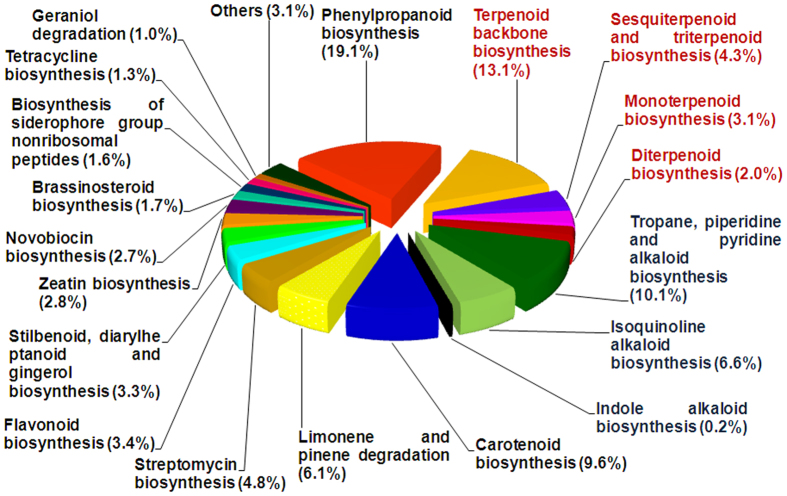
Distribution of contigs under secondary metabolism categories. Contig distribution under secondary metabolism categories based on KEGG classifications and the relative percentage of contigs involved in various secondary metabolic pathways. Numbers in brackets indicate relative % representation.

To gain insight into secondary metabolic pathways in *M. koenigii,* all assembled contigs with ≥150 bp and ≥1 FPKM were mapped against KEGG (Kyoto Encyclopedia of Genes and Genomes) database using KEGG Automatic Annotation Server (KAAS). A total of 25809 contigs were annotated and mapped to 325 canonical pathways including primary and secondary metabolic processes (Fig. S4). Maximum percentage of contigs (39.02%) were found to be involved in metabolism, of which 9.9% contigs were putatively involved in 30 secondary metabolic pathways (Fig. 2 and Fig. S4). Majority of the contigs under “secondary metabolic pathways” were annotated to be involved in terpenoid biosynthesis (22.5%), including terpenoid backbone, mono-, sesqui-, di-, and tri-terpenoid biosynthetic pathways (Fig. 2). Also, significant percentage of contigs belonged to phenylpropanoid (19.1%) and different alkaloid biosynthetic pathways (16.9%). Within terpenoid backbone biosynthesis, 60 contigs corresponding to 7 genes of MEP pathway and 43 contigs for 6 genes of MVA pathway were identified with a cumulative FPKM of 3413 and 509, respectively (Table 1). The number of annotated contigs for downstream genes were 11 (IDI), 15 (GDS), 41 (GGDS) and 6 (FDS) with a cumulative FPKM of 833, 292, 1906 and 74, respectively. Mono- and sesqui- terpene synthases were represented by 36 and 70 contigs having FPKM of 2828 and 1928, respectively (Table 1). Contigs related to anthranilate pathway leading to synthesis of quinolone included 112 contigs accounting for 5 genes with a combined FPKM of 4661 (Table 1).

### Phylogenetic analysis of polyketide synthases from *M. koenigii*

**Figure 3:**
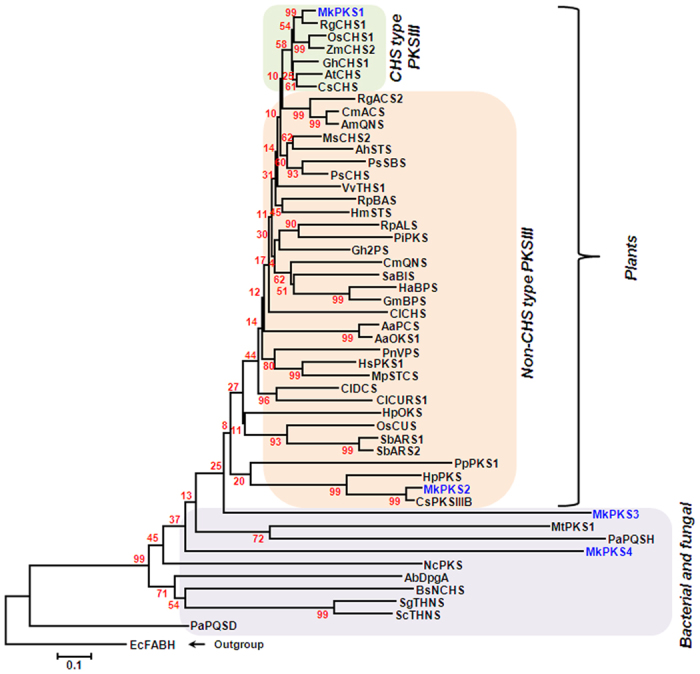
Phylogenetic relatedness of *M. koenigii* PKS candidates with other polyketide synthases. A bootstrap value of 1000 runs was used to calculate the distance using MEGA 6 software. AaOKS1, *Aloe arborescens* octaketide synthase 1; AaPCS, *A. arborescens* pentaketide chromone synthase; AbDpgA, *Amycolatopsis balhimycina* dihydroxyphenylacetic acid synthase; AhSTS, *Arachis hypogaea* stilbene synthase; AmQNS, *Aegle marmelos* quinolone synthase; AtCHS, *Arabidopsis thaliana* chalcone synthase; BsNCHS, *Bacillus subtilis* naringenin-chalcone synthase; CsCHS, *Cannabis sativa* chalcone synthase; CmACS, *Citrus microcarpa* acridone synthase; CmQNS, *C. microcarpa* quinolone synthase; CsPKSIIIB, *C. sinensis* polyketide synthase type III B; ClCHS, *Curcuma longa* chalcone synthase; ClDCS, *C. longa* diketide CoA synthase; ClCURS1, *C. longa* curcumin synthase 1; EcFABH, *Escherichia coli* K-12 beta-ketoacyl-ACP synthase III; Gh2PS, *Gerbera hybrida* 2-pyrone synthase; GhCHS1, *G. hybrida* naringenin-chalcone synthase 1; GmBPS, *Garcinia mangostana* benzophenone synthase; HaBPS, *Hypericum androsaemum* benzophenone synthase; HpOKS, *H. perforatum* octaketide synthase; HpPKS, *H. perforatum* polyketide synthase; HmSTS, *Hydrangea macrophylla* stilbenecarboxylate synthase; HsPKS1, *Huperzia serrata* chalcone synthase-like polyketide synthase 1; MpSTCS, *Marchantia polymorpha* stilbenecarboxylate synthase 2; MsCHS2, *Medicago sativa* chalcone synthase 2; MtPKS1, *Mycobacterium tuberculosis* phenolpthiocerol synthesis type-I polyketide synthase; NcPKS, *Neurospora crassa* type III pentaketide synthase; OsCHS1, *Oryza sativa* chalcone synthase 1; OsCUS, *O. sativa* curcuminoid synthase; PaPQSD, *Pseudomonas aeruginosa* 2-heptyl-4(1 H)-quinolone synthase; PaPQSH, *P. aeruginosa* 2-heptyl-3-hydroxy-4(1 H)-quinolone synthase; PBBS, *Phalaenopsis* sp. bibenzyl synthase; PiPKS, *Plumbago indica* polyketide synthase; PnVPS, *Psilotum nudum* valerophenone synthase; PpPKS1, *Physcomitrella patens* 2′-oxoalkylresorcinol synthase; PsCHS, *Pisum sativum* chalcone synthase; PsCHS, *Pinus strobus* chalcone synthase; PsSBS, *P. strobus* stilbene synthase; RgACS2, *Ruta graveolens* acridone synthase 2; RgCHS1, *R. graveolens* chalcone synthase 1; RpALS, *Rheum palmatum* aloesone synthase; RpBAS, *R. palmatum* benzalacetone synthase; SaBIS, *Sorbus aucuparia* biphenyl synthase; SbARS1, *Sorghum bicolor* alkylresorcinol synthase 1; SbARS2, *S. bicolor* alkylresorcinol synthase 2; ScTHNS, *Streptomyces coelicolor* 1,3,6,8-tetrahydroxynaphthalene synthase; SgTHNS, *Streptomyces griseus* 1,3,6,8-tetrahydroxynaphthalene synthase; VvTHS1, *Vitis vinifera* resveratrol synthase 1; ZmCHS2, *Zea mays* chalcone synthase 2. Accession numbers of protein sequences used in this figure are given in Supplementary Information.

The enzymes catalyzing quinolone formation belong to a group of multifunctional polyketide synthases (PKSs). PKSs characterized from plants belong to PKSIII superfamily, which is broadly classified into chalcone synthase-type (CHS-type) PKSIII and non-chalcone synthase type (non-CHS type) PKSIII (Fig. 3). Search of curry leaf transcriptome to identify candidates that could be involved in carbazole alkaloid biosynthesis resulted in 44 contigs annotated as PKSs, which were having a combined FPKM of 2169. Out of these contigs, 4 full length PKS candidates (MkPKS1-4) were identified and used to build phylogenetic relationship with other characterized PKSs. While MkPKS1 was grouped with the CHS-type PKSIII suggesting it to be a chalcone synthase (CHS), MkPKS2 was grouped together with non-CHS type PKSIII that are involved in the formation of different alkaloids (Fig. 3). MkPKS3 and MkPKS4 were grouped with multifunctional PKSs from bacteria and fungus that included MtPKS1 (*Mycobacterium tuberculosis* PKS) involved in the synthesis of the immunomodulatory phenolic glycolipids (PGLs)^10^ and PaPQSH (*Pseudomonas aeruginosa* 2-heptyl-3-hydroxy-4(1H)-quinolone synthase) synthesizing quinolone as a signaling molecule^11^ (Fig. 3). The catalytic triad Cys-His-Asn present within the active sites of all characterized type-III PKSs from plants was also found in MkPKS1 and MkPKS2 (Fig. S5). This catalytic triad catalyzes the repetitive condensations of C_2_ units derived from CoA-linked starter molecules^12^. Based on the phylogenetic analysis, the role of PKSs in synthesizing one or multiple steps of carbazole alkaloids may be hypothesized.

### Genes for downstream carbazole alkaloid formation

In addition to PKS candidates, transcriptome mining also yielded contigs for prenyltransferases (PTs), methyltransferases (MTs), and cytochrome P450 enzymes (CYP450s), of which some may be involved in the formation of carbazole alkaloids^3^,^9^. A total of 119 contigs were annotated as PTs in the transcriptome having different metabolic pathways assigned to them. However, 17 contigs with “prenyltransferase activity” were not assigned to any putative pathways indicating that they could be further studied for their involvement in prenylation of quinolone (Table S4). In the case of MTs, out of 172 annotated contigs, 37 were annotated as either “MT”, “MT family protein” or “putative/uncharacterized MTs” (Table S5), suggesting their possible role in alkaloid biosynthesis. *M. koenigii* transcriptome also comprised 452 CYP450 candidates that were distributed among 38 CYP450 families (Table S6). Among them, candidates belonging to various CYP450 gene families reported in the biosynthesis of terpenoids and alkaloids were also identified. They included CYP51, CYP71, CYP73, CYP76, CYP82, CYP88, CYP98, CYP706 and CYP716 families. Future functional studies on these candidates may shed light on their involvement in carbazole alkaloid biosynthesis.

### Phylogenetic analysis of *M. koenigii* terpene synthases

**Figure 4:**
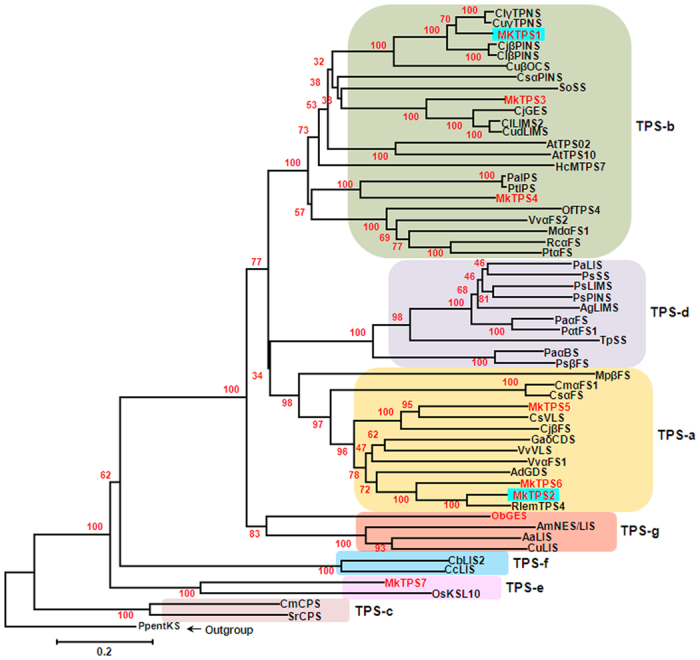
Phylogenetic relationship of *M. koenigii* TPS candidates with other terpene synthases. An unrooted neighbor joining tree of *M. koenigii* and other terpene synthases with bootstrap value of 1000 runs. The color shaded boxes represent TPS subfamilies a–g. The characterized TPSs in this study are boxed in blue. AbLIMS, *Abies grandis* (−)-4*S*-limonene synthase; AaLIS, *Actinidia arguta* linalool synthase; AdGDS, *Actinidia deliciosa* germacrene-D synthase; AmNES/LIS-1, *Antirrhinum majus* nerolidol/linalool synthase 1; AtTPS02, *Arabidopsis thaliana* terpene synthase 02; AtTPS10, *A. thaliana* terpene synthase10; CsαPINS, *Cannabis sativa* (+)-α-pinene synthase; CjGES, *Citrus jambhiri* geraniol synthase; RlemTPS2, *C. jambhiri* sabinene synthase; RlemTPS4, *C. jambhiri* δ-elemene synthase; CjβPINS, *C. jambhiri* β- pinene synthase; CjuβFS, *C. junos (E*)-β-farnesene synthase; ClLIMS2, *C. limon* limonene synthase 2; ClβPINS, *C. limon* (−)-β-pinene synthase; ClγTPNS, *C. limon* γ-terpinene synthase; CsiVLS, *C. sinensis* valencene synthase; CudLIMS, *C. unshiu* limonene synthase; CuLIS, *C. unshiu* linalool synthase; CuβOCS, *C. unshiu (E*)-β-ocimene synthase; CuγTPNS, *C. unshiu* γ-terpinene synthase; CbLIS2, *Clarkia breweri* linalool synthase 2; CcLIS, *C. concinna* linalool synthase; Cmα-FS1, *Cucumis melo* α-farnesene synthase; CsαFS, *C. sativus (E,E*)-α-farnesene synthase; CmCPS, *Cucurbita maxima* copalyl diphosphate sythase; GaδCDS, *Gossypium arboreum* (+)-δ-cadinene synthase; HcMTPS7, *Hedychium coronarium* chloroplast monoterpene synthase; MdαFS, *Malus domestica (E,E*)-α-farnesene synthase; MpβFS, *Mentha piperita* β-farnesene synthase; ObGES, *Ocimum basilicum* geraniol synthase; OsKSL10, *Oryza sativa Ent*-sandaracopimaradiene synthase; OfTPS4, *Osmanthus fragrans* α-farnesene synthase; Pp*ent*-KS, *Physcomitrella patens* (−)-*ent*-kaurene synthase; PaαBS, *Picea abies E*-α-bisabolene synthase; PaLIS, *P. abies* (−)-linalool synthase; PaαFS, *P. abies (E,E)*-α-farnesene synthase; PsLIMS, *P. sitchensis* (−)-limonene synthase; PsPINS, *P. sitchensis* pinene synthase; PsSS, *P. sitchensis* (+)-sabinene synthase; *Pinus sylvestris E*-β-farnesene synthase; PtαFS Psβ-FS, *Pinus taeda* α-farnesene synthase; Ptα-FS, *P. taeda* α-farnesene synthase; PaIPS, *Populus alba* isoprene synthase; PtIPS, *P. tremuloides* isoprene synthase; Rcα-FS, *Ricinus communis* α-farnesene synthase; SoSS, *Salvia officinalis* (+)-sabinene synthase; SrCPS, *Stevia rebaudiana* copalyl pyrophosphate synthase; TpSS, *Thuja plicata* sabinene synthase; Vvα-FS1, *Vitis vinifera* α-farnesene synthase; VvαFS2, *V. vinifera* α-farnesene/β-ocimene synthase; VvVLS, *V. vinifera* valencene synthase. Accession numbers of protein sequences used in this figure are given in Supplementary Information.

Search for TPS candidates using KEGG, NCBI and uniprot databases yielded 160 contigs annotated as putative TPSs (Table 1). Further search for full length TPS candidates yielded 7 full length sequences designated as MkTPS1 to 7 encoding putative mono-, sesqui- and di- terpene synthases. Phylogenetic analysis of these TPS proteins showed that MkTPS1 (602 aa), MkTPS3 (607 aa) and MkTPS4 (608 aa) were clustered with TPS-b subfamily, which consisted mainly monoterpene synthases, whereas MkTPS2 (560 aa), MkTPS5 (562 aa) and MkTPS6 (562 aa) grouped with TPS-a subfamily representing sesquiterpene synthases (Fig. 4). MkTPS7 (781 aa) formed a separate clade with TPS-e subfamily consisting of diterpene synthases. While MkTPS1 and MkTPS3 exhibited highest identities to *C. limon* γ-terpinene synthase (Clγ-TPNS) (86% identity) and limonene synthase (ClLIMS) (71% identity), respectively, MkTPS4 was closest to *Populus alba* isoprene synthase (PaIPS) (54% identity) (Fig. 4). Within TPS-a subfamily, MkTPS2 showed highest identity (83%) to δ-elemene synthase from *C. jambhiri* (RlemTPS4). MkTPS5 and MkTPS6 exhibited 63 and 88% identities to *C. sinensis* valencene synthase (CsVLS) and *C. unshiu* linalool synthase (CuLIS), respectively, whereas MkTPS7 exhibited 65% identity to *Malus domestica* kaurene synthase (MdKS), a diterpene synthase. The arginine-tryptophan rich motif “R(R)X_8_W” was found at the N-terminus of all TPSs except MkTPS7 of TPS-e subfamily that lacks this motif or may have a highly modified version of it^13^ (Fig. S6). This motif may play a role in the initiation of isomerization-cyclization reaction or stabilizes the protein through electrostatic interaction^13^. All seven TPS proteins contained highly conserved arginine-rich motif “RXR” that is believed to assist in directing the diphosphate anion away from the reactive carbocation after ionization^14^. Also, aspartate-rich metal ion binding motifs “DDXXD” and “(N/D)DXX(S/T)XXXE” were present at the C-terminus of all MkTPS protein sequences (Fig. S6).

### Subcellular localization of MkTPS1 and MkTPS2

**Figure 5:**
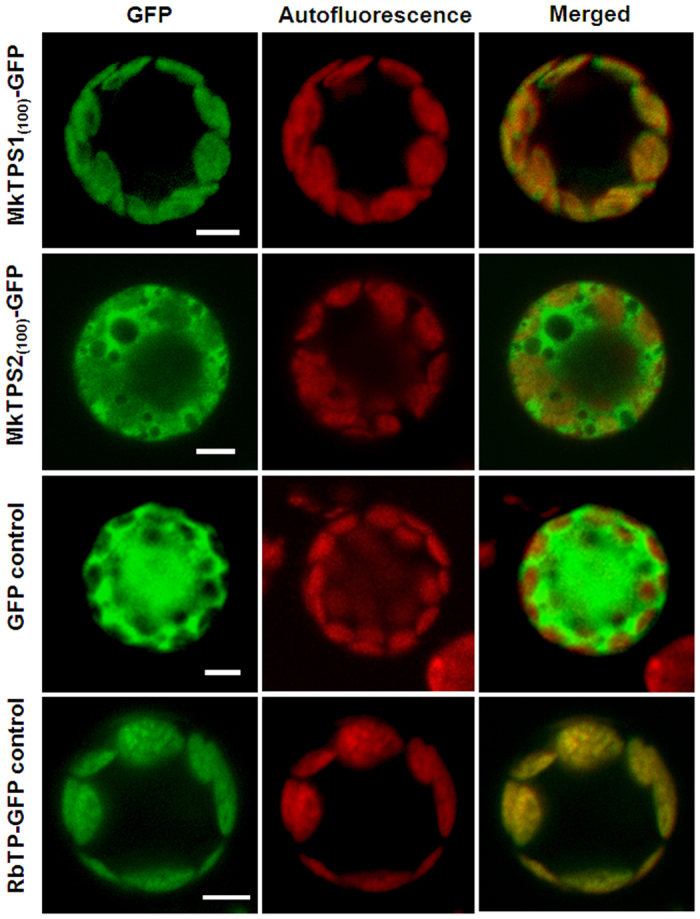
Subcellular localization of *M. koenigii* terpene synthases. Confocal laser scanning microscopy of MkTPS1 and MkTPS2 using GFP-fusion proteins in *Arabidopsis* protoplasts. Name of GFP fusion constructs are shown on the left, and the corresponding transient expression in the protoplasts is shown on the right. GFP fluorescence detected is shown in the ‘GFP’ column; chlorophyll autofluorescence is shown in the “Autofluorescence” column, and the “Merged” column shows combined GFP fluorescence and chlorophyll autofluorescence. Scale bars = 50 μm. The numbers in the fusion constructs correspond to amino acid positions. RbTP (plastidial RuBisCO target peptide)-GFP is a chloroplast marker.

To understand volatile terpenoid biosynthesis in curry leaf, two candidates, MkTPS1 (GenBank accession no. KX171229) and MkTPS2 (GenBank accession no. KX171230) having highest FPKM of 127.27 and 45.83, respectively, were characterized. *In silico* prediction analysis indicated that MkTPS1 and MkTPS2 to be plastidial and cytosolic, respectively, suggesting their putative mono- and sesqui- TPS activities (Table S7). To confirm their subcellular localization, the open reading frames (ORFs) of *MkTPS1* and *MkTPS2* were fused upstream of, and in frame with, GFP and the fusion constructs were transformed into *Arabidopsis* protoplasts. While the green fluorescence of MkTPS1_(100)_-GFP fusion protein was localized in the plastids similar to the RbTP-GFP that served as positive control for plastid localization, MkTPS2_(100)_-GFP exhibited cytosolic localization similar to the GFP control (Fig. 5). This was in agreement with other known monoterpene and sesquiterpene synthases and indicated that MkTPS1 and MkTPS2 could be involved in the formation of mono- and sesqui- terpenes emitted from *M. koenigii* leaves.

### Functional characterization of MkTPS1 and MkTPS2

**Figure 6:**
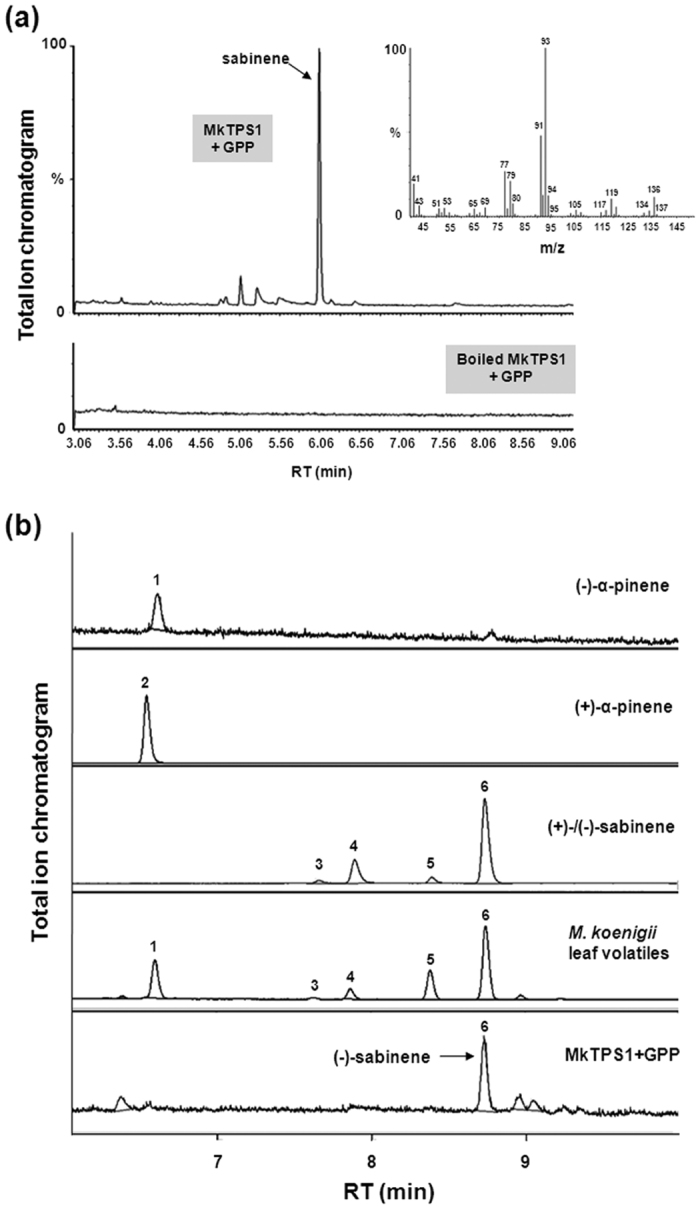
GC-MS analysis of products formed by recombinant MkTPS1 from geranyl diphosphate (GPP). (**a**) GC-MS chromatogram with mass spectra for the product formed by MkTPS1 enzyme from geranyl diphosphate (GPP). Terpene synthase assay was carried out using the Ni-NTA purified MkTPS1 in the presence of 70 μM GPP as substrate. Boiled protein was used as control. Reaction products were obtained by solid-phase microextraction (SPME) and analyzed by GC-MS. (**b**) Choromatograms of chiral GC analysis using authentic standards, leaf essential oil, and MkTPS1 assay product. The numbers labeled in the chromatogram are as follows: 1, (−)-α-pinene; 2, (+)-α-pinene; 3, (+)-β-pinene; 4, (−)-β-pinene; 5, (+)-sabinene; 6, (−)-sabinene.

**Figure 7:**
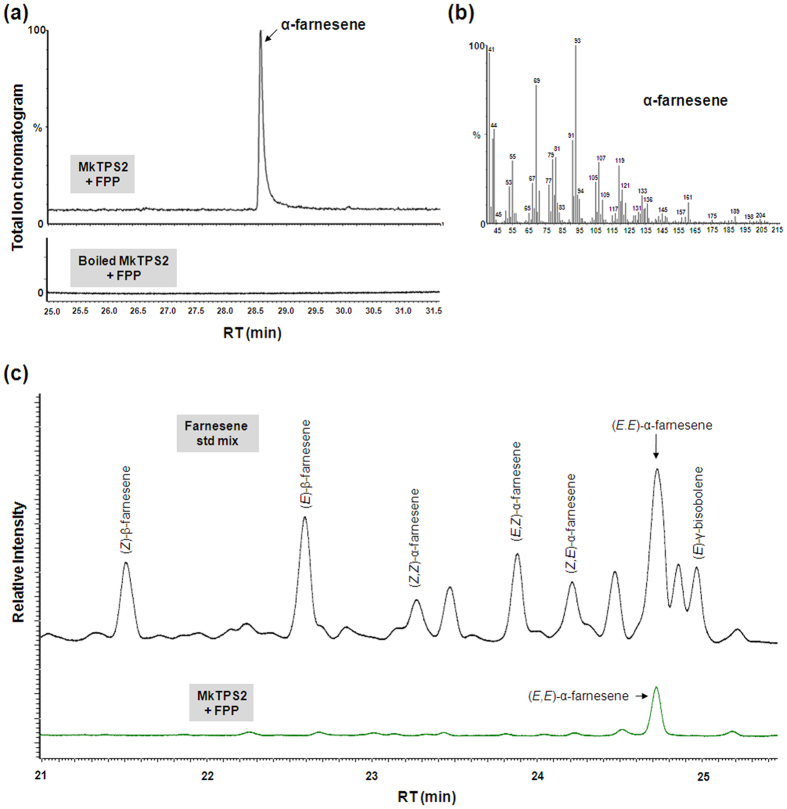
Analysis of reaction products formed by recombinant MkTPS2 from farnesyl diphosphate (FPP). (**a**) GC-MS chromatogram showing the product formed by MkTPS2 enzyme from FPP. (**b**) Mass spectra of α-farnesene. Terpene synthase assay was carried out using the purified recombinant MkTPS2 in the presence of 70 μM FPP as substrate. Boiled protein was used as control. Reaction products were obtained by solid-phase microextraction (SPME) and analyzed by GC-MS. (**c**) GC chromatograms of authentic farnesene standard mix (black) and MkTPS2 enzymatic product (green).

The coding regions of *MkTPS1* and *MkTPS2* were sub-cloned into pET32a expression vector, the encoded proteins were expressed in *Escherichia coli*, and purified recombinant proteins were analyzed for TPS activities. For *MkTPS1*cloning, the transit peptide was truncated at second methionine (Met-40) to avoid the formation of inclusion bodies, which is a typical feature of monoterpene synthases^15^,^16^. GC-MS analysis of the reaction products of MkTPS1 showed the formation of predominantly sabinene utilizing GPP as substrate whereas there was no product formed in the case of boiled enzyme (Fig. 6). Further, chiral analyses of the reaction products showed that MkTPS1 catalyzed the formation of (−)-sabinene. Chiral GC analysis of leaf essential oil also confirmed the presence of (−)-sabinene as a major leaf volatile along with (−)-α-pinene and (+)-sabinene (Fig. 6). Sabinene synthases (SSs) have been reported from five plant species including *Salvia officinalis* (SoSS)^17^, *C. jambhiri* (RlemTPS2)^18^, *Picea sitchensis* (PsSS)^19^, *Thuja plicata* (TpSS)^20^ and *Hedychium coronarium* (HcTPS7)^21^. Among the characterized SSs, MkTPS1 exhibited highest homology (80%) to RlemTPS2 (Table S8). Amino acid alignment revealed that catalytic active site residue Asn-348 is present at the 7^th^ position upstream of metal ion binding “DDXXD” motif in MkTPS1 (Fig. S6). In the case of MkTPS2, the product analysis showed the formation of α-farnesene with FPP as substrate. There was no product formation either in the boiled protein control (Fig. 7) or with GPP as substrate, indicating that MkTPS2 is a sesquiterpene synthase with *bona fide* α-farnesene synthase activity (Fig. 7a). Next, the geometric isomeric nature of the identified α-farnesene was determined to be (*E,E*)-α-farnesene by comparison with authentic farnesene mix (Fig. 7b and Fig. S10). Among the characterized plant α-farnesene synthases, MkTPS2 exhibited highest identity (54%) with *Vitis vinifera (E,E*)-α-farnesene synthase (VvαFS1)^13^ that catalyzed the formation of α-farnesene from FPP. Amino acid sequence alignment of MkTPS2 with characterized plant α-farnesene synthases showed the presence of all conserved characteristic motifs of terpene synthases (Fig. S8).

### Involvement of MkTPS1 and MkTPS2 in the formation of curry leaf volatiles

**Figure 8:**
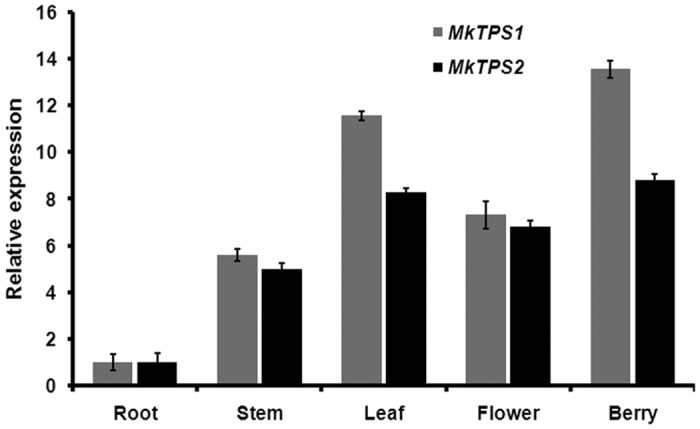
Tissue specific expression of *MkTPS1* and *MkTPS2*. Real-time qPCR expression analysis of *MkTPS1* and *MkTPS2* in different tissues of curry tree. *Actin* was used as the endogenous control. The expression of root was set to 1 to determine the relative abundance in other tissues.

In order to determine the involvement of characterized MkTPSs in curry leaf volatiles formation, tissue specific gene expression and headspace volatile compounds of *M. koenigii* leaves were analyzed. Before proceeding for quantitative Real Time PCR (qPCR) analysis to determine the expression of *MkTPS1* and *MkTPS2*, two endogenous reference genes *actin* and *F-BOX* were selected and validated for transcript normalization. Among them, *actin* exhibited highest stability in different tissues (Fig. S9). Root was used as calibrator to determine the relative expression in other tissues. Results showed the highest expression of both *MkTPS1* and *MkTPS2* in green berry and mature leaf followed by flower and stem (Fig. 8). While *MkTPS1* exhibited ~13.5 and ~11.5 fold expression in berry and leaf, the expression of *MkTPS2* was also higher in above tissues (~9 fold and 8 fold respectively). Among the two candidates, *MkTPS1* exhibited higher transcript abundance compared to *MkTPS2* in mature leaf and green berry (Fig. 8). Highest expression of both *MkTPS1* and *MkTPS2* in leaf and berry (which are the parts of the plant known for their higher aroma levels), indicated their involvement in the formation of major terpene volatiles. Subsequent GC-MS analysis of *M. koenigii* leaf volatiles revealed that, the emitted volatiles were dominated by monoterpenes with sabinene as the major compound (Fig. S11). The highest mRNA expression of MkTPS1 that catalyzes the formation of (−)-sabinene and the presence of (−)-sabinene as the major volatile compound indicated the involvement of MkTPS1 in (−)-sabinene formation in curry tree leaf. However, (*E,E*)-α-farnesene formed by MkTPS2 was not detected in the headspace of curry leaf (Fig. S11).

## Discussion

In an attempt to understand the molecular basis of aroma and carbazole alkaloid biosynthesis, a RNA-Seq technology was employed to sequence the leaf transcriptome of curry tree. The high similarity (83%) of *M. koenigii* annotated contigs to *Citrus* species indicated the closeness of both species belonging to Rutaceae family (Fig. S1c). High representation and abundance of contigs related to terpenoid and alkaloid biosynthetic pathways in the leaf transcriptome supported high amounts of terpenoid volatiles and alkaloids in *M. koenigii* leaf (Table 1). In addition, presence of significant number of contigs for mono- and sesqui- TPSs with higher abundance (FPKM) indicated their involvement in formation of *M. koenigii* leaf volatiles.

Genes encoding PKS have been characterized from plant, fungal and bacterial species and are shown to have a role in the biosynthesis of wide range of alkaloids (acridone, quinolone, curcuminoids), flavanoids, alkylresorcinols and antibiotics etc.^22^,^23^,^24^,^25^,^26^,^27^. In plants, PKSIII enzymes catalyzing quinolone (proposed intermediate for carbazole alkaloids) formation have been characterized in three species that include *Aegle marmelos* (QNS-quinolone synthase)^25^, *Citrus microcarpa*^26^ (QNS-quinolone synthase and ACS-acridone synthase) and *Rheum palmatum*^27^ (BAS- benzalacetone synthase). In *M. koenigii* transcriptome, three potential PKSs, MkPKS2, MkPKS3 and MkPKS4 were found that could be putatively involved in carbazole alkaloid biosynthesis. While, MkPKS2 was grouped with multifunctional non-CHS type PKSIII proteins involved in plant alkaloid biosynthesis, MkPKS3 and MkPKS4 were grouped with bacterial and fungal PKSs that are involved in quinolone formation (Fig. 3). Future enzymatic and *in planta* characterization of these three candidates could reveal their role in carbazole alkaloid biosynthesis. In addition to PKSs, contigs were identified that encode putative PTs, MTs and CYP450s, which could be involved in downstream steps leading to array of carabazole alkaloids^3^,^9^ (Tables S4–S6).

In plants, generally monoterpene synthases are localized in plastids and sesquiterpene synthases are cytosolic^8^,^28^,^29^. MkTPS1 and MkTPS2 were grouped in TPS-b and TPS-a subfamily of mono- and sesqui- terpene synthases, respectively (Fig. 4). Also, GFP localization studies confirmed MkTPS1 and MkTPS2 to be plastidial and cytosolic proteins, respectively (Fig. 5). Functional characterization showed that the recombinant MkTPS1 catalyzed the formation of the predominantly monoterpene (−)-sabinene utilizing GPP as substrate (Fig. 6). Among the six sabinene synthases characterized so far, (−)-sabinene synthase has been reported only in Sitka spruce (*Picea sitchensis*) that catalyzed the formation of (−)-sabinene as the major product with trace amount of (+)-3-carene^30^. It was reported that amino acid in position 596 determined the product specificity in Sitka spruce sabinene synthase, where “Leu” and “Phe” specified the formation of (+)-3-carene and (−)-sabinene, respectively^30^. However, this was not the case with MkTPS1 that contained “Ala” similar to sabinene synthases of *S. officinalis* (SoSS) and *H. coronarium* (HcMTPS7)^21^ (Fig. S7). In addition, the presence of “Ile” at 7^th^ position upstream of “DDXXD” motif has been reported for the product specificity of sabinene synthases^18^,^31^. While, RlemTPS2 (sabinene synthase) exhibited pinene synthase activity when “Ile” was mutated to “Asn”^18^, *Salvia fruticosa* cineole synthase showed sabinene synthase activity when “Asn” was mutated to “Ile”^31^. In contrast, MkTPS1 has “Asn” instead of “Ile” at the corresponding position and still catalyzed formation of sabinene as the major product similar to HcTPS7^21^ (Fig. S7). Similarly, “Cys” instead of “Ileu” is present at the corresponding position in sabinene synthases of *P. sitchensis*^19^ and *T. plicata*^20^. These observations suggest that the product specificity of sabinene synthases may be determined by other amino acids in addition to “Phe” at position 596 as in the case of PsSS, and “Asn” or “Ileu” at 7^th^ position upstream of “DDXXD” motif. In the case of MkTPS2, product determination confirmed its sesquiterpene synthase activity forming (*E,E*)-α-farnesene as the major product with Mg^++^ as cofactor (Fig. 7). Among the characterized plant α-farnesene synthases, MkTPS2 exhibited highest amino acid identity (54%) with *Vitis vinifera* α-farnesene synthase (VvαFS1)^13^ and least identify (35%) with apple α-farnesene synthase (MdαFS1)^32^, both of which catalyzed formation of (*E,E*)-α-farnesene as the major product from FPP (Fig. S8 and Table S8). While both MkTPS2 and VvαFS1 belonged to TPS-a subfamily that also consisted of α-FSs from *C. melo* and *C. sativus*, other characterized plant α-FSs were distributed under TPS-b and TPS-d subfamilies (Fig. 4).

Transcript abundance or mRNA expression of corresponding genes has been reported to be positively correlated with accumulation and emission of volatile terpenes^33^,^34^,^35^,^36^,^37^. NGS studies have employed *in silico* gene expression methods such as Reads Per Kilobase of transcript per Million mapped reads (RPKM) and FPKM combined with aroma profiling to successfully identify and characterize genes involved in volatile terpene formation^34^,^36^. Both *MKTPS1* and *MkTPS2* were highly expressed in green berry and leaves with *MkTPS1* exhibiting higher expression than *MkTPS2*. High expression of *MkTPS1* and *MkTPS2* positively correlated with their high FPKM values (Fig. 8). Leaf essential oil analysis showed the presence of (−)-sabinene, (+)-sabinene, (−)-α-pinene, (−)-β-pinene and (+)-β-pinene with (−)-sabinene, (−)-α-pinene and (+)-sabinene as major terpenoid compounds in that order. Moreover, high expression of *MkTPS1* in leaf tissue implicated the involvement of MkTPS1 in production of (−)-sabinene, which is the major monoterpene among curry leaf volatiles (Fig. 6 and Fig. S11). Formation of (−)-sabinene as the sole product by MkTPS1 suggests that there could be isoforms of this gene that may be responsible for (+)-sabinene formation. Although a comparable level of *MkTPS2* expression to that of *MkTPS1* was found in all analyzed tissues, (*E,E*)-α-farnesene was not detected in volatile analysis. This lack of correlation between *MkTPS2* expression and (*E,E*)-α-farnesene emission in *M. koenigii* leaf (Fig. S11) could be due to the fact that sesquiterpenes could undergo complex processes of storage and conversion. A similar discrepancy between gene expression and volatile terpene emission has been reported in *Osmanthus fragrans*^34^*, Actinidia chinensis*^38^ and *Actinidia arguta*^39^. In conclusion, this is the first report of a transcriptome analysis and characterization of terpene synthases from economically and pharmacologically important genus “*Murraya*” and also from the tribe “clauseneae”. MkTPS1 characterized in this study could be employed for specific production of (−)-sabinene in heterologous systems. Further, the transcriptome data presented here will take the research on *M. koenigii* to the next level, facilitating characterization of genes involved in the biosynthesis of pharmacologically important carbazole alkaloids.

## Methods

### Plant material, library preparation and sequencing

Pooled leaf tissues were collected from a *M. koenigii* plant grown at CIMAP Research Centre, Bangalore, India. Total RNA was isolated from the leaf tissue following manufacturer’s protocol (Qiagen RNeasy mini kit cat # 74104). The RNA integrity was assessed using Qubit 2.0 Fluorometer with Qubit RNA BR Assay kit (Life Technologies, USA) and on a 2100 Bioanalyzer using an Agilent RNA 6000 Pico kit (Agilent Technologies, USA). Four μg of total RNA with an RNA Integrity Number (RIN) value of 7.0 was used for cDNA synthesis. cDNA library was prepared according to Illumina TruSeq RNA library protocol included in “TruSeq RNA Sample Preparation Guide” (Part # 15008136) provided with Illumina^®^ TruSeq™ RNA sample preparation kit. The library quality was assessed by 2100 Bioanalyzer using DNA 1000 kit (Agilent Technologies, USA), concentration was measured using Library quantification kit (Kapa Biosystems, USA), and sequencing was performed using HiSeq2000 platform (Illumina Inc., USA) after indexing the sample.

### Assembly, expression analysis and annotation

Raw reads obtained after sequencing were filtered by removing Illumina adapter using “cut-adapt”^40^ tool and in house “Perl scripts” was used to remove low quality bases (Q < 20) and to obtain processed reads. The trimmed reads were then *de novo* assembled using Velvet (Version 1.2.09) & Oases (Version 0.2.8) with kmer size of 41^41^,^42^. To calculate FPKM values, trimmed reads were first aligned to the assembled transcriptome using Bowtie2 program. Up to 1-mismatch was allowed in the seed region (length = 31 bp). Assembled contigs with >150 bp and expression value of >1 FPKM were annotated using BLASTX programme against various public databases including NCBI non-redundant (nr) protein database with set parameters of *E*-value >10^−5^ and similarity score of >40%, UniProt database for assigning Gene Ontology (GO) terms^43^, and KEGG using KEGG Automatic Annotation Server (KAAS; http://www.genome.jp/kaas-bin/kaas_main?mode=partial)^44^ for pathway assignment.

### Sequence analysis

Candidate transcripts were identified on the basis of their KEGG, NCBI and UniProt annotation. The transcripts were translated using ExPASy translate tool (http://web.expasy.org/translate) and sequence alignment was generated using MAFFT version 7 (http://mafft.cbrc,jp/alignmnet/software/) and BOXSHADE 3.21 (http://www.ch.embnet.org/software/BOX_form.html). Sequence and phylogenetic relatedness was analyzed using Molecular Evolutionary Genetics Analysis tool version 6 (MEGA 6) (http://www.megasoftware.net).

### Quantitative RT-PCR analysis

Total RNA was extracted from 100 mg of different tissues (root, stem, mature and leaf, flower, and berry) using Spectrum^TM^ Plant Total RNA Kit (Sigma–Aldrich, USA) following manufacturer’s instructions. On-column DNase digestion was performed to remove genomic DNA using DNase I (Sigma–Aldrich, USA) and cDNA was synthesized as reported^45^. Two μg of total RNA was used for first strand cDNA synthesis with random hexamer primers using High Capacity cDNA Reverse Transcription Kit (Applied Biosystems, USA). qPCR was performed as previously reported^46^. qPCR was performed with a linear range of cDNA using Step One Real Time PCR System (Applied Biosystems, USA). To validate the stability of reference genes to be used for qPCR normalization, *actin* and *F-BOX* (that has been previously used as reference gene for normalization in *Citrus* sp.) were used (Table S9). Among the two, only *actin* exhibited invariant expression in different tissues and hence was further used for normalization (Fig. S9). q-PCR was performed with 5 μl of total reaction with 2.5 μl of 2X Maxima SYBR Green PCR master mix (Thermo Scientific, USA), 1:10 diluted cDNA from different tissues and 2 μM gene-specific primers with following conditions, 94 °C for10 min for first cycle, that was followed by 40 cycles of 94 °C for15 s and 60 °C for 15 s. Fold change differences in gene expression were analyzed using the comparative cycle threshold (*Ct*) method using root cDNA as calibrator. All experiments were repeated using three technical replicates and data were analyzed statistically ( ± SD).

### Subcellular localization

The 300 bp ORF corresponding to first 100 amino acid was fused upstream of, and in frame with GFP between *Xba*I and *Bam*HI restriction sites of p326-sGFP vector containing the CaMV 35 S promoter (gift from Dr. I. Hwang, Pohang University of Science and Technology, Korea) (Table S9). Primers used for cloning into GFP vector are listed in Table S9. The fusion constructs were transformed into *Arabidopsis* protoplasts as described previously^47^. Transient expression of GFP fusion proteins was observed 16–20 h after transformation using confocal laser scanning microscope (Zeiss LSM 880) with a 40 × 1.55 numerical aperture lens. Fluorescence was collected with a bandpass filter and GFP excitation was measured at 488 nm with an argon laser and the fluorescence was detected at 520 nm. GFP and RbTP-GFP served as positive controls for cytoplasm and plastid localization, respectively.

### Expression and purification of recombinant MkTPS proteins

The ORFs of *MkTPS* candidates were amplified with forward and reverse primers consisting of start and stop codons, respectively (Table S9), along with restriction sites for cloning into pET28a vector. PCR was performed using Platinum Taq DNA polymerase High Fidelity (Invitrogen) and amplified PCR products were gel-purified using gel extraction kit (Fermentas, USA). PCR products were cloned into pJET 1.2/blunt cloning vector (CloneJET PCR cloning kit, Thermo Scientific) and transformed into *E. coli* XL1 for plasmid amplification. After restriction digestion and gel extraction, resulting fragments were sub-cloned into the expression vector pET32a (Novagen) downstream and in-frame of (His)_6_-tag. Positive clones were selected and resulting constructs were confirmed by restriction digestion and nucleotide sequencing. For functional expression, *E. coli* Rosetta-2 cells were transformed with recombinant pET32a containing MkTPS candidates. Induction, harvesting, and protein purification were performed as previously reported^46^. Briefly, a single colony was used to inoculate 25 ml Luria-Bertani broth with 37 mg ml^−1^ chloramphenicol and 50 mg ml^−1^ ampicillin and were grown overnight at 37 °C. 5 ml of these cultures were transferred to 1000 ml of the same medium and allowed to grow at 37 °C until an absorbance of 0.5 at OD_600_ nm. Cultures were then induced by adding isopropyl-1-thio-β-D-galactopyranoside (IPTG) to a final concentration of 0.4 mM and grown for an additional 18 h at 18 °C. Bacterial pellets having overexpressed recombinant proteins were used for protein purification by affinity chromatography on nickel-nitrilotriacetic (Ni-NTA) acid agarose (Bio-Rad, www.bio-rad.com). Protein concentration was determined using the Bradford method^48^.

### Terpene synthase assay and product identification

Terpene synthase assay was performed in 1000 μl assay buffer (30 mM HEPES, pH 7.5, 5 mM DTT, and 1 mM MgCl_2_) containing recombinant protein (5 μg) and 70 μM GPP/FPP. The contents were mixed in a 5 ml glass vial and connected to solid-phase microextraction (SPME) system to trap volatile products. After incubation for 2 h at 30 °C and 15 min at 42 °C, SPME system was removed from the tube and the adsorbed volatiles were analyzed by Gas Chromatography-Mass Spectrometry (GC-MS). Similarly, *M. koenigii* leaf volatiles were trapped by SPME system and analyzed by GC-MS.

#### Analysis of sabinene and farnesene

The enzymatic products were analyzed by GC-MS using a PerkinElmer Auto System XL GC interfaced with a TurboMass Quadrupole mass spectrometer. The column oven was programmed from 70 °C to 250 °C at a rate of 3 °C/min, with initial and final hold times of 2 min, and then programmed to 290 °C at 6 °C/min, with a final hold time of 5 min, using H_2_ as carrier gas at a constant pressure of 10 psi, a split ratio of 1:35, and injector and detector (FID) temperatures of 290 °C and 280 °C, respectively. Injector, transfer line and source temperatures were 250 °C, ionization energy 70 eV, and mass scan range 40–450 amu. Identification of compounds was achieved on the basis of retention time, elution order, relative retention index using a homologous series of *n*-alkanes (C_6_-C_28_ hydrocarbons, Polyscience Corp. Niles IL), mass spectral library search (NIST/EPA/NIH version 2.1 and Wiley registry of mass spectral data 7^th^ edition) and by comparing with the mass spectral literature data.

### Analysis of chirality of sabinene and geometric isomer of farnesene

#### Chiral analysis

Fresh leaf tissue (200 g) of *M. koenigii* was used for hydro-distillation using clevenger apparatus for 3–4 h at 50 °C to extract leaf essential oil, which was dried over anhydrous Na_2_SO_4_. To determine the chirality of sabinene in the assay product and leaf essential oil, volatiles were separated by GC using chiral column as reported previously^49^. The resolved enantiomers of sabinene were identified based on their retention time, elution order, co-injection of authentic standard (Aldrich, Cat No. W530597-SAMPLE-K) and relative retention index using a homologous series of n-alkanes (C_6_-C_28_ hydrocarbons, Polyscience Corp. Niles IL).

#### Geometric isomer analysis

The geometric isomeric nature of α-farnesene was determined by GC-MS analysis using farnesene standard mixture (Sigma Aldrich, Cat No. W383902-SAMPLE-K) in a GC7890B (Agilent Technologies) equipped with MS detector 5977A MSD (Agilent Technologies). To confirm the geometric isomer of product formed by MkTPS2, the assay product and farnesene standard mixture were analyzed by GC 7890B (Agilent Technologies) under same conditions that was used in GC-MS for farnesene.

## Additional Information

**How to cite this article**: Meena, S. *et al*. Transcriptomic insight into terpenoid and carbazole alkaloid biosynthesis, and functional characterization of two terpene synthases in curry tree (*Murraya koenigii*). *Sci. Rep.* **7**, 44126; doi: 10.1038/srep44126 (2017).

**Publisher's note:** Springer Nature remains neutral with regard to jurisdictional claims in published maps and institutional affiliations.

## Supplementary Material

Supplementary Information

## Figures and Tables

**Figure 1:**
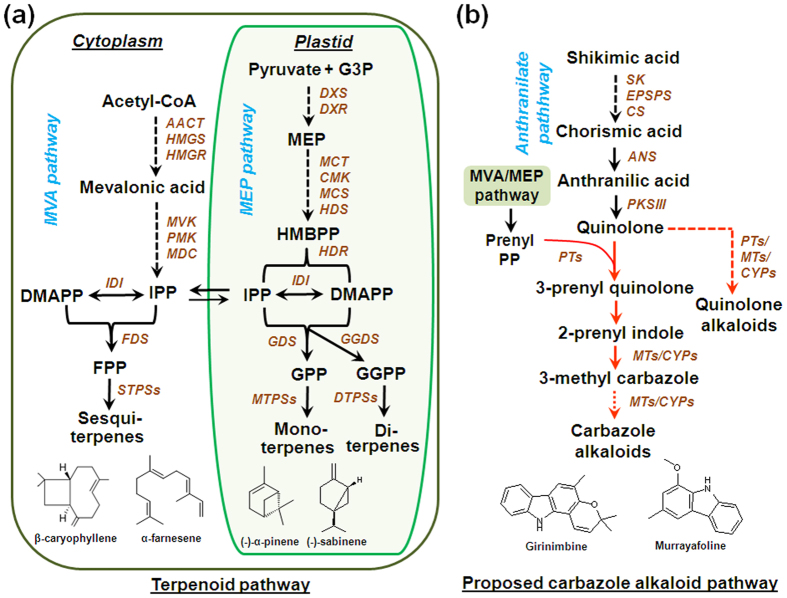
Biosynthetic pathways for terpenoids (**a**) and carbazole alkaloids (**b**) in *M. koenigii*. Abbreviations: *AACT*, acetoacetyl-CoA thiolase; *ANS*, anthranilate synthase; *CMK*, 4-(cytidine 5′-diphospho)-2-*C*-methyl-D-erythritol kinase; *CS*, chorismate synthase; *CYP450*, cytochrome P450; DMAPP, dimethylallyl pyrophosphate; *DTPSs,* diterpene synthases; *DXR*, 1-deoxy-D-xylulose-5-phosphate reductoisomerase; *DXS*, 1-deoxy-D-xylulose-5-phosphate synthase; *EPSPS*, 5-enolpyruvylshikimate-3-phosphate (EPSP) synthase; *FDS*, farnesyl diphosphate synthase; *FPP*, farnesyl diphosphate; G3P, 3-phosphoglyceraldehyde; *GDS*, geranyl diphosphate synthase; *GGDS*, geranylgeranyl diphosphate synthase; GGPP, geranylgeranyl pyrophosphate; GPP, geranyl pyrophosphate; *HDR*, hydroxy-2-methyl-2-(*E*)-butenyl 4-diphosphate reductase; *HDS*, hydroxy-2-methyl-2-(*E*)-butenyl 4-diphosphate synthase; HMBPP, (*E*)-4-hydroxy-3-methyl-but-2-enyl diphosphate; *HMGR*, 3-hydroxy-3-methyl glutaryl coenzyme A reductase; *HMGS*, 3-hydroxy-3 methyl glutaryl coenzyme A synthase; *IDI*, isopentenyl diphosphate isomerase; IPP, isopentenyl diphosphate; *MCT*, 2-*C*-methyl-D-erythritol-4-(cytidyl-5-diphosphate) transferase; *MCS*, 2-*C*-methyl-D-erythritol-2,4-cyclodiphosphate synthase; *MDC*, mevalonate-5-pyrophosphate decarboxylase; MEP, 2-*C*-methyl-D-erythritol 4-phosphate; *MTs*, methyltransferses; *MTPs*, mono terpene synthases; *MVK*, mevalonate kinase; *PMK*, 5-phosphomevalonate kinase; *PKSIII*; polyketide synthase type III; *PTs*, prenyltransferases; *SK,* shikimate kinase; S*TPSs,* sesqui terpene synthases. Dashed arrows represent multiple steps. Arrows marked in red are proposed steps in carbazole alkaloid pathway^3^. The chemical structures represent major volatile terpenes and carbazole alkaloids reported in *M. koenigii* leaf.

**Table 1:** Contig abundance of terpenoid and carbazole alkaloid pathway genes in *M. koenigii* leaf transcriptome.

Gene Name	Number of Contigs	Total FPKM	Full Length
Genes of MEP pathway			
*DXS*	13	297	8
*DXR*	17	1380	2
*MCT*	4	181	2
*CMK*	13	691	—
*MCDS*	2	83	1
*HDS*	5	341	4
*HDR*	*6*	440	*—*
*IDI*	11	833	—
**Genes of mono and di- terpene biosynthesis**			
*GDS*	15	292	1
*GGDS*	41	1906	5
*MTPS*	36	2828	3
*DTPS*	22	311	1
**Genes of MVA pathway**			
*ACAT*	11	193	3
*HMGS*	9	118	3
*HMGR*	4	15	4
*MVK*	3	18	—
*PMK*	5	73	2
*MVD*	11	92	—
**Genes of sesquiterpene biosynthesis**			
*FDS*	6	74	2
*STPS*	70	1928	3
**Genes of shikimate- derived carbazole alkaloid biosynthesis**			
*SK*	33	919	5
*EPSPS*	12	117	1
*CS*	16	1249	4
*ANS*	7	207	4
*PKS*	44	2169	4
