# Supplementary material for: Transcriptomic insight into terpenoid and carbazole alkaloid biosynthesis, and functional characterization of two terpene synthases in curry tree (Murraya koenigii)
Source: Sci Rep. 2017 Mar 8;7:44126. doi: 10.1038/srep44126 (PMC5341033; doi:10.1038/srep44126)
Supplement: Supplementary Information [file srep44126-s1.pdf]

Supplementary Information

Transcriptomic insight into terpenoid and carbazole-alkaloid biosynthesis, and functional characterization of two terpene synthases in curry tree (*Murraya koenigii* L.)

Seema Meena[1#], Sarma Rajeev Kumar[1#], Varun Dwivedi[1#], Anup Kumar Singh[1], Chandan S. Chanotiya[2], Md. Qussen Akhtar[3], Krishna Kumar[1], Ajit Kumar Shasany[3], and Dinesh A. Nagegowda[1*]

Table S1. List of biologically active compounds isolated from different plant parts of *M. koenigii* and their respective biological properties.

Biologically active compound	Part	Biological activity	References
1,1'-Bis(2-hydroxy-3-methylcarbazole)/3,3'-Dimethyl-[1,1'-bi-9H-carbazole]-2,2'-diol	Roots & Stem bark	-	1
1-formyl-3-methoxy-6-methyl carbazole<br>6,7-hydroxy-3-methylcarbazole	Leaves	antimicrobial	2
3e(1e-hydroxy ethyl)-7-hydroxy-1-isobenzofuranone<br>3,3'-[oxybis(methylene)]bis99-methoxy-9H-carbazole)	Stem bark	antibacterial, antidibetic	3, 4
3-methyl Carbazole	Roots	antimicrobial	5
8,8' '-biskoenigine (bisindole alkaloid)	Leaves	antiosteoporotic	6
9-Carbethoxy-3-methyl-9H-carbazole/Ethyl 3-methyl-9H-carbazole-9-carboxylate	Roots	antiinflammatory, antineoplastic	5
9-formyl-3-methylcarbazole	Roots	cytotoxic	5
Bicyclomahanimbicine	Leaves	antidiabetic, antioxidant, anticancer, antiulcer	7, 8
Bikoeniquinone-A (bisindole alkaloid)	Roots & Stem bark	-	1
Bikoeniquinonine (bisindole alkaloid)	Aerial part	antimicrobial	6, 9
Bismurrayaquinone-A	Roots & Stem bark	-	1
Bisgerayafolines A–C	Fruit pulp	antioxidant, anti-α-glucosidase, DNA binding, cytotoxic	10
Bismahanine	Leaves & Stem bark	antioxidant	1, 11
Bismahanimbinol	Fruit pulp	-	10
Bispyarafoline	Leaves & Fruit pulp	antioxidant	10, 11
Bismurrayafoline E	Leaves	antioxidant	10, 11
Bismurrayaquinone-A (bisindole alkaloid)	Roots & Stem bark	inotropic response (triphasic)	1, 12

Chrestifoline-A	Roots	cytotoxic	1
Curryangine/Mahanimbidine/Murrayazoline	Stem & Leaves	cytotoxic	13
Euchrestine-B	Leaves	antioxidant	10, 11
eustifoline-C	Roots	-	1
Curryanine/Murrayazolidine/Cyclomahanimbine (terpenoid alkaloid)	Leaves & Stem bark	antiinflammatory	7
Furostifoline	Stem	cytotoxic	13
Girinimbilol/mukoenine-A/2-Hydroxy-3-methyl-1-prenylcarbazole	Stem bark	anti-trichomonal	14, 15
Girinimbiol	Leaves	hypoglycaemic, hepatoprotective	16
Girinimbine/3,11-Dihydro-3,3,5-trimethyl-Pyrano(3,2-a)carbazole	Seeds, Leaves & stem bark	anti-trichomonal, hypoglycaemic, cytotoxic, antiplatelet agent	1, 15-17
Glycozoline	Roots	antibacterial	1
Isomahanine/Pyrafoline D/Pyrayafoline D	Leaves, Seeds & Fruits	antibacterial, cytotoxicity against HL-60 cells	18, 19
isomurrayafoline-B	Stem bark	analgesic activity	1, 20
Isomurrayazoline	Stem bark	anti-amnesic, immunomodulatory	21, 22
Isomurrayazolinine	Stem bark	-	21
karapinchamines A & B	Leaves	melanogenesis	23
Koenigine	Leaves	antioxidant, radical-scavenging	2, 24
Koenimbidine/Koenidine/Koenigicine	Roots & Leaves	insecticidal, antidiabetic	25, 26
Koenimbine	Seeds, Leaves & Stem	anti-diarrhea, antiinflammatory, antimicrobial	27, 28
Koenine	Leaves, seeds	anti-diarrhea	27
Koenoline/1-Methoxy-9H-carbazole-3-methanol	Roots & Stem bark	anti-tumor	29
Kurryam	seeds	anti-diarrhea	27
Mahanimbicine/Isomahanimbine	Roots & Leaves	anticancer, antimicrobial, mosquitocidal, antiproliferative, wound healing, antioxidant	11, 30

Mahanimbine	Roots, Leaves, Stem bark & Seeds	antidiabetic, hypolipidemic, antimicrobial, mosquitocidal, antioxidant, antiinflammatory, antiproliferative, anti-diarrhea, antitumor, wound healing	11, 25, 27, 30, 31
Mahanimbinine (terpenoid alkaloid)	Leaves & Seeds	antioxidant, anti-microbial	7, 8
Mahanimbilol	Stem bark	antitrichomal	14, 15
Mahanimbinol/1-(3,7-Dimethyl-octa-2,6-dienyl)-3-methyl-9H-carbazol-2-ol	Stem bark	cytotoxic	1
Mahanimboline	Roots	anti-diarrhea, antibacterial	27
Mahanine	Leaves, Roots, Stem bark, Seeds & Fruit pulp	anticancer, antioxidant, antimicrobial, mosquitocidal, antiinflammatory, antiproliferative, anti-diarrhea, wound healing, radical scavenging, α-glucosidase inhibitory	10, 11, 19, 27, 30
Mukonal/2-Hydroxy-9H-carbazole-3-carboxaldehyde	Stem bark	antifungal	32, 33
Mukoeic acid	Stem bark	antioxidant	34
Mukolidine/3-Formyl-8-methoxycarbazole/6-Formyl-1-methoxycarbazole	Roots	-	35
Mukoline/6-Hydroxymethyl-1-methoxycarbazole	Roots	antibacterial	35
Mukonicine	Leaves	antioxidant	36
Mukonidine	Stem bark	-	10
Mukoenine-B/3-Formyl-1-geranyl-2-hydroxycarbazole	roots	-	1
Mukoenine-C/Murrayamine A	roots	-	1
Mukoenigatin	aerial part	-	9
Murrafoline -A, B, C, D, G, H	-	-	1
Murrafoline-I	-	cytotoxicity against HL-60 cells	19
Murrayacine	Stem bark	-	25
Murrayacinine	Leaves	antioxidant, antimicrobial, antidiabetic	8

Murrayanine/1-Methoxy-9H-carbazole-3-carbaldehyde	Leaf & Stem bark	antimicrobial, neuroprotective, antiinflammatory	6, 37, 38
Murrayadinal	aerial part	-	9
Murrayanol	Leaves & Seeds	antimicrobial, antioxidant, mosquitocidal, antiinflammatory, anticancer	18, 39
Murrayafoline-A	Stem bark & Roots	anticancer	1, 40
Murrastifoline-F/1,1'-Dimethoxy-3,3'-dimethyl-4,9'-bi-9H-carbazole, 9CI	Roots	-	1
Murrayaquinone-A	Stem bark & Roots	anticancer	1, 40
Murrayaquinone-B	Stem bark	-	1
Murrayaquinoline	Stem	cytotoxic	13
Murrayazolinine	Stem bark	cytotoxic	13
Murrayazolinol	Roots & Stem bark	-	41
O-methyl murrayamine A	Leaves	-	10, 11
O-methyl mahanine	Leaves & Fruit pulp	-	10, 11
O-methyl mukanol	Leaves & Fruit pulp		10, 11
Scopolin	Leaves	antiinflammatory, anti-dementia	38
Xanthotoxin	Seeds	used to treat psoriasis, eczema, vitiligo & some cutaneous lymphomas	42
Isobyakangelicol	Seeds	-	42
Phellopterin	Seeds	neuroprotective	42
Gosferol	Seeds	-	42
Neobyakangelicol	Seeds	-	42
Byakangelicol	Seeds	-	42
Byakangelicin	Seeds	Antidiabetic & anticataract	42
Isogosferol	Seeds	-	42

Table S2. Summary of RNA-Seq.

	Murraya koenigii
**Total Number of HQ Reads**	**50311257 (50.31 Mb)**
**Number of paired-end reads after trimming/filtered**	**46517951(46.51 Mb)**
**Mean read quality (Phred score)**	**35.415**
**Number of bases (MB)**	**5081.44**
**Number of bases (Gb) after trimming**	**3.72**
**Mean read length (bp)**	**101**
**kmer size**	**41**
**Number of assembled transcripts**	**118,221**
**No. of transcripts with length >= 150 bp**	**113,931**
**Maximum transcript length (bp)**	**12,154**
**Mean GC % of transcripts**	**40.79**
**Number of transcripts with FPKM >= 1.0**	**102,857**
**N50 value**	**1733**

Table S3: Annotation summary of *M. koenigii* leaf transcriptome.

Abbriviation: TNAT-Total number of annotated transcripts.

Database	TNAT	Percentage(%)
NCBI	87,417	84.98
Uniprot	54,887	53.36
KEGG	30708	29.94
AGRIS	8261	8.03
Total transcripts	102857	100

Table S4. Trancripts for prenyltransferase gene family with unknown function identified in *M. koenigii* leaf transcriptome.
NA- Not annotated

Transcript ID	Read count	FPKM	Gene name	Organism	GO term
Locus_9584_Transcript_4/8_Confidence_0.333_Length_920	381	8.90259	hypothetical protein CICLE_v10000458mg	*Citrus clementina*	protein prenylation; protein prenyltransferase activity
Locus_10147_Transcript_10/11_Confidence_0.188_Length_747	160	4.60446	hypothetical protein CICLE_v10020293mg	*Citrus clementina*	protein prenylation; protein prenyltransferase activity
Locus_10147_Transcript_11/11_Confidence_0.062_Length_747	158	4.54691	hypothetical protein CICLE_v10020293mg	*Citrus clementina*	protein prenylation; protein prenyltransferase activity
Locus_10147_Transcript_7/11_Confidence_0.438_Length_1027	255	5.33764	hypothetical protein CICLE_v10020293mg	*Citrus clementina*	protein prenylation; protein prenyltransferase activity
Locus_10147_Transcript_8/11_Confidence_0.250_Length_1218	250	4.41237	hypothetical protein CICLE_v10020293mg	*Citrus clementina*	protein prenylation; protein prenyltransferase activity
Locus_10311_Transcript_1/5_Confidence_0.667_Length_1420	1801	27.265	hypothetical protein CICLE_v10011942mg	*Citrus clementina*	integral component of membrane; prenyltransferase activity
Locus_10311_Transcript_2/5_Confidence_0.667_Length_1494	1966	28.2887	hypothetical protein CICLE_v10011942mg	*Citrus clementina*	integral component of membrane; prenyltransferase activity
Locus_2984_Transcript_4/4_Confidence_0.222_Length_574	131	4.90613	hypothetical protein CICLE_v10020293mg	*Citrus clementina*	protein prenylation; protein prenyltransferase activity
Locus_565_Transcript_12/20_Confidence_0.484_Length_1409	2635	40.2021	prenyltransferase	*Populus trichocarpa*	NA
Locus_7997_Transcript_1/5_Confidence_0.833_Length_689	513	16.0058	prenyltransferase	*Populus trichocarpa*	NA
Locus_7997_Transcript_2/5_Confidence_0.667_Length_651	485	16.0155	prenyltransferase	*Populus trichocarpa*	NA
Locus_7997_Transcript_3/5_Confidence_0.500_Length_617	433	15.0863	prenyltransferase	*Populus trichocarpa*	NA
Locus_7997_Transcript_4/5_Confidence_0.667_Length_1435	493	7.38541	prenyltransferase	*Populus trichocarpa*	NA
Locus_7997_Transcript_5/5_Confidence_0.167_Length_1577	543	7.40197	prenyltransferase	*Populus trichocarpa*	NA

Table S5. Transcripts for methyltransferase gene family with unknown function identified in *M. koenigii* leaf transcriptome. NA- Not annotated.

Transcript ID	Read count	FPKM	Gene name	Organism	GO term
Locus_14513_Transcript_1/1_Confidence_0.000_Length_1760	281	3.4322	Methyltransferase	*Theobroma cacao*	NA
Locus_30864_Transcript_1/1_Confidence_0.000_Length_272	13	1.02743	Methyltransferase	*Ajellomyces dermatitidis* ATCC 18188	NA
Locus_22721_Transcript_1/1_Confidence_0.000_Length_836	57	1.46571	Methyltransferase family protein, putative	*Theobroma cacao*	NA
Locus_22721_Transcript_1/1_Confidence_0.000_Length_836	57	1.46571	Methyltransferase family protein, putative	*Theobroma cacao*	NA
Locus_4840_Transcript_10/28_Confidence_0.343_Length_2009	670	7.16926	Methyltransferase family protein isoform 2	*Theobroma cacao*	NA
Locus_4840_Transcript_11/28_Confidence_0.314_Length_1923	639	7.14333	Methyltransferase family protein isoform 2	*Theobroma cacao*	NA
Locus_4840_Transcript_13/28_Confidence_0.171_Length_569	370	13.9788	Methyltransferase family protein isoform 2	*Theobroma cacao*	NA
Locus_4840_Transcript_14/28_Confidence_0.171_Length_572	367	13.7927	Methyltransferase family protein isoform 2	*Theobroma cacao*	NA
Locus_4840_Transcript_17/28_Confidence_0.343_Length_2125	633	6.4036	Methyltransferase family protein isoform 2	*Theobroma cacao*	NA
Locus_4840_Transcript_18/28_Confidence_0.114_Length_685	334	10.4818	Methyltransferase family protein isoform 2	*Theobroma cacao*	NA
Locus_4840_Transcript_24/28_Confidence_0.314_Length_1107	451	8.75807	Methyltransferase family protein isoform 2	*Theobroma cacao*	NA
Locus_4840_Transcript_25/28_Confidence_0.086_Length_1392	453	6.99582	Methyltransferase family protein isoform 2	*Theobroma cacao*	NA
Locus_4840_Transcript_22/28_Confidence_0.171_Length_701	163	4.99861	Methyltransferase family protein isoform 4	*Theobroma cacao*	NA
Locus_4840_Transcript_23/28_Confidence_0.171_Length_1126	203	3.87558	Methyltransferase family protein isoform 4	*Theobroma cacao*	NA
Locus_7879_Transcript_2/3_Confidence_0.400_Length_919	530	12.3977	PREDICTED: putative methyltransferase At1g22800-like	*Citrus sinensis*	NA

Locus_14985_Transcript_1/4_Confidence_0.500_Length_570	115	4.33713	PREDICTED: putative methyltransferase At1g22800-like	*Citrus sinensis*	NA
Locus_14985_Transcript_2/4_Confidence_0.333_Length_540	108	4.29942	PREDICTED: putative methyltransferase At1g22800-like	*Citrus sinensis*	NA
Locus_14985_Transcript_3/4_Confidence_0.500_Length_595	105	3.7936	PREDICTED: putative methyltransferase At1g22800-like	*Citrus sinensis*	NA
Locus_14985_Transcript_4/4_Confidence_0.000_Length_660	108	3.5177	PREDICTED: putative methyltransferase At1g22800-like	*Citrus sinensis*	NA
Locus_2014_Transcript_6/12_Confidence_0.067_Length_199	18	1.94446	PREDICTED: uncharacterized methyltransferase WBSCR22-like	*Cucumis sativus*	NA
Locus_10007_Transcript_1/7_Confidence_0.333_Length_1091	1975	38.9154	PREDICTED: uncharacterized methyltransferase At1g78140, chloroplastic-like isoform X1	*Citrus sinensis*	NA
Locus_10007_Transcript_3/7_Confidence_0.667_Length_1604	2196	29.4312	PREDICTED: uncharacterized methyltransferase At1g78140, chloroplastic-like isoform X1	*Citrus sinensis*	NA
Locus_10007_Transcript_4/7_Confidence_0.583_Length_1709	2172	27.321	PREDICTED: uncharacterized methyltransferase At1g78140, chloroplastic-like isoform X1	*Citrus sinensis*	NA
Locus_10007_Transcript_5/7_Confidence_0.583_Length_1694	2034	25.8117	PREDICTED: uncharacterized methyltransferase At1g78140, chloroplastic-like isoform X1	*Citrus sinensis*	NA
Locus_10007_Transcript_6/7_Confidence_0.417_Length_1064	639	12.9104	PREDICTED: uncharacterized methyltransferase At1g78140, chloroplastic-like isoform X1	*Citrus sinensis*	NA
Locus_10007_Transcript_7/7_Confidence_0.250_Length_1604	2199	29.4714	PREDICTED: uncharacterized methyltransferase At1g78140, chloroplastic-like isoform X1	*Citrus sinensis*	NA
Locus_10007_Transcript_2/7_Confidence_0.167_Length_670	1309	41.9995	PREDICTED: uncharacterized methyltransferase At1g78140, chloroplastic-like isoform X2	*Citrus sinensis*	NA
Locus_2847_Transcript_10/29_Confidence_0.289_Length_2063	6881	71.7021	PREDICTED: uncharacterized methyltransferase At2g41040, chloroplastic-like	*Citrus sinensis*	NA
Locus_2847_Transcript_11/29_Confidence_0.200_Length_1165	3223	59.4722	PREDICTED: uncharacterized methyltransferase At2g41040, chloroplastic-like	*Citrus sinensis*	NA
Locus_2847_Transcript_12/29_Confidence_0.222_Length_1205	4544	81.0645	PREDICTED: uncharacterized methyltransferase At2g41040, chloroplastic-like	*Citrus sinensis*	NA
Locus_2847_Transcript_2/29_Confidence_0.133_Length_948	2382	54.0148	PREDICTED: uncharacterized methyltransferase At2g41040, chloroplastic-like	*Citrus sinensis*	NA
Locus_2847_Transcript_3/29_Confidence_0.222_Length_936	5215	119.773	PREDICTED: uncharacterized methyltransferase At2g41040, chloroplastic-like	*Citrus sinensis*	NA
Locus_2847_Transcript_4/29_Confidence_0.267_Length_1039	5481	113.403	PREDICTED: uncharacterized methyltransferase At2g41040, chloroplastic-like	*Citrus sinensis*	NA

Locus_2847_Transcript_5/29_Confidence_0.289_Length_1204	5877	104.932	PREDICTED: uncharacterized methyltransferase At2g41040, chloroplastic-like	*Citrus sinensis*	NA
Locus_2847_Transcript_6/29_Confidence_0.289_Length_1973	6640	72.347	PREDICTED: uncharacterized methyltransferase At2g41040, chloroplastic-like	*Citrus sinensis*	NA
Locus_2847_Transcript_7/29_Confidence_0.289_Length_1952	5393	59.3923	PREDICTED: uncharacterized methyltransferase At2g41040, chloroplastic-like	*Citrus sinensis*	NA
Locus_2847_Transcript_9/29_Confidence_0.311_Length_1294	6120	101.671	PREDICTED: uncharacterized methyltransferase At2g41040, chloroplastic-like	*Citrus sinensis*	NA

Table S6a: Cytochrome P450 families identified in *M. koenigii* leaf transcriptome putatively involved in Secondary metabolic pathways

CYP Family	Locus
CYP51	Locus_7540_Transcript_1/1_Confidence_0.000_Length_1583
	Locus_31208_Transcript_1/2_Confidence_0.333_Length_398
CYP70	Locus_21790_Transcript_3/3_Confidence_0.000_Length_307
CYP71	Locus_2163_Transcript_6/11_Confidence_0.269_Length_721
	Locus_2163_Transcript_10/11_Confidence_0.385_Length_1465
	Locus_2163_Transcript_1/11_Confidence_0.269_Length_681
	Locus_7712_Transcript_6/10_Confidence_0.455_Length_726
	Locus_3183_Transcript_12/23_Confidence_0.129_Length_663
	Locus_2658_Transcript_11/69_Confidence_0.035_Length_241
	Locus_21900_Transcript_1/1_Confidence_0.000_Length_150
	Locus_16873_Transcript_1/2_Confidence_0.333_Length_467
	Locus_2312_Transcript_4/9_Confidence_0.417_Length_2270
	Locus_2312_Transcript_3/9_Confidence_0.458_Length_2289
	Locus_2312_Transcript_1/9_Confidence_0.458_Length_2300
	Locus_2312_Transcript_5/9_Confidence_0.208_Length_2261
	Locus_2312_Transcript_2/9_Confidence_0.458_Length_2411
	Locus_24492_Transcript_1/1_Confidence_0.000_Length_305
	Locus_9952_Transcript_4/4_Confidence_0.000_Length_910
	Locus_19197_Transcript_1/1_Confidence_0.000_Length_425
	Locus_3026_Transcript_55/74_Confidence_0.028_Length_342
	Locus_3356_Transcript_3/48_Confidence_0.074_Length_3952
	Locus_3356_Transcript_8/48_Confidence_0.037_Length_4106
	Locus_3356_Transcript_4/48_Confidence_0.025_Length_2472
	Locus_2658_Transcript_8/69_Confidence_0.053_Length_357
	Locus_2658_Transcript_9/69_Confidence_0.053_Length_391

	Locus_2658_Transcript_37/69_Confidence_0.053_Length_523
	Locus_2658_Transcript_7/69_Confidence_0.041_Length_593
	Locus_2658_Transcript_41/69_Confidence_0.058_Length_938
	Locus_37231_Transcript_1/1_Confidence_1.000_Length_220
	Locus_2658_Transcript_1/69_Confidence_0.029_Length_667
	Locus_2658_Transcript_2/69_Confidence_0.023_Length_352
	Locus_2658_Transcript_3/69_Confidence_0.023_Length_456
	Locus_2658_Transcript_10/69_Confidence_0.041_Length_299
	Locus_2656_Transcript_2/3_Confidence_0.455_Length_813
	Locus_2656_Transcript_1/3_Confidence_0.182_Length_447
	Locus_2658_Transcript_52/69_Confidence_0.041_Length_228
	Locus_2658_Transcript_64/69_Confidence_0.292_Length_3126
	Locus_2658_Transcript_25/69_Confidence_0.135_Length_1122
	Locus_2658_Transcript_67/69_Confidence_0.222_Length_3336
	Locus_2658_Transcript_61/69_Confidence_0.251_Length_3206
	Locus_2658_Transcript_4/69_Confidence_0.070_Length_321
	Locus_2658_Transcript_5/69_Confidence_0.058_Length_288
	Locus_3026_Transcript_52/74_Confidence_0.028_Length_578
	Locus_3026_Transcript_53/74_Confidence_0.043_Length_732
	Locus_11455_Transcript_1/1_Confidence_0.000_Length_1818
	Locus_37069_Transcript_1/1_Confidence_0.667_Length_170
	Locus_29371_Transcript_1/1_Confidence_0.000_Length_407
	Locus_8228_Transcript_1/1_Confidence_0.000_Length_1766
CYP72	Locus_7712_Transcript_7/10_Confidence_0.409_Length_670
	Locus_7712_Transcript_10/10_Confidence_0.273_Length_1276
	Locus_7712_Transcript_8/10_Confidence_0.500_Length_1274
	Locus_2163_Transcript_4/11_Confidence_0.346_Length_684
	Locus_7712_Transcript_2/10_Confidence_0.500_Length_852
	Locus_7712_Transcript_1/10_Confidence_0.500_Length_850
	Locus_2163_Transcript_5/11_Confidence_0.308_Length_699

	**Locus_7712_Transcript_9/10_Confidence_0.545_Length_965**
	**Locus_7712_Transcript_5/10_Confidence_0.409_Length_1394**
	**Locus_7712_Transcript_4/10_Confidence_0.500_Length_1392**
	**Locus_2163_Transcript_2/11_Confidence_0.269_Length_830**
	**Locus_2843_Transcript_18/18_Confidence_0.200_Length_3790**
	**Locus_2843_Transcript_17/18_Confidence_0.533_Length_3870**
	**Locus_1265_Transcript_5/69_Confidence_0.059_Length_929**
	**Locus_1265_Transcript_2/69_Confidence_0.034_Length_581**
	**Locus_1265_Transcript_61/69_Confidence_0.109_Length_1394**
	**Locus_9283_Transcript_2/2_Confidence_0.000_Length_1221**
	**Locus_1265_Transcript_37/69_Confidence_0.034_Length_515**
	**Locus_1265_Transcript_46/69_Confidence_0.084_Length_866**
	**Locus_1265_Transcript_43/69_Confidence_0.059_Length_847**
	**Locus_27095_Transcript_1/1_Confidence_0.000_Length_405**
	**Locus_1265_Transcript_41/69_Confidence_0.059_Length_637**
	**Locus_1265_Transcript_39/69_Confidence_0.076_Length_637**
	**Locus_1265_Transcript_35/69_Confidence_0.076_Length_649**
	**Locus_1265_Transcript_36/69_Confidence_0.076_Length_675**
	**Locus_1265_Transcript_40/69_Confidence_0.076_Length_759**
	**Locus_1265_Transcript_1/69_Confidence_0.034_Length_831**
	**Locus_1265_Transcript_3/69_Confidence_0.042_Length_839**
	**Locus_1265_Transcript_56/69_Confidence_0.059_Length_554**
	**Locus_1265_Transcript_66/69_Confidence_0.067_Length_424**
	**Locus_1265_Transcript_4/69_Confidence_0.025_Length_580**
	**Locus_1265_Transcript_55/69_Confidence_0.059_Length_918**
	**Locus_1265_Transcript_51/69_Confidence_0.126_Length_961**
	**Locus_1265_Transcript_33/69_Confidence_0.109_Length_1413**
	**Locus_1265_Transcript_32/69_Confidence_0.118_Length_1569**
	**Locus_23869_Transcript_1/1_Confidence_0.000_Length_221**
	**Locus_27253_Transcript_1/1_Confidence_0.000_Length_396**

	**Locus_1265_Transcript_60/69_Confidence_0.025_Length_469**
	**Locus_1265_Transcript_57/69_Confidence_0.042_Length_1162**
	**Locus_1265_Transcript_63/69_Confidence_0.067_Length_424**
	**Locus_1265_Transcript_67/69_Confidence_0.050_Length_424**
	**Locus_1265_Transcript_65/69_Confidence_0.050_Length_670**
	**Locus_3026_Transcript_15/74_Confidence_0.043_Length_449**
	**Locus_3026_Transcript_14/74_Confidence_0.014_Length_449**
	**Locus_3026_Transcript_31/74_Confidence_0.014_Length_511**
	**Locus_3026_Transcript_27/74_Confidence_0.043_Length_757**
	**Locus_3026_Transcript_28/74_Confidence_0.035_Length_757**
	**Locus_1900_Transcript_12/104_Confidence_0.040_Length_1345**
	**Locus_1900_Transcript_13/104_Confidence_0.015_Length_919**
	**Locus_9283_Transcript_1/2_Confidence_0.333_Length_1824**
**CYP73**	**Locus_5435_Transcript_3/3_Confidence_0.000_Length_865**
	**Locus_5435_Transcript_2/3_Confidence_0.333_Length_1897**
	**Locus_5435_Transcript_1/3_Confidence_0.667_Length_1101**
	**Locus_25834_Transcript_1/1_Confidence_0.000_Length_811**
	**Locus_32754_Transcript_1/1_Confidence_0.000_Length_506**
	**Locus_30656_Transcript_1/1_Confidence_0.000_Length_217**
	**Locus_32312_Transcript_1/1_Confidence_0.000_Length_161**
**CYP74**	**Locus_2093_Transcript_5/5_Confidence_0.111_Length_1720**
	**Locus_2093_Transcript_4/5_Confidence_0.333_Length_2108**
	**Locus_2093_Transcript_3/5_Confidence_0.333_Length_2201**
	**Locus_2093_Transcript_2/5_Confidence_0.222_Length_998**
	**Locus_2093_Transcript_1/5_Confidence_0.111_Length_1136**
	**Locus_11826_Transcript_1/4_Confidence_0.714_Length_2049**
	**Locus_11826_Transcript_4/4_Confidence_0.143_Length_2049**
	**Locus_11826_Transcript_2/4_Confidence_0.714_Length_2061**
	**Locus_11826_Transcript_3/4_Confidence_0.429_Length_1919**
**CYP75**	**Locus_6034_Transcript_6/15_Confidence_0.600_Length_2103**

	**Locus_31518_Transcript_1/1_Confidence_0.000_Length_381**
	**Locus_6034_Transcript_12/15_Confidence_0.320_Length_1242**
	**Locus_6034_Transcript_8/15_Confidence_0.560_Length_1504**
	**Locus_6034_Transcript_14/15_Confidence_0.280_Length_1817**
	**Locus_21489_Transcript_1/1_Confidence_0.000_Length_203**
	**Locus_16409_Transcript_3/4_Confidence_0.500_Length_1727**
	**Locus_16409_Transcript_1/4_Confidence_0.750_Length_1728**
	**Locus_16409_Transcript_4/4_Confidence_0.000_Length_1652**
	**Locus_16409_Transcript_2/4_Confidence_0.250_Length_1653**
	**Locus_11745_Transcript_2/2_Confidence_0.000_Length_1803**
	**Locus_11745_Transcript_1/2_Confidence_0.750_Length_1596**
**CYP76**	**Locus_384_Transcript_17/34_Confidence_0.068_Length_507**
	**Locus_384_Transcript_4/34_Confidence_0.095_Length_215**
	Locus_384_Transcript_8/34_Confidence_0.027_Length_518
	**Locus_22631_Transcript_1/1_Confidence_0.000_Length_257**
	**Locus_384_Transcript_22/34_Confidence_0.095_Length_540**
	**Locus_384_Transcript_23/34_Confidence_0.149_Length_679**
	**Locus_384_Transcript_27/34_Confidence_0.135_Length_683**
	**Locus_13518_Transcript_1/2_Confidence_0.750_Length_1779**
	**Locus_384_Transcript_5/34_Confidence_0.216_Length_664**
	**Locus_384_Transcript_6/34_Confidence_0.162_Length_731**
	**Locus_384_Transcript_11/34_Confidence_0.257_Length_846**
	**Locus_384_Transcript_10/34_Confidence_0.257_Length_854**
	**Locus_384_Transcript_3/34_Confidence_0.095_Length_461**
	**Locus_384_Transcript_12/34_Confidence_0.203_Length_846**
	**Locus_384_Transcript_14/34_Confidence_0.216_Length_910**
	**Locus_13518_Transcript_2/2_Confidence_0.000_Length_1850**
	**Locus_18083_Transcript_1/2_Confidence_1.000_Length_266**
**CYP78**	**Locus_12021_Transcript_1/1_Confidence_0.000_Length_564**
	**Locus_10475_Transcript_5/11_Confidence_0.500_Length_2157**

	**Locus_10475_Transcript_7/11_Confidence_0.188_Length_1295**
	**Locus_10475_Transcript_9/11_Confidence_0.125_Length_1785**
	**Locus_6692_Transcript_2/2_Confidence_0.333_Length_810**
	**Locus_6692_Transcript_1/2_Confidence_0.667_Length_803**
	**Locus_21638_Transcript_1/1_Confidence_0.000_Length_701**
	**Locus_12984_Transcript_1/1_Confidence_0.000_Length_728**
**CYP79**	**Locus_10851_Transcript_10/11_Confidence_0.476_Length_1586**
	**Locus_3183_Transcript_1/23_Confidence_0.065_Length_379**
	**Locus_5093_Transcript_3/4_Confidence_0.400_Length_2576**
	**Locus_5093_Transcript_2/4_Confidence_0.600_Length_2353**
	**Locus_22028_Transcript_1/1_Confidence_0.000_Length_297**
**CYP81**	**Locus_3183_Transcript_2/23_Confidence_0.032_Length_473**
	**Locus_6868_Transcript_3/14_Confidence_0.333_Length_492**
	**Locus_6868_Transcript_2/14_Confidence_0.400_Length_525**
	**Locus_6868_Transcript_1/14_Confidence_0.467_Length_850**
	**Locus_2612_Transcript_4/4_Confidence_0.000_Length_1036**
	**Locus_2612_Transcript_3/4_Confidence_0.333_Length_1908**
	**Locus_1752_Transcript_1/7_Confidence_0.111_Length_1903**
	**Locus_1752_Transcript_2/7_Confidence_0.111_Length_2104**
	**Locus_11470_Transcript_3/4_Confidence_0.571_Length_1398**
	**Locus_24190_Transcript_1/1_Confidence_0.000_Length_1129**
	**Locus_11470_Transcript_2/4_Confidence_0.429_Length_1398**
	**Locus_11470_Transcript_4/4_Confidence_0.000_Length_1398**
	**Locus_7159_Transcript_5/7_Confidence_0.667_Length_1917**
	**Locus_7159_Transcript_6/7_Confidence_0.500_Length_1917**
	**Locus_7159_Transcript_4/7_Confidence_0.250_Length_1327**
	**Locus_11470_Transcript_1/4_Confidence_0.286_Length_270**
	**Locus_7159_Transcript_2/7_Confidence_0.500_Length_1195**
	**Locus_7159_Transcript_3/7_Confidence_0.417_Length_1204**
	**Locus_7159_Transcript_1/7_Confidence_0.500_Length_1172**

	**Locus_1500_Transcript_8/8_Confidence_0.429_Length_2594**
	**Locus_1752_Transcript_6/7_Confidence_0.333_Length_1763**
	**Locus_1752_Transcript_5/7_Confidence_0.444_Length_1767**
	**Locus_1752_Transcript_3/7_Confidence_0.444_Length_3510**
	**Locus_12542_Transcript_1/9_Confidence_0.500_Length_580**
**CYP82**	**Locus_10475_Transcript_2/11_Confidence_0.188_Length_1306**
	**Locus_10475_Transcript_3/11_Confidence_0.062_Length_1602**
	**Locus_2006_Transcript_6/27_Confidence_0.283_Length_1221**
	**Locus_2006_Transcript_8/27_Confidence_0.264_Length_1352**
	**Locus_17484_Transcript_1/1_Confidence_0.000_Length_678**
	**Locus_2006_Transcript_16/27_Confidence_0.057_Length_380**
	**Locus_2006_Transcript_3/27_Confidence_0.189_Length_737**
	**Locus_2616_Transcript_5/5_Confidence_0.000_Length_1077**
	**Locus_2616_Transcript_4/5_Confidence_0.375_Length_1917**
	**Locus_2616_Transcript_1/5_Confidence_0.625_Length_1907**
	**Locus_2616_Transcript_3/5_Confidence_0.250_Length_1851**
	**Locus_2616_Transcript_2/5_Confidence_0.375_Length_1915**
	**Locus_2006_Transcript_12/27_Confidence_0.113_Length_628**
	**Locus_2006_Transcript_10/27_Confidence_0.226_Length_832**
	**Locus_2006_Transcript_11/27_Confidence_0.189_Length_672**
	**Locus_2096_Transcript_1/9_Confidence_0.182_Length_1978**
	**Locus_2096_Transcript_2/9_Confidence_0.091_Length_1980**
	**Locus_2006_Transcript_25/27_Confidence_0.321_Length_1534**
	**Locus_2006_Transcript_26/27_Confidence_0.226_Length_1571**
	**Locus_2006_Transcript_19/27_Confidence_0.396_Length_1572**
	**Locus_2006_Transcript_22/27_Confidence_0.358_Length_1572**
	**Locus_2006_Transcript_18/27_Confidence_0.396_Length_1565**
	**Locus_2006_Transcript_23/27_Confidence_0.396_Length_1641**
	**Locus_2006_Transcript_21/27_Confidence_0.396_Length_1659**
	**Locus_2006_Transcript_27/27_Confidence_0.113_Length_622**

	**Locus_2006_Transcript_4/27_Confidence_0.302_Length_1221**
	**Locus_2006_Transcript_2/27_Confidence_0.321_Length_1308**
	**Locus_16804_Transcript_1/1_Confidence_0.000_Length_905**
	**Locus_2006_Transcript_20/27_Confidence_0.340_Length_1572**
	**Locus_1496_Transcript_5/16_Confidence_0.625_Length_1627**
	**Locus_1496_Transcript_13/16_Confidence_0.583_Length_1641**
	**Locus_1496_Transcript_11/16_Confidence_0.625_Length_1641**
	**Locus_1496_Transcript_6/16_Confidence_0.542_Length_1627**
	**Locus_1496_Transcript_12/16_Confidence_0.500_Length_1641**
	**Locus_1496_Transcript_8/16_Confidence_0.625_Length_1695**
	**Locus_1496_Transcript_14/16_Confidence_0.542_Length_1709**
	**Locus_1496_Transcript_10/16_Confidence_0.542_Length_1811**
	**Locus_1496_Transcript_7/16_Confidence_0.625_Length_1626**
	**Locus_1496_Transcript_15/16_Confidence_0.333_Length_1640**
	**Locus_1496_Transcript_9/16_Confidence_0.583_Length_1575**
	**Locus_1496_Transcript_16/16_Confidence_0.000_Length_395**
	**Locus_1496_Transcript_3/16_Confidence_0.542_Length_1610**
	**Locus_1496_Transcript_4/16_Confidence_0.417_Length_1195**
	**Locus_1496_Transcript_1/16_Confidence_0.167_Length_828**
	**Locus_1496_Transcript_2/16_Confidence_0.042_Length_500**
	**Locus_2006_Transcript_5/27_Confidence_0.302_Length_1222**
**CYP83**	**Locus_3026_Transcript_71/74_Confidence_0.050_Length_1496**
	**Locus_3026_Transcript_67/74_Confidence_0.135_Length_2218**
	**Locus_3026_Transcript_62/74_Confidence_0.135_Length_2050**
	**Locus_13963_Transcript_5/5_Confidence_0.000_Length_1807**
	**Locus_13963_Transcript_4/5_Confidence_0.500_Length_1808**
	**Locus_13963_Transcript_1/5_Confidence_0.667_Length_1809**
	**Locus_13963_Transcript_3/5_Confidence_0.583_Length_1809**
	**Locus_13963_Transcript_2/5_Confidence_0.583_Length_1762**
	**Locus_2658_Transcript_36/69_Confidence_0.082_Length_1040**

	Locus_2658_Transcript_45/69_Confidence_0.111_Length_1318
	Locus_2658_Transcript_46/69_Confidence_0.064_Length_1329
	Locus_2658_Transcript_40/69_Confidence_0.082_Length_1401
	Locus_2658_Transcript_42/69_Confidence_0.111_Length_1401
	Locus_3026_Transcript_2/74_Confidence_0.064_Length_1006
	Locus_2658_Transcript_6/69_Confidence_0.058_Length_424
	Locus_37046_Transcript_1/1_Confidence_0.833_Length_338
CYP84	Locus_19681_Transcript_1/1_Confidence_0.000_Length_1840
	Locus_11698_Transcript_1/1_Confidence_0.000_Length_841
	Locus_17506_Transcript_1/1_Confidence_0.000_Length_401
CYP85	Locus_25051_Transcript_1/1_Confidence_0.000_Length_246
CYP86	Locus_2860_Transcript_4/16_Confidence_0.273_Length_758
	Locus_19470_Transcript_1/1_Confidence_0.000_Length_901
CYP87	Locus_11181_Transcript_10/17_Confidence_0.130_Length_381
	Locus_11181_Transcript_13/17_Confidence_0.174_Length_1410
	Locus_11181_Transcript_12/17_Confidence_0.174_Length_1415
	Locus_11181_Transcript_11/17_Confidence_0.174_Length_1410
	Locus_11181_Transcript_14/17_Confidence_0.130_Length_1372
	Locus_11181_Transcript_15/17_Confidence_0.043_Length_1338
CYP88	Locus_6215_Transcript_5/5_Confidence_0.000_Length_1741
	Locus_6215_Transcript_4/5_Confidence_0.125_Length_2038
CYP89	Locus_2860_Transcript_5/16_Confidence_0.273_Length_726
	Locus_2860_Transcript_6/16_Confidence_0.273_Length_812
	Locus_2860_Transcript_7/16_Confidence_0.318_Length_904
	Locus_2860_Transcript_15/16_Confidence_0.273_Length_828
	Locus_2860_Transcript_8/16_Confidence_0.545_Length_1268
	Locus_2860_Transcript_11/16_Confidence_0.318_Length_1035
	Locus_2860_Transcript_13/16_Confidence_0.500_Length_1358
	Locus_2860_Transcript_10/16_Confidence_0.500_Length_1354
	Locus_2860_Transcript_16/16_Confidence_0.227_Length_1370

	**Locus_2860_Transcript_14/16_Confidence_0.545_Length_1446**
	**Locus_2860_Transcript_9/16_Confidence_0.636_Length_1462**
	**Locus_2860_Transcript_12/16_Confidence_0.500_Length_1444**
	**Locus_3183_Transcript_18/23_Confidence_0.419_Length_1290**
	**Locus_10238_Transcript_1/1_Confidence_0.333_Length_1883**
	**Locus_16923_Transcript_3/4_Confidence_0.400_Length_1016**
	**Locus_16923_Transcript_1/4_Confidence_0.400_Length_852**
	**Locus_16923_Transcript_2/4_Confidence_0.200_Length_955**
	**Locus_10856_Transcript_3/7_Confidence_0.091_Length_628**
	**Locus_4058_Transcript_7/12_Confidence_0.143_Length_555**
**CYP90**	**Locus_296_Transcript_2/14_Confidence_0.200_Length_789**
	**Locus_296_Transcript_3/14_Confidence_0.333_Length_1223**
	**Locus_296_Transcript_14/14_Confidence_0.100_Length_1314**
	**Locus_296_Transcript_10/14_Confidence_0.300_Length_2127**
	**Locus_296_Transcript_13/14_Confidence_0.233_Length_2259**
	**Locus_296_Transcript_11/14_Confidence_0.367_Length_2384**
	**Locus_296_Transcript_7/14_Confidence_0.467_Length_2388**
	**Locus_296_Transcript_8/14_Confidence_0.433_Length_2415**
	**Locus_296_Transcript_9/14_Confidence_0.300_Length_1930**
	**Locus_296_Transcript_12/14_Confidence_0.100_Length_2867**
	**Locus_16943_Transcript_1/1_Confidence_0.000_Length_2055**
	**Locus_2919_Transcript_6/8_Confidence_0.200_Length_1003**
	**Locus_2919_Transcript_3/8_Confidence_0.400_Length_1573**
	**Locus_7472_Transcript_3/3_Confidence_0.000_Length_846**
	**Locus_7472_Transcript_1/3_Confidence_0.400_Length_2057**
	**Locus_7472_Transcript_2/3_Confidence_0.000_Length_1334**
	**Locus_21810_Transcript_1/1_Confidence_0.000_Length_535**
	**Locus_26673_Transcript_1/1_Confidence_0.000_Length_238**
	**Locus_9617_Transcript_5/6_Confidence_0.571_Length_1910**
	**Locus_9617_Transcript_4/6_Confidence_0.571_Length_1919**

	**Locus_9617_Transcript_3/6_Confidence_0.571_Length_2005**
	**Locus_9617_Transcript_1/6_Confidence_0.714_Length_2014**
	**Locus_9617_Transcript_6/6_Confidence_0.143_Length_2014**
	**Locus_9617_Transcript_2/6_Confidence_0.714_Length_2109**
**CYP94**	**Locus_2871_Transcript_1/2_Confidence_0.750_Length_1863**
	**Locus_2871_Transcript_2/2_Confidence_0.250_Length_1867**
	**Locus_2424_Transcript_1/1_Confidence_0.000_Length_1734**
**CYP96**	**Locus_2163_Transcript_3/11_Confidence_0.346_Length_520**
	**Locus_3183_Transcript_3/23_Confidence_0.323_Length_874**
	**Locus_3183_Transcript_4/23_Confidence_0.387_Length_879**
	**Locus_3183_Transcript_7/23_Confidence_0.452_Length_1677**
	**Locus_3183_Transcript_8/23_Confidence_0.516_Length_1682**
	**Locus_3183_Transcript_6/23_Confidence_0.387_Length_1020**
	**Locus_3183_Transcript_5/23_Confidence_0.452_Length_1025**
	**Locus_3183_Transcript_17/23_Confidence_0.452_Length_1117**
	**Locus_3183_Transcript_19/23_Confidence_0.452_Length_1113**
	**Locus_3183_Transcript_9/23_Confidence_0.581_Length_1828**
	**Locus_3183_Transcript_13/23_Confidence_0.581_Length_1920**
	**Locus_3183_Transcript_16/23_Confidence_0.516_Length_1878**
	**Locus_3183_Transcript_15/23_Confidence_0.548_Length_1990**
	**Locus_3183_Transcript_20/23_Confidence_0.581_Length_2078**
	**Locus_3183_Transcript_21/23_Confidence_0.419_Length_1275**
	**Locus_3183_Transcript_22/23_Confidence_0.581_Length_2081**
	**Locus_3183_Transcript_23/23_Confidence_0.290_Length_1288**
	**Locus_11035_Transcript_1/1_Confidence_0.000_Length_1723**
	**Locus_21546_Transcript_1/1_Confidence_0.000_Length_1236**
	**Locus_30147_Transcript_1/1_Confidence_0.000_Length_300**
	**Locus_12542_Transcript_7/9_Confidence_0.333_Length_577**
**CYP97**	**Locus_6410_Transcript_12/23_Confidence_0.370_Length_2302**
	**Locus_6410_Transcript_7/23_Confidence_0.407_Length_2315**

	**Locus_6410_Transcript_10/23_Confidence_0.407_Length_2320**
	**Locus_6410_Transcript_13/23_Confidence_0.407_Length_2349**
	**Locus_6410_Transcript_14/23_Confidence_0.407_Length_2422**
	**Locus_6410_Transcript_15/23_Confidence_0.556_Length_4023**
	**Locus_6410_Transcript_16/23_Confidence_0.593_Length_4842**
	**Locus_6410_Transcript_18/23_Confidence_0.556_Length_4938**
	**Locus_6410_Transcript_3/23_Confidence_0.111_Length_928**
	**Locus_6410_Transcript_5/23_Confidence_0.111_Length_928**
	**Locus_6410_Transcript_4/23_Confidence_0.111_Length_933**
	**Locus_6410_Transcript_6/23_Confidence_0.222_Length_1366**
	**Locus_6410_Transcript_20/23_Confidence_0.593_Length_4735**
	**Locus_6410_Transcript_21/23_Confidence_0.556_Length_4876**
	**Locus_6410_Transcript_2/23_Confidence_0.148_Length_625**
	**Locus_9437_Transcript_4/8_Confidence_0.538_Length_2054**
	**Locus_9437_Transcript_8/8_Confidence_0.154_Length_2219**
	**Locus_9437_Transcript_7/8_Confidence_0.692_Length_2201**
	**Locus_9437_Transcript_2/8_Confidence_0.538_Length_2553**
	**Locus_9437_Transcript_1/8_Confidence_0.308_Length_1777**
	**Locus_5217_Transcript_4/6_Confidence_0.222_Length_823**
	**Locus_5217_Transcript_5/6_Confidence_0.222_Length_827**
	**Locus_5217_Transcript_1/6_Confidence_0.667_Length_1286**
	**Locus_5217_Transcript_2/6_Confidence_0.667_Length_1290**
	**Locus_5217_Transcript_6/6_Confidence_0.111_Length_1187**
	**Locus_5217_Transcript_3/6_Confidence_0.444_Length_1361**
	**Locus_15204_Transcript_2/2_Confidence_0.400_Length_995**
	**Locus_15204_Transcript_1/2_Confidence_0.800_Length_757**
	**Locus_6410_Transcript_22/23_Confidence_0.370_Length_4847**
**CYP98**	**Locus_5966_Transcript_4/8_Confidence_0.333_Length_881**
	**Locus_5966_Transcript_3/8_Confidence_0.389_Length_884**
	**Locus_5966_Transcript_5/8_Confidence_0.389_Length_1106**

	Locus_5966_Transcript_2/8_Confidence_0.389_Length_1805
	Locus_5966_Transcript_1/8_Confidence_0.389_Length_1808
	Locus_5966_Transcript_6/8_Confidence_0.389_Length_1808
	Locus_5966_Transcript_8/8_Confidence_0.056_Length_1768
	Locus_5966_Transcript_7/8_Confidence_0.111_Length_1065
	Locus_19973_Transcript_1/1_Confidence_0.000_Length_1735
CYP702	Locus_22762_Transcript_3/5_Confidence_0.143_Length_696
	Locus_22762_Transcript_5/5_Confidence_0.000_Length_1642
	Locus_22762_Transcript_2/5_Confidence_0.571_Length_1702
	Locus_11181_Transcript_8/17_Confidence_0.217_Length_376
	Locus_11181_Transcript_9/17_Confidence_0.217_Length_363
CYP704	Locus_21790_Transcript_1/3_Confidence_0.750_Length_416
	Locus_21790_Transcript_2/3_Confidence_0.500_Length_509
CYP705	Locus_2163_Transcript_7/11_Confidence_0.538_Length_1351
	Locus_2163_Transcript_8/11_Confidence_0.538_Length_1693
	Locus_2163_Transcript_11/11_Confidence_0.154_Length_678
	Locus_28508_Transcript_1/1_Confidence_0.000_Length_300
	Locus_3897_Transcript_2/3_Confidence_0.625_Length_792
	Locus_3897_Transcript_3/3_Confidence_0.500_Length_912
	Locus_3897_Transcript_1/3_Confidence_0.625_Length_922
	Locus_6868_Transcript_5/14_Confidence_0.200_Length_287
	Locus_5093_Transcript_4/4_Confidence_0.000_Length_680
	Locus_17870_Transcript_2/3_Confidence_0.200_Length_1007
CYP706	Locus_9265_Transcript_3/3_Confidence_0.167_Length_360
	Locus_9265_Transcript_2/3_Confidence_0.500_Length_466
	Locus_9265_Transcript_1/3_Confidence_0.333_Length_466
	Locus_22720_Transcript_1/1_Confidence_0.000_Length_304
	Locus_1182_Transcript_2/17_Confidence_0.516_Length_1813
	Locus_1182_Transcript_3/17_Confidence_0.484_Length_1813
	Locus_1182_Transcript_1/17_Confidence_0.516_Length_1817

	Locus_1182_Transcript_4/17_Confidence_0.516_Length_1813
	Locus_1182_Transcript_11/17_Confidence_0.452_Length_1872
	Locus_1182_Transcript_8/17_Confidence_0.548_Length_1872
	Locus_1182_Transcript_13/17_Confidence_0.516_Length_1872
	Locus_1182_Transcript_12/17_Confidence_0.516_Length_1909
	Locus_1182_Transcript_14/17_Confidence_0.484_Length_1909
	Locus_1182_Transcript_7/17_Confidence_0.484_Length_1870
	Locus_1182_Transcript_10/17_Confidence_0.484_Length_1852
	Locus_1182_Transcript_6/17_Confidence_0.581_Length_1909
	Locus_1182_Transcript_15/17_Confidence_0.484_Length_1929
	Locus_1182_Transcript_5/17_Confidence_0.581_Length_1969
	Locus_1182_Transcript_9/17_Confidence_0.613_Length_2028
	Locus_18372_Transcript_1/1_Confidence_0.000_Length_284
	Locus_14353_Transcript_1/1_Confidence_0.000_Length_1114
	Locus_15761_Transcript_1/1_Confidence_0.000_Length_1304
	Locus_1182_Transcript_16/17_Confidence_0.226_Length_866
	Locus_12215_Transcript_6/8_Confidence_0.500_Length_2092
	Locus_12215_Transcript_7/8_Confidence_0.250_Length_2079
	Locus_12215_Transcript_8/8_Confidence_0.083_Length_1862
	Locus_28066_Transcript_1/1_Confidence_0.000_Length_559
CYP707	Locus_1174_Transcript_1/1_Confidence_0.000_Length_1127
	Locus_12634_Transcript_1/1_Confidence_0.000_Length_1613
CYP708	Locus_11181_Transcript_17/17_Confidence_0.000_Length_513
	Locus_5093_Transcript_1/4_Confidence_0.600_Length_457
CYP709	Locus_10475_Transcript_4/11_Confidence_0.312_Length_909
	Locus_10475_Transcript_8/11_Confidence_0.438_Length_2605
CYP712	Locus_6868_Transcript_4/14_Confidence_0.400_Length_671
	Locus_16293_Transcript_1/1_Confidence_0.000_Length_1727
CYP714	Locus_10475_Transcript_6/11_Confidence_0.438_Length_2115
CYP716	Locus_6034_Transcript_2/15_Confidence_0.120_Length_502

	**Locus_25282_Transcript_1/1_Confidence_0.000_Length_1460**
	**Locus_7923_Transcript_2/4_Confidence_0.333_Length_1584**
	**Locus_7923_Transcript_1/4_Confidence_0.444_Length_1589**
	**Locus_7923_Transcript_3/4_Confidence_0.667_Length_1593**
	**Locus_7923_Transcript_4/4_Confidence_0.333_Length_1655**
	**Locus_22920_Transcript_1/1_Confidence_0.000_Length_236**
	**Locus_28257_Transcript_1/1_Confidence_0.000_Length_364**
	**Locus_24538_Transcript_1/1_Confidence_0.000_Length_786**
	**Locus_18277_Transcript_1/1_Confidence_0.000_Length_293**
	**Locus_6034_Transcript_9/15_Confidence_0.200_Length_706**
	**Locus_6034_Transcript_1/15_Confidence_0.360_Length_1151**
	**Locus_6034_Transcript_3/15_Confidence_0.320_Length_1151**
	**Locus_6034_Transcript_13/15_Confidence_0.360_Length_1496**
	**Locus_6034_Transcript_5/15_Confidence_0.400_Length_1811**
	**Locus_6034_Transcript_15/15_Confidence_0.040_Length_831**
**CYP721**	**Locus_10475_Transcript_1/11_Confidence_0.250_Length_749**
	**Locus_10475_Transcript_11/11_Confidence_0.000_Length_1062**
**CYP722**	**Locus_6868_Transcript_9/14_Confidence_0.467_Length_1899**
**CYP734**	**Locus_2860_Transcript_2/16_Confidence_0.227_Length_535**
	**Locus_3941_Transcript_5/6_Confidence_0.222_Length_1272**
	**Locus_3941_Transcript_1/6_Confidence_0.667_Length_1619**
	**Locus_3941_Transcript_3/6_Confidence_0.556_Length_1774**
	**Locus_3941_Transcript_2/6_Confidence_0.444_Length_669**
	**Locus_3941_Transcript_4/6_Confidence_0.111_Length_1852**
	**Locus_3941_Transcript_3/6_Confidence_0.556_Length_1774**
	**Locus_3941_Transcript_2/6_Confidence_0.444_Length_669**
**CYP735**	**Locus_20269_Transcript_1/1_Confidence_0.000_Length_720**
	**Locus_3780_Transcript_9/13_Confidence_0.462_Length_1358**
	**Locus_3780_Transcript_7/13_Confidence_0.500_Length_1389**
	**Locus_3780_Transcript_2/13_Confidence_0.462_Length_1291**

	**Locus_3780_Transcript_8/13_Confidence_0.577_Length_1997**
	**Locus_3780_Transcript_1/13_Confidence_0.269_Length_1024**
	**Locus_3780_Transcript_10/13_Confidence_0.115_Length_1666**
	**Locus_3780_Transcript_5/13_Confidence_0.231_Length_879**
	**Locus_3780_Transcript_4/13_Confidence_0.231_Length_878**
	**Locus_3780_Transcript_3/13_Confidence_0.269_Length_1519**
	**Locus_3780_Transcript_6/13_Confidence_0.269_Length_1568**

Table S6b: Putative nomenclature of identified *M. koenigii* full length genes encoding Cytochrome P450 using different public databases

**Transcript ID**	**Nelson database/ http://blast.uthsc.edu /blast.cgi**	**Bio catnet CYPED v6.0/ https://cyped.biocatnet.de/ workbench/blast**	**NCBI**	**TAIR**
**Locus_7540_Transcript_1/1_Confidence_0.000_Length_1583**	CYP51G1/CYP51A2	Cyp51/PREDICTED: sterol 14-demethylase-like	PREDICTED: obtusifoliol 14-alpha demethylase [*Citrus sinensis*]	putative obtusifoliol 14-alpha demethylase involved in sterol biosynthesis
**Locus_2312_Transcript_4/9_Confidence_0.417_Length_2270**	CYP71B34	PREDICTED: cytochrome P450 71B37-like|cytochrome P450	PREDICTED: cytochrome P450 71B34-like [*Citrus sinensis*]	CYP71B2/secondary metabolite biosynthetic process
**Locus_2312_Transcript_3/9_Confidence_0.458_Length_2289**	CYP71B34	PREDICTED: cytochrome P450 71B37-like|cytochrome P450	PREDICTED: cytochrome P450 71B34-like [*Citrus sinensis*]	CYP71B2/secondary metabolite biosynthetic process
**Locus_2312_Transcript_1/9_Confidence_0.458_Length_2300**	CYP71B34	PREDICTED: cytochrome P450 71B37-like|cytochrome P450	PREDICTED: cytochrome P450 71B34-like [*Citrus sinensis*]	CYP71B2/secondary metabolite biosynthetic process

**Locus_11455_Transcript_1/1_Confidence_0.000_Length_1818**	CYP71B35	hypothetical protein	PREDICTED: cytochrome P450 71A1-like [*Citrus sinensis*]	CYP71B22
**Locus_9283_Transcript_1/2_Confidence_0.333_Length_1824**	CYP721	PREDICTED: cytochrome P450 734A1-like	PREDICTED: cytochrome P450 734A1-like [*Citrus sinensis*]	CYP721A1
**Locus_2093_Transcript_4/5_Confidence_0.333_Length_2108**	CYP74B2	hydroperoxide lyase [*Citrus jambhiri*]	fatty acid hydroperoxide lyase [*Citrus sinensis*]	CYP74B2, HPL1, HYDROPEROXIDE LYASE 1/sterol metabolic process
**Locus_11826_Transcript_1/4_Confidence_0.714_Length_2049**	CYP74A	CYP74A	allene oxide synthase [*Citrus sinensis*]	ALLENE OXIDE SYNTHASE, AOS, CYP74A, CYTOCHROME P450 74A, DDE2, DELAYED DEHISCENCE 2/defense response, defense response to fungus, epoxygenase P450 pathway, jasmonic acid biosynthetic process, oxidation-reduction process, oxylipin biosynthetic process, oxylipin metabolic process, response to fungus, response to jasmonic acid, response to wounding, sterol metabolic process
**Locus_11826_Transcript_4/4_Confidence_0.143_Length_2049**	CYP74A	CYP74A	allene oxide synthase [*Citrus sinensis*]	ALLENE OXIDE SYNTHASE, AOS, CYP74A, CYTOCHROME P450 74A, DDE2, DELAYED DEHISCENCE 2/defense response, defense response to fungus, epoxygenase P450 pathway, jasmonic acid biosynthetic process, oxidation-reduction process, oxylipin biosynthetic process, oxylipin metabolic process, response to fungus, response to jasmonic acid, response to wounding, sterol metabolic process

**Locus_11826_Transcript_2/4_Confidence_0.714_Length_2061**	CYP74A	CYP74A	allene oxide synthase [*Citrus sinensis*]	ALLENE OXIDE SYNTHASE, AOS, CYP74A, CYTOCHROME P450 74A, DDE2, DELAYED DEHISCENCE 2/defense response, defense response to fungus, epoxygenase P450 pathway, jasmonic acid biosynthetic process, oxidation-reduction process, oxylipin biosynthetic process, oxylipin metabolic process, response to fungus, response to jasmonic acid, response to wounding, sterol metabolic process
**Locus_11826_Transcript_3/4_Confidence_0.429_Length_1919**	CYP74A	CYP74A	allene oxide synthase [*Citrus sinensis*]	ALLENE OXIDE SYNTHASE, AOS, CYP74A, CYTOCHROME P450 74A, DDE2, DELAYED DEHISCENCE 2/defense response, defense response to fungus, epoxygenase P450 pathway, jasmonic acid biosynthetic process, oxidation-reduction process, oxylipin biosynthetic process, oxylipin metabolic process, response to fungus, response to jasmonic acid, response to wounding, sterol metabolic process
**Locus_16409_Transcript_3/4_Confidence_0.500_Length_1727**	CYP75B1	cytochrome P450 71A1-like	PREDICTED: cytochrome P450 71A1-like [*Citrus sinensis*]	CYP81H1/defense response to other organism, indole glucosinolate metabolic process, oxidation-reduction process, secondary metabolite biosynthetic process OR CYP71B16/secondary metabolite biosynthetic process
**Locus_16409_Transcript_1/4_Confidence_0.750_Length_1728**	CYP75B1	cytochrome P450 71A1-like	PREDICTED: cytochrome P450 71A1-like [*Citrus sinensis*]	CYP81H1/defense response to other organism, indole glucosinolate metabolic process, oxidation-reduction process, secondary metabolite biosynthetic process OR CYP71B16/secondary metabolite biosynthetic process

**Locus_16409_Transcript_4/4_Confidence_0.000_Length_1652**	CYP75B1	cytochrome P450 71A1-like	PREDICTED: cytochrome P450 71A1-like [*Citrus sinensis*]	CYP81H1/defense response to other organism, indole glucosinolate metabolic process, oxidation-reduction process, secondary metabolite biosynthetic process OR CYP71B16/secondary metabolite biosynthetic process
**Locus_16409_Transcript_2/4_Confidence_0.250_Length_1653**	CYP75B1	cytochrome P450 71A1-like	PREDICTED: cytochrome P450 71A1-like [*Citrus sinensis*]	CYP81H1/defense response to other organism, indole glucosinolate metabolic process, oxidation-reduction process, secondary metabolite biosynthetic process OR CYP71B16/secondary metabolite biosynthetic process
**Locus_11745_Transcript_2/2_Confidence_0.000_Length_1803**	CYP75B1	PREDICTED: flavonoid 3-monooxygenase-like	PREDICTED: flavonoid 3'-monooxygenase [*Citrus sinensis*]	CYP75B1, CYTOCHROME P450 75B1, D501, TRANSPARENT TESTA 7, TT7/flavonoid biosynthetic process, oxidation-reduction process, response to UV, response to auxin, secondary metabolite biosynthetic process
**Locus_13518_Transcript_1/2_Confidence_0.750_Length_1779**	CYP76C4	CYP76B	PREDICTED: geraniol 8-hydroxylase-like isoform X3 [*Citrus sinensis*]	CYP71B20/defense response to other organism, oxidation-reduction process, secondary metabolite biosynthetic process
**Locus_10475_Transcript_5/11_Confidence_0.500_Length_2157**	CYP78A9	cytochrome P450 78A9-like isoform	PREDICTED: cytochrome P450 3A31-like isoform X1 [*Citrus sinensis*]	CCT2, PHOSPHORYLCHOLINE CYTIDYLYLTRANSFERASE2
**Locus_5093_Transcript_3/4_Confidence_0.400_Length_2576**	CYP79B3	NADPH--cytochrome P450 reductase	PREDICTED: NADPH--cytochrome P450 reductase isoform X1 [*Citrus sinensis*]	P450 REDUCTASE 2/phenylpropanoid general pathway
**Locus_5093_Transcript_2/4_Confidence_0.600_Length_2353**	CYP79B3	NADPH--cytochrome P450 reductase	PREDICTED: NADPH--cytochrome P450 reductase isoform X1 [*Citrus sinensis*]	P450 REDUCTASE 2/phenylpropanoid general pathway

**Locus_2612_Transcript_3/4_Confidence_0.333_Length_1908**	CYP81K2	CYP81K	PREDICTED: cytochrome P450 81D11-like [*Citrus sinensis*]	CYP81K2/defense response to other organism, indole glucosinolate metabolic process,oxidation-reduction process, secondary metabolite biosynthetic process
**Locus_1752_Transcript_1/7_Confidence_0.111_Length_1903**	CYP81D8	cytochrome P450 81D1-like	PREDICTED: isoflavone 3'-hydroxylase-like [*Citrus sinensis*]	CYP81D5/defense response to other organism, indole glucosinolate metabolic process, oxidation-reduction process, secondary metabolite biosynthetic process
**Locus_1752_Transcript_2/7_Confidence_0.111_Length_2104**	CYP81D8	cytochrome P450 81D1-like	PREDICTED: isoflavone 3'-hydroxylase-like [*Citrus sinensis*]	CYP81D5/defense response to other organism, indole glucosinolate metabolic process, oxidation-reduction process, secondary metabolite biosynthetic process
**Locus_1752_Transcript_6/7_Confidence_0.333_Length_1763**	CYP81D8	CYP81E	PREDICTED: isoflavone 2'-hydroxylase-like isoform X1 [*Citrus sinensis*]	CYP81D5/defense response to other organism, indole glucosinolate metabolic process, oxidation-reduction process, secondary metabolite biosynthetic process
**Locus_1752_Transcript_5/7_Confidence_0.444_Length_1767**	CYP81D8	isoflavone 2'-hydroxylase-like isoform X1/CYP81E	PREDICTED: isoflavone 2'-hydroxylase-like isoform X1 [*Citrus sinensis*]	CYP81D5/defense response to other organism, indole glucosinolate metabolic process, oxidation-reduction process, secondary metabolite biosynthetic process
**Locus_2616_Transcript_4/5_Confidence_0.375_Length_1917**	CYP82C4	CYP82G	PREDICTED: cytochrome P450 82C4-like [*Ziziphus jujuba*]	CYP82C4/cellular response to iron ion, oxidation-reduction process, secondary metabolite biosynthetic process
**Locus_2616_Transcript_1/5_Confidence_0.625_Length_1907**	CYP82C4	CYP82G	PREDICTED: cytochrome P450 82C4-like *[Ziziphus jujuba*]	CYP82C4/cellular response to iron ion, oxidation-reduction process, secondary metabolite biosynthetic process

**Locus_2616_Transcript_3/5_Confidence_0.250_Length_1851**	CYP82C4	CYP82G	PREDICTED: cytochrome P450 82C4-like [*Ziziphus jujuba*]	CYP82C4/cellular response to iron ion, oxidation-reduction process, secondary metabolite biosynthetic process
**Locus_2616_Transcript_2/5_Confidence_0.375_Length_1915**	CYP82C4	CYP82G	PREDICTED: cytochrome P450 82C4-like [*Ziziphus jujuba*]	CYP82C4/cellular response to iron ion, oxidation-reduction process, secondary metabolite biosynthetic process
**Locus_2096_Transcript_1/9_Confidence_0.182_Length_1978**	CYP82C4	cytochrome P450 82C4-like	PREDICTED: cytochrome P450 CYP82D47-like [*Citrus sinensis*]	CYP82C3/oxidation-reduction process, secondary metabolite biosynthetic process
**Locus_2096_Transcript_2/9_Confidence_0.091_Length_1980**	CYP82C4	cytochrome P450 82C4-like	PREDICTED: cytochrome P450 CYP82D47-like [*Citrus sinensis*]	CYP82C3/oxidation-reduction process, secondary metabolite biosynthetic process
**Locus_2006_Transcript_25/27_Confidence_0.321_Length_1534**	CYP82C4/C2/C3	cytochrome P450 82C4-like	cytochrome P450 82A3-like isoform X2 [*Citrus sinensis*]	CYP71B37
**Locus_3026_Transcript_71/74_Confidence_0.050_Length_1496**	CYP83A2	CYP83B	PREDICTED: cytochrome P450 83B1-like [*Citrus sinensis*]	CYP71B5/ cellular response to ethylene stimulus, cellular response to iron ion, cellular response to nitric oxide, oxidation-reduction process, secondary metabolite biosynthetic process
**Locus_3026_Transcript_67/74_Confidence_0.135_Length_2218**	CYP83A2	CYP83B	PREDICTED: cytochrome P450 83B1-like [*Citrus sinensis*]	CYP71B5/ cellular response to ethylene stimulus, cellular response to iron ion, cellular response to nitric oxide, oxidation-reduction process, secondary metabolite biosynthetic process
**Locus_3026_Transcript_62/74_Confidence_0.135_Length_2050**	CYP83A2	CYP83B	PREDICTED: cytochrome P450 83B1-like [*Citrus sinensis*]	CYP71B5/ cellular response to ethylene stimulus, cellular response to iron ion, cellular response to nitric oxide, oxidation-reduction process, secondary metabolite biosynthetic

				process
**Locus_19681_Transcript_1/1_Confidence_0.000_Length_1840**	CYP84A1	CYP84A	PREDICTED: cytochrome P450 84A1 [*Citrus sinensis*]	CYP84A1, CYTOCHROME P450 84A1, FAH1, FERULIC ACID 5-HYDROXYLASE 1/lignin biosynthetic process, oxidation-reduction process, phenylpropanoid biosynthetic process, response to UV-B
**Locus_10238_Transcript_1/1_Confidence_0.333_Length_1883**	CYP89A5	PREDICTED: cytochrome P450 89A2-like	PREDICTED: cytochrome P450 89A2-like [*Citrus sinensis*]	CYP89A3/ oxidation-reduction process, secondary metabolite biosynthetic process
**Locus_7472_Transcript_1/3_Confidence_0.400_Length_2057**	CYP90C1	3-epi-6-deoxocathasterone 23-monooxygenase-like isoform X1	PREDICTED: 3-epi-6-deoxocathasterone 23-monooxygenase isoform X1 [*Citrus sinensis*]	CYP90B1, DWARF 4, DWF4, PARTIALLY SUPPRESSING COI1 INSENSITIVITY TO JA 1, PSC1, SAV1, SHADE AVOIDANCE 1, SNP2, SUPPRESSOR OF NPH4 2/ brassinosteroid biosynthetic process, brassinosteroid homeostasis, jasmonic acid mediated signaling pathway, leaf development, leaf shaping, oxidation-reduction process, response to brassinosteroid, response to jasmonic acid, sterol metabolic process, unidimensional cell growth

**Locus_9617_Transcript_5/6_Confidence_0.571_Length_1910**	CYP90D1	CYP90D	PREDICTED: 3-epi-6-deoxocathasterone 23-monooxygenase [*Citrus sinensis*]	CYP90D1/Encodes a cytochrome P-450 gene that is involved in brassinosteroid biosynthesis, most likely in the conversion step of teasterone (TE) to 3-dehydroteasterone (3DT), and/or 6-deoxoteasterone (6-deoxoTE) to 6-deoxo-3-dehydroteasterone (6-deoxo3DT); or the conversion of cathasterone (CT) to TE, and/or 6-deoxocathasterone (6-deoxoCT) to 6-deoxoTE. Recently, CYP90D1 was shown to catalyse the C-23 hydroxylation of several brassinosteroids (the enzyme has a broad specificity for 22-hydroxylated substrates). Member of the CYP90C CYP450 family. Similar to Cytochrome P450 90C1 (ROT3).
**Locus_9617_Transcript_4/6_Confidence_0.571_Length_1919**	CYP90D1	CYP90D	PREDICTED: 3-epi-6-deoxocathasterone 23-monooxygenase [*Citrus sinensis*]	CYP90D1/Encodes a cytochrome P-450 gene that is involved in brassinosteroid biosynthesis, most likely in the conversion step of teasterone (TE) to 3-dehydroteasterone (3DT), and/or 6-deoxoteasterone (6-deoxoTE) to 6-deoxo-3-dehydroteasterone (6-deoxo3DT); or the conversion of cathasterone (CT) to TE, and/or 6-deoxocathasterone (6-deoxoCT) to 6-deoxoTE. Recently, CYP90D1 was shown to catalyse the C-23 hydroxylation of several brassinosteroids (the enzyme has a broad specificity for 22-hydroxylated substrates). Member of the CYP90C CYP450 family. Similar to Cytochrome P450 90C1 (ROT3).

**Locus_9617_Transcript_3/6_Confidence_0.571_Length_2005**	CYP90D1	CYP90D	PREDICTED: 3-epi-6-deoxocathasterone 23-monooxygenase [*Citrus sinensis*]	CYP90D1/Encodes a cytochrome P-450 gene that is involved in brassinosteroid biosynthesis, most likely in the conversion step of teasterone (TE) to 3-dehydroteasterone (3DT), and/or 6-deoxoteasterone (6-deoxoTE) to 6-deoxo-3-dehydroteasterone (6-deoxo3DT); or the conversion of cathasterone (CT) to TE, and/or 6-deoxocathasterone (6-deoxoCT) to 6-deoxoTE. Recently, CYP90D1 was shown to catalyse the C-23 hydroxylation of several brassinosteroids (the enzyme has a broad specificity for 22-hydroxylated substrates). Member of the CYP90C CYP450 family. Similar to Cytochrome P450 90C1 (ROT3).
**Locus_9617_Transcript_1/6_Confidence_0.714_Length_2014**	CYP90D1	CYP90D	PREDICTED: 3-epi-6-deoxocathasterone 23-monooxygenase [*Citrus sinensis*]	CYP90D1/Encodes a cytochrome P-450 gene that is involved in brassinosteroid biosynthesis, most likely in the conversion step of teasterone (TE) to 3-dehydroteasterone (3DT), and/or 6-deoxoteasterone (6-deoxoTE) to 6-deoxo-3-dehydroteasterone (6-deoxo3DT); or the conversion of cathasterone (CT) to TE, and/or 6-deoxocathasterone (6-deoxoCT) to 6-deoxoTE. Recently, CYP90D1 was shown to catalyse the C-23 hydroxylation of several brassinosteroids (the enzyme has a broad specificity for 22-hydroxylated substrates). Member of the CYP90C CYP450 family. Similar to Cytochrome P450 90C1 (ROT3).
**Locus_2871_Transcript_1/2_Confidence_0.750_Length_1863**	CYP94D2	PREDICTED: cytochrome P450 94A1-like	PREDICTED: cytochrome P450 94A1-like [*Citrus sinensis*]	CYP94B1/CYP86C1

**Locus_2871_Transcript_2/2_Confidence_0.250_Length_1867**	CYP94D2	PREDICTED: cytochrome P450 94A1-like	PREDICTED: cytochrome P450 94A1-like [*Citrus sinensis*]	CYP94B1/CYP86C1
**Locus_2424_Transcript_1/1_Confidence_0.000_Length_1734**	CYP94D2	PREDICTED: cytochrome P450 94A1-like	PREDICTED: cytochrome P450 94A1-like [*Citrus sinensis*]	CYP94B1/CYP86C1
**Locus_3183_Transcript_7/23_Confidence_0.452_Length_1677**	CYP96A10	-	PREDICTED: peptidyl-prolyl cis-trans isomerase CYP37, chloroplastic isoform X1 [*Citrus sinensis*]	Cyclophilin-like peptidyl-prolyl cis-trans isomerase family protein
**Locus_11035_Transcript_1/1_Confidence_0.000_Length_1723**	CYP96A10	CYP86B	PREDICTED: alkane hydroxylase MAH1 [*Vitis vinifera*]	CYP96A9/CYP96A8
**Locus_6410_Transcript_15/23_Confidence_0.556_Length_4023**	CYP97A3	CYP97B	cytochrome P450 [*Citrus unshiu*]	CYP97A3, LUT5, LUTEIN DEFICIENT 5/carotenoid biosynthetic process, oxidation-reduction process, xanthophyll biosynthetic process
**Locus_6410_Transcript_16/23_Confidence_0.593_Length_4842**	CYP97A3	CYP97B	cytochrome P450 [*Citrus unshiu*]	CYP97A3, LUT5, LUTEIN DEFICIENT 5/carotenoid biosynthetic process, oxidation-reduction process, xanthophyll biosynthetic process
**Locus_6410_Transcript_20/23_Confidence_0.593_Length_4735**	CYP97A3	CYP97B	cytochrome P450 [*Citrus unshiu*]	CYP97A3, LUT5, LUTEIN DEFICIENT 5/carotenoid biosynthetic process, oxidation-reduction process, xanthophyll biosynthetic process
**Locus_6410_Transcript_21/23_Confidence_0.556_Length_4876**	CYP97A3	CYP97B	cytochrome P450 [*Citrus unshiu*]	CYP97A3, LUT5, LUTEIN DEFICIENT 5/carotenoid biosynthetic process, oxidation-reduction process, xanthophyll biosynthetic process

**Locus_19973_Transcript_1/1_Confidence_0.000_Length_1735**	CYP98A3	PREDICTED: cytochrome P450 98A2-like	PREDICTED: cytochrome P450 98A2 [*Citrus sinensis*]/p-coumaryl-CoA 3'-hydroxylase [Populus alba x Populus grandidentata	CYP98A3/encodes coumarate 3-hydroxylase (C3H), a P450-dependent monooxygenase. coumarin biosynthetic process, flavonoid biosynthetic process, lignin biosynthetic process, oxidation-reduction process, phenylpropanoid biosynthetic process
**Locus_22762_Transcript_5/5_Confidence_0.000_Length_1642**	CYP702A6/A5/A1/A2	CYP702A6	PREDICTED: cytochrome P450 708A2-like [*Camelina sativa*]	CYP702A6/A5/A1/A2 involved in brassinosteroid biosynthetic process, brassinosteroid homeostasis, multicellular organism development, oxidation-reduction process, sterol metabolic process
**Locus_22762_Transcript_2/5_Confidence_0.571_Length_1702**	CYP702A6/A5/A1/A2	CYP702A6	PREDICTED: cytochrome P450 708A2-like [*Camelina sativa*]	CYP702A6/A5/A1/A2 involved in brassinosteroid biosynthetic process, brassinosteroid homeostasis, multicellular organism development, oxidation-reduction process, sterol metabolic process
**Locus_15761_Transcript_1/1_Confidence_0.000_Length_1304**	CYP706A4	-	PREDICTED: 8-hydroxygeraniol dehydrogenase-like [*Citrus sinensis*]	CAD6/CAD6
**Locus_12215_Transcript_6/8_Confidence_0.500_Length_2092**	CYP706A4	CYP706A	PREDICTED: geraniol 8-hydroxylase [*Ricinus communis*]	CYP706A4/oxidation-reduction process, secondary metabolite biosynthetic process
**Locus_12215_Transcript_7/8_Confidence_0.250_Length_2079**	CYP706A4	CYP706A	PREDICTED: geraniol 8-hydroxylase [*Ricinus communis*]	CYP706A4/oxidation-reduction process, secondary metabolite biosynthetic process
**Locus_12215_Transcript_8/8_Confidence_0.083_Length_1862**	CYP706A4	CYP706A	PREDICTED: geraniol 8-hydroxylase [*Ricinus communis*]	CYP706A4/oxidation-reduction process, secondary metabolite biosynthetic process

**Locus_12634_Transcript_1/1_Confidence_0.000_Length_1613**	CYP707A4	CYP707A/abscisic acid 8'-hydroxylase 3-like precursor	abscisic acid 8'-hydroxylase 3-like precursor [*Citrus sinensis*]	CYP707A4/ ABA 8'-hydroxylase activity, involved in abscisic acid catabolic process, brassinosteroid biosynthetic process, brassinosteroid homeostasis, multicellular organism development, oxidation-reduction process, sterol metabolic process
**Locus_10475_Transcript_8/11_Confidence_0.438_Length_2605**	CYP709B2	cytochrome P450 78A9-like isoform X3	PREDICTED: cytochrome P450 4C1-like isoform X3 [*Citrus sinensis*]	CCT2, PHOSPHORYLCHOLINE CYTIDYLYLTRANSFERASE2
**Locus_16293_Transcript_1/1_Confidence_0.000_Length_1727**	CYP712A1	PREDICTED: cytochrome P450 93A1-like	PREDICTED: 3,9-dihydroxypterocarpan 6A-monooxygenase [*Citrus sinensis*]	CYP712A1/ oxidation-reduction process, secondary metabolite biosynthetic process
**Locus_10475_Transcript_6/11_Confidence_0.438_Length_2115**	CYP714A1	cytochrome P450 78A9-like isoform X1	PREDICTED: cytochrome P450 3A31-like isoform X1 [*Citrus sinensis*]	CCT2, PHOSPHORYLCHOLINE CYTIDYLYLTRANSFERASE2
**Locus_3941_Transcript_3/6_Confidence_0.556_Length_1774**	CYP734A1 old name CYP72B1	PREDICTED: cytochrome P450 734A1-like	PREDICTED: cytochrome P450 CYP749A22-like [*Citrus sinensis*]	DUF2
**Locus_3941_Transcript_3/6_Confidence_0.556_Length_1774**	CYP734A1 old name CYP72B2	PREDICTED: cytochrome P450 734A1-like	PREDICTED: cytochrome P450 CYP749A22-like [*Citrus sinensis*]	DUF2

Table S7: Localization prediction of MkTPSs using different software tools

	ChlorpP	WoLF PSORT	Predotar	TargetP
**MkTPS1**	Chloroplast	Chloroplast	Plastid	Chloroplast
**MkTPS2**	none	cytosol	None	none
**MkTPS3**	Chloroplast	Chloroplast	possibly plastid	Chloroplast
**MkTPS4**	Chloroplast	Chloroplast	possibly plastid	Chloroplast
**MkTPS5**	none	cytosol	None	none
**MkTPS6**	none	cytosol/nucleus	None	none
**MkTPS7**	Chloroplast	cytosol/chloroplast	None	none

Table S8: Identity and similarity score matrix of MkTPS1 and MkTPS2 with characterized A) Sabinene synthases and B) α-Farnesene synthases

A	Other characterized Sabinene synthases	MkTPS1
1	RlemTPS2	I- 80%, S-87%
2	SoSS	I- 43%, S-62%
3	HcMTPS7	I- 41%, S-60%
4	PsSS	I- 31%, S-47%
5	TpSS	I- 29%, S-51%
B	Other characterized α-Farnesene synthases (AFS)	MkTPS2
1	VvAFS	I- 54%, S-71%
2	CmAFS	I- 46%, S-5%6
3	CsAFS/BOCS	I- 45%, S-64%
4	VvAFS/BOCS	I- 38%, S-56%
5	RcAFS	I- 35%, S-55%
6	OfTPS4	I- 36%, S-53%
7	AtAFS/BOCS	I- 35%, S-55%
8	MdAFS1/BOCS	I- 35%, S-54%
9	PaAFS	I- 33%, S-53%
10	PtAFS	I- 32%, S-51%
11	PgXeTPS/bocs	I- 32%, S-52%
12	AdAFS/bocs	I- 26%, S-47%

Table S9. List of primers used in the study

Name	Sequence (5'-3')	Use
MKTPS1 F1	GCATATGGCTACTACTAAGCCTGCC	pet32a(+) cloning forward primer
MKTPS1 R1	CGCGGCCGCTGGAATGGGATCAAAAAATAAAG	pet32a(+) cloning reverse primer
MKTPS1 F2	GTCTAGAATGGCTCTCAATCTGCTCTCTTC	GFP cloning forward vector
MKTPS1 R2	CGGATCCGGCATACGATTCTCCCTTATA	GFP cloning reverse primer
MKTPS1 F3	CGCACATTAGTGAACCAT	internal sequencing
MKTPS1 F4	CTTATGGACCGTGGGGGATATATTCG	internal sequencing
MKTPS1 R3	CCCCCACGGTCCATAAGAAATTCTC	internal sequencing
MKTPS2 F1	GCATATGTCTTTACAAGTTTCAGCCTC	pet32a(+) cloning forward primer
MKTPS2 R1	CGCGGCCGCTATCGGCACAGGATTAATAAGC	pet32a(+) cloning reverse primer
MKTPS2 F2	GTCTAGAATGTCTTTACAAGTTTCAGCCTC	GFP cloning forward vector
MKTPS2 R2	CGGATCCTGCATCAATCAATTCAAGTGCAT	GFP cloning reverse primer
MKTPS2 R3	CGGATCCTATCGGCACACCATTAATAAGC	internal sequencing
MKActin F	GTCAGGGACATGAAGGAAAAGC	Endogenous control for qPCR
MKActin R	TCTTGGAGGTCTCAAGCTCTTGT	Endogenous control for qPCR
MKF-BOX F	TCGCCACTGGGTTTGATGAT	Endogenous control for qPCR
MKF-BOX R	AACAAGGAACGCGGGAACTT	Endogenous control for qPCR
MKTPS1 RT F	TGGAGGATGCGAAGATTGG	TPS1 qPCR primer
MKTPS1 RTR	CATGTCCGGTCCACTTTCGT	TPS1 qPCR primer
MKTPS2 RTF	CCAACTGCTGTGCCAATGC	TPS2 qPCR primer
MKTPS2 RTR	GACATCTGCAACGCGTGTAAG	TPS2 qPCR primer

References:

1. Ito, C., Thoyama, Y., Omura, M., Kajiura, I. & Furukawa, H. Alkaloidal Constituents of *Murraya koenigii*.Isolation and structural elucidation of novel binary carbazolequinones and carbazole alkaloids. *Chem. Pharm. Bull.* **41**, 2096-2100 (1993).
2. Chowdhury, B. K., Jha, S., Bhattacharyya, P. & Mukherjee, J. Two new carbazole alkaloids from *Murraya koenigii. Ind. J. Chem.* **40B**, 490-494 (2001).
3. Adebajo, A. C. *et al.* Evaluation of the ethnomedical claims of *Murraya koenigii*. *Pharm. Biol.* **42**, 610-620 (2005).
4. Rahman, M. M. & Gray, A. I. A benzofuran derivative and carbazole alkaloids from *Murraya koenigii* and their antimicrobial activity. *Phytochemistry* **66**, 1601-1606 (2005).
5. Chakrabarty, M. *et al.* Carbazole alkaloids from *Murraya koenigii*. *Phytochemistry* **46**, 751-755 (1997)
6. Wang, Y. S., He, H. P., Shen, Y. M., Hong, X. & Hao, X. J. Two new carbazole alkaloids from *Murraya koenigii*. *J. Nat. Prod.* **66**, 416-418 (2003).
7. Kureel, S. P., Kapil, R. S. & Popli, S. P. Two novel alkaloids from *Murraya koenigii* spreng: mahanimbicine and bicyclomahanimbicine. *Chem. Ind.* **18**, 958 (1970).
8. Ganesan, P., Phaiphan, A., Murugan, Y. & Baharin, B.S. Comparative study of bioactive compounds in curry and coriander leaves: An update. *J. chem. pharm. res.* **5**, 590-594 (2013).

9. Naz, S., Saied, S., Ahmed, A. & Shahid, S. M. Three new carbazole alkaloids and biological activities of *Murraya koenigii*. *J. Asian Nat. Prod. Res.* **17**, 7-13 (2015).

10. Uvarani, C. *et al.* Axially chiral biscarbazoles and biological evaluation of the constituents from *Murraya koenigii*. *Fitoterapia*. **94**, 10-20 (2014).

11. Tachibana, Y., Kikuzaki, H., Lajis, N. H. & Nakatani, N. Antioxidative activity of carbazoles from *Murraya koenigii* leaves. *J. Agric. Food Chem.* **49**, 5589-5594 (2001).

12. Takeya, K., Itoigawa, M. & Furukawa, H. Triphasic inotropic response of guinea-pig papillary muscle to murrayaquinone-A isolated from Rutaceae. *Eur. J. Pharmacol.* **169**, 137-145 (1989).

13. Tantapakul, C., Phakhodee, W., Laphookhieo, S., Ritthiwigrom, T. & Cheenpracha, S. Cytotoxic carbazole alkaloids from the stems of *Murraya koenigii. Chem. Nat. Comp.* **50**, 186-188 (2014).

14. Reisch, J., Adebajo, A. C., Kumar, V. & Aladesanmi, A. J. Two carbazole alkaloids from *Murraya koenigii. Phytochemistry* **36**, 1073-1076 (1994).

15. Adebajo, A. C. *et al.* Anti-trichomonal, biochemical and toxicological activities of methanolic extract and some carbazole alkaloids isolated from the leaves of *Murraya koenigii* growing in Nigeria. *Phytomed.* **13**, 246-254 (2006).

16. Roy, M. K., Thalang, V. N., Trakoontivakorn, G. & Nakahara, K. Mechanism of mahenineinduced apoptosis in human leukemia cell (HL-60). *Biochem. Pharmacol.* **67**, 41-51 (2004).

17. Kok, Y. Y. *et al.* Anti-tumour promoting activity and antioxidant properties of girinimbine isolated from the stem bark of *Murraya koenigii* S. *Molecules*. **17**, 4651-4660 (2012).

18. Reisch, J., Goj, O., Wickramasinghe, A., Herath, H. M. T. B. & Henkel, G. Carbazole alkaloids from seeds of *Murraya koenigii*. *Phytochemistry* **31**, 2877-2879 (1992).

19. Ito, C. *et al*. Induction of apoptosis by carbazole alkaloids isolated from *Murraya koenigii*. *Phytomed*. **13b**, 359-365 (2006).

20. Rehman, F., Khan, M. F., Khan, I., Shareef, H. & Marwat, S. K. Analgesic Activity of Carbazole Alkaloid from *Murraya paniculata* Linn. (Rutaceae). *American-Eurasian J. Agric. & Environ. Sci*. **14**, 240-245 (2014).

21. Bhattacharya, L., Roy, S. K. & Chakrovorty, D. P. Structure of the carbazole alkaloid isomurrayazoline from *Murraya koenigii*. *Phytochemistry* **21**, 2432–2433 (1982).

22. Handral, H. K., Pandith, A. & Shruthi S. D. A Review on *Murraya Koenigii*: Multipotenet medicinal plant. *Asian J. Pharm. Clin. Res*. **5**, 5-14 (2012).

23. Nakamura, S. *et al*. Alkaloids from Sri Lankan curry-leaf (*Murraya koenigii*) display melanogenesis inhibitory activity: structures of karapinchamines A and B. *Bioorg. Med. Chem*. **21**, 1043-1049 (2013).

24. Rao, L. J. M., Ramalakshmi, K., Borse, B. B. & Raghavan, B. Antioxidant and radical-scavenging carbazole alkaloids from the oleoresin of curry leaf (*Murraya koenigii* Spreng.). *Food Chem*. **100**, 742–747 (2007).

25. Joshi, B. S., Kamat, V. N. & Gawad, D. H. On the structures of girinimbine, mahanimbine, isomahanimbine, koenimbidine and murrayacine. *Tetrahedron*. **26**, 1475-1482 (1970).

26. Patel, O. P. *et al*. Naturally Occurring Carbazole Alkaloids from *Murraya koenigii* as Potential Antidiabetic Agents. *J. Nat. Prod*. [Epub ahead of print] (2016)

27. Mandal, S. *et al*. Antidiarrhoeal activity of carbazole alkaloids from *Murraya koenigii* Spreng (Rutaceae) seeds. *Fitoterapia* **81**, 72–74 (2010).

28. Nalli, Y. *et al*. Four new carbazole alkaloids from *Murraya koenigii* that display anti-inflammatory and anti-microbial activities. *Org. Biomol. Chem*. **14**, 3322-3332 (2016).

29. Fiebig, M., Pezzuto, J. M., Soejarto, D. D. & Kinghorn, A. D. Koenoline, a further cytotoxic carbazole alkaloid from *Murraya koenigii. Phytochemistry* **24**, 3041-3043 (1985).

30. Nagappan, T., Segaran, T. C., Wahid, M. E., Ramasamy, P. & Vairappan, C. S. Efficacy of carbazole alkaloids, essential oil and extract of *Murraya koenigii* in enhancing subcutaneous wound healing in rats. *Molecules* **17**, 14449-14463 (2012).

31. Dineshkumar, B., Mitra, A. & Mahadevappa, M. Antidiabetic and hypolipidemic effects of mahanimbine (carbazole alkaloid) from *Murraya koenigii* (Rutaceae) leaves. *Int. J. Phytomed.* **2**, 22-30 (2010).

32. Bhattacharyya, P. & Chakraborty, A. Mukonal, a probable biogenetic intermediate of pyranocarbazole alkaloids from *Murraya koenigii. Phytochemistry* **23**, 471-472 (1984).

33. Sunthitikawinsakul, A. *et al*. Coumarins and carbazoles from *Clausena excavata* exhibited antimycobacterial and antifungal activities. *Planta Med*. **69**, 155-157 (2003).

34. Choudhury, B. K. & Chakraborty, D. P. Mukoeic acid, the first carbazole carboxylic acid from a plant source. *Phytochemistry* **10**, 1967-1970 (1971).

35. Ishikura, M., Abe, T., Choshi, T. & Hibino, S. Simple indole alkaloids and those with a non-rearranged monoterpenoid unit. *Nat. Prod. Rep.* **30**, 694-752 (2013).

36. Mukherjee, M. Mukherjee, S., Shaw, A. K. & Ganguly, S. N. Mukonicine, a carbazole alkaloid from leaves of *Murraya koenigii*. *Phytochemistry* (1983).

37. Chakraborty, D. P., Barman, B. K. & Bose, P. K. On the constitution of murrayanine, a carbazole derivative isolated from *Murraya koenigii* Spreng. *Tetrahedron* **21**, 681-685 (1965).

38. Priya, R. M., Blessed, B. P. & Nija, S. Chemical composition, antibacterial and antioxidant profile of essential oil from *Murraya koenigii* (l.) leaves. *Avicenna J. Phytomed.* **4**, 200-214 (2014).

39. Ramsewak, R. S., Nair, M. G., Strasburg, G. M., DeWitt, D. L. & Nitiss, J. L. Biologically active carbazole alkaloids from *Murraya koenigii*. *J. Agric. Food Chem.* **47**, 444-447 (1999).

40. Itoigawa, M. *et al.* Antitumor agents. Carbazole alkaloid murrayaquinone A and related synthetic carbazolequinones as cytotoxic agents. *J. Nat. Prod.* **63**, 893-897 (2000).

41. Ahmad, K., Tan, S. P., Hazni, H. & Nafiah, M. A. Cyclic monoterpenoid pyranocarbazole alkaloids from the bark of *Murraya koenigii* (L.) Spreng. *Jurnal Teknologi.* **77**, 73–77 (2015).

42. Adebajo, A. C. & Reisch, J. Minor furocoumarins of *Murraya koenigii*. *Fitoterpia* **71**, 334-337 (2000).

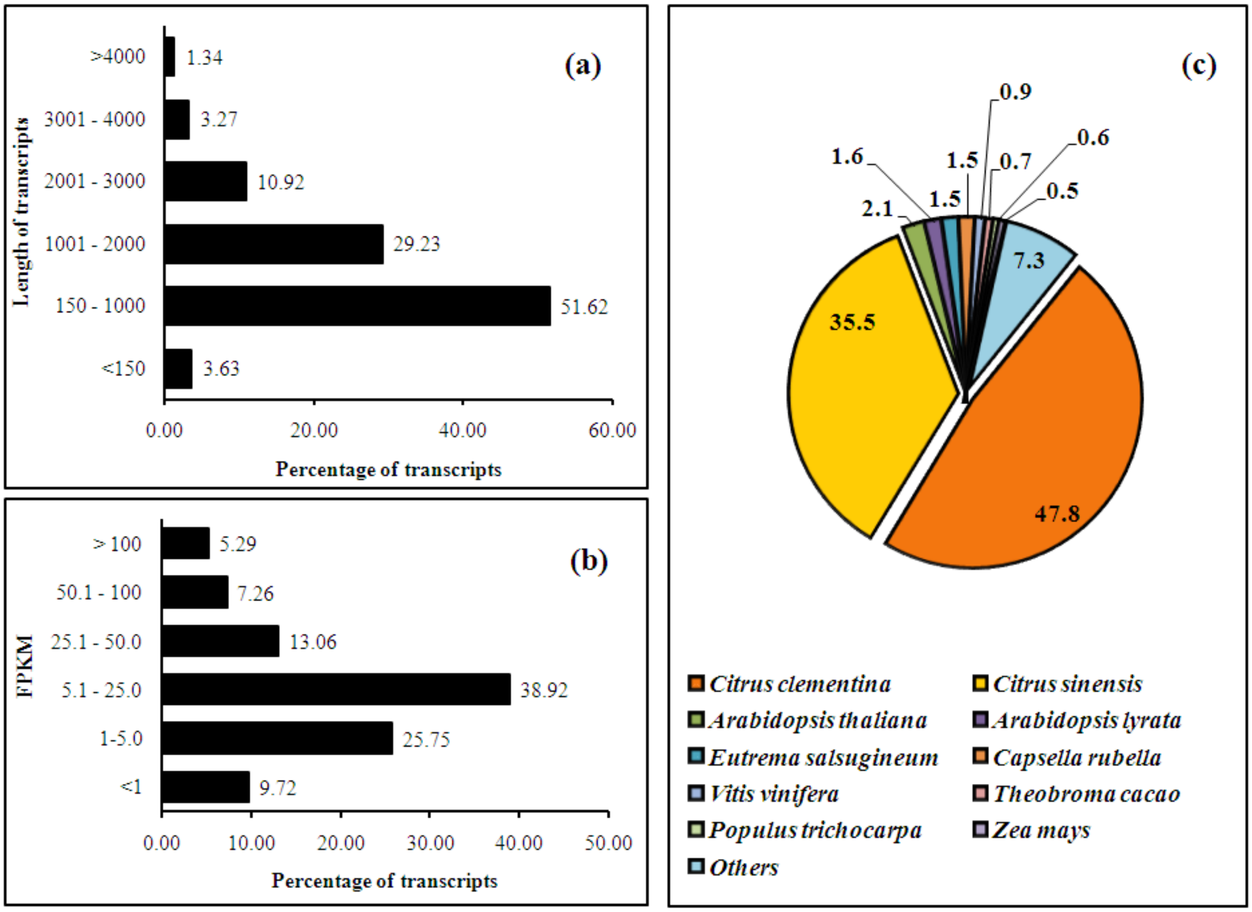

Figure S1. a) Length distribution of total assembled transcripts in *M. koenigii*, b) FPKM distribution of assembled transcripts with length ≥ 150 bp, c) Organismal distribution of *M. koenigii* transcripts annotated according to NCBI BLASTX top hits.

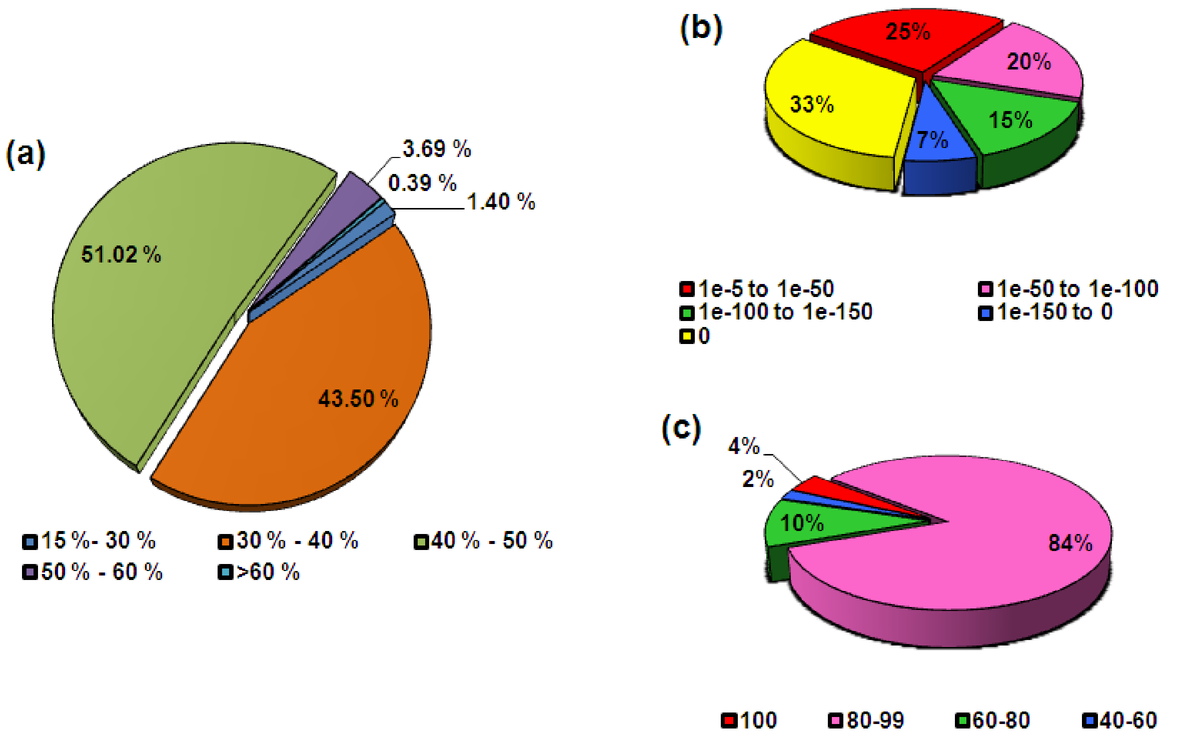

Figure S2. a) GC % distribution of assembled transcripts with length ≥150bp, b) E-value distribution of assembled transcripts with length ≥150bp and FPKM ≥1, balstx against NCBI nr database and (c) Similarity score distribution of blastx results against NCBI nr database

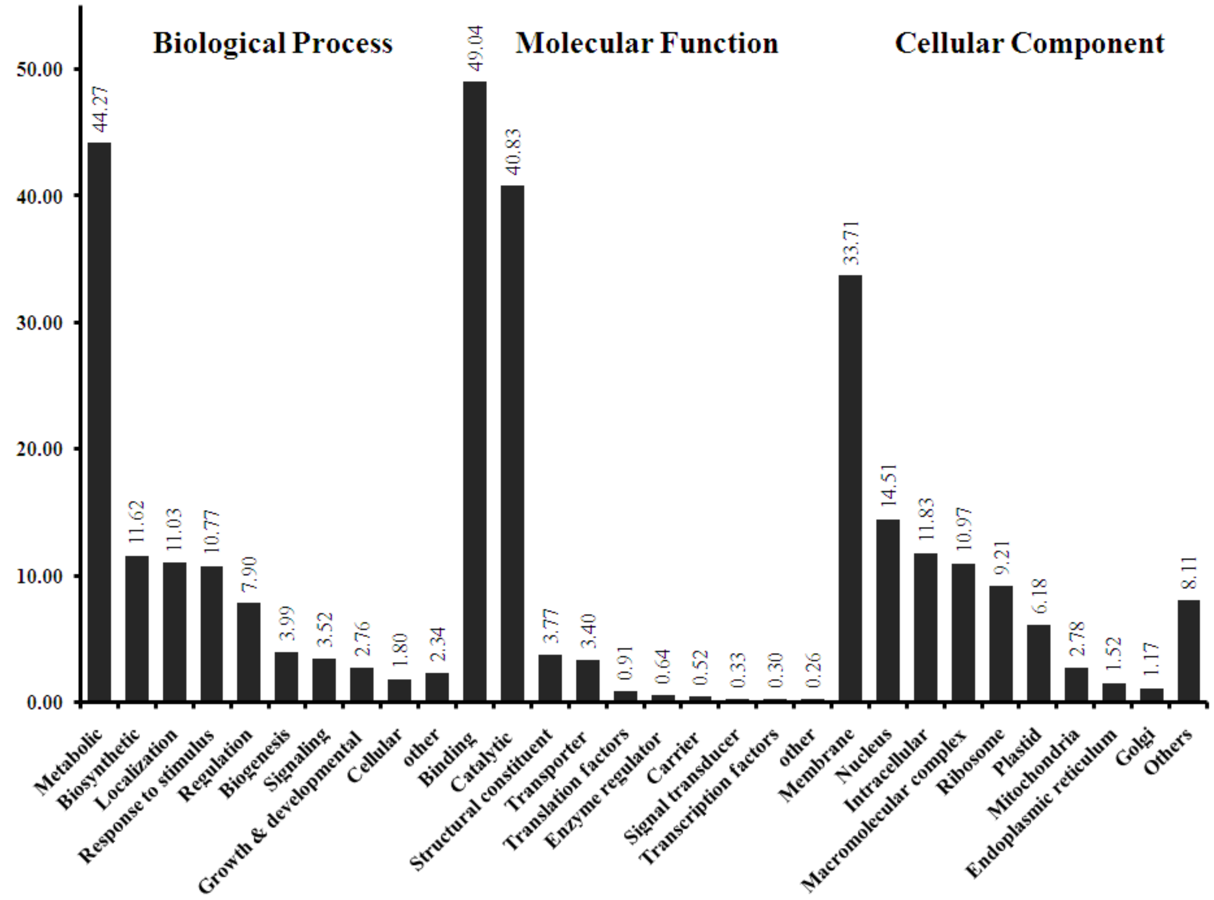

Figure S3. Gene ontology classification as derived from Uniprot annotation: Gene ontology has 3 main categories including Biological processes, Molecular function, Cellular component

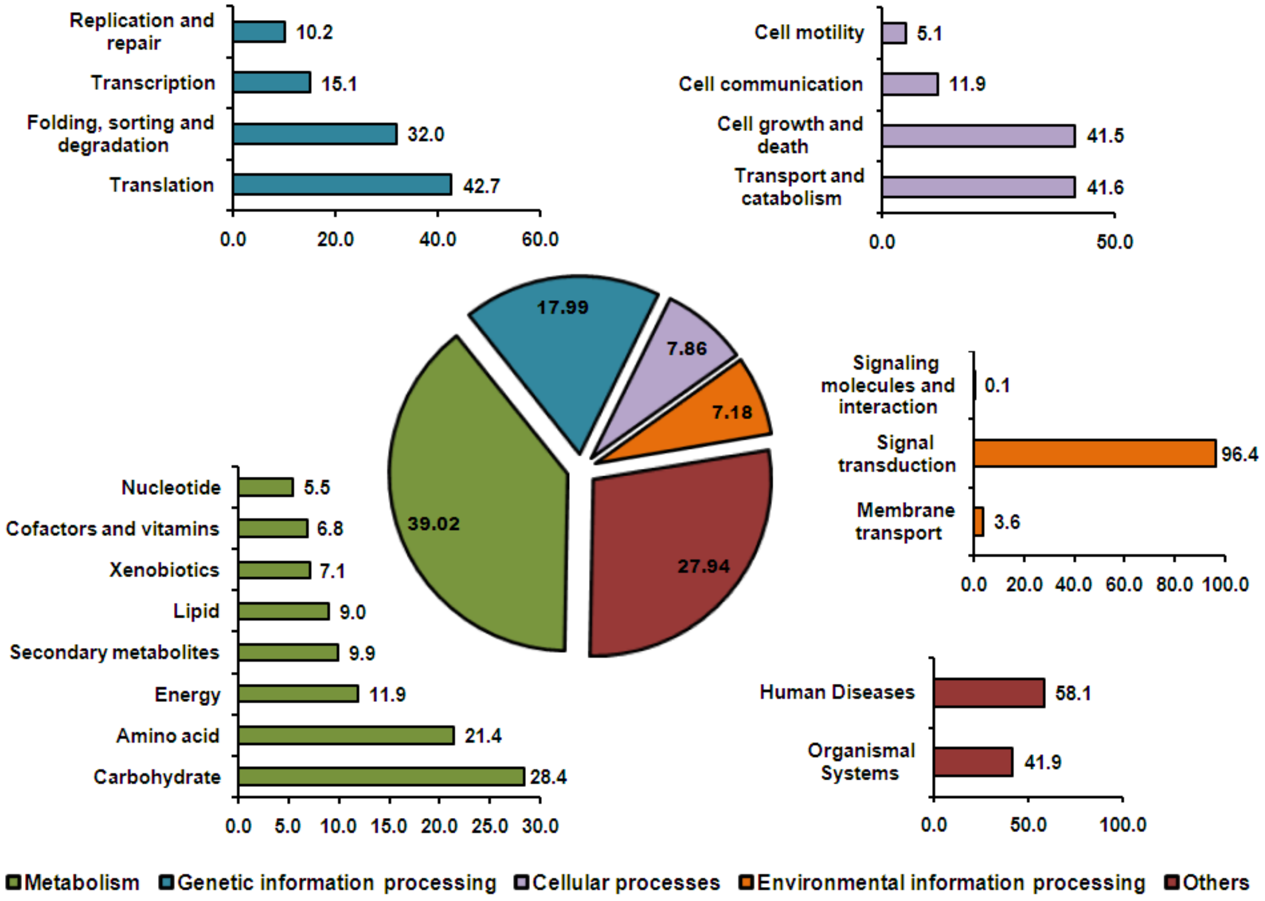

Figure S4. KEGG Orthology (KO) classification and distribution of transcripts identified by KASS into 5 main categories and their sub-categories.

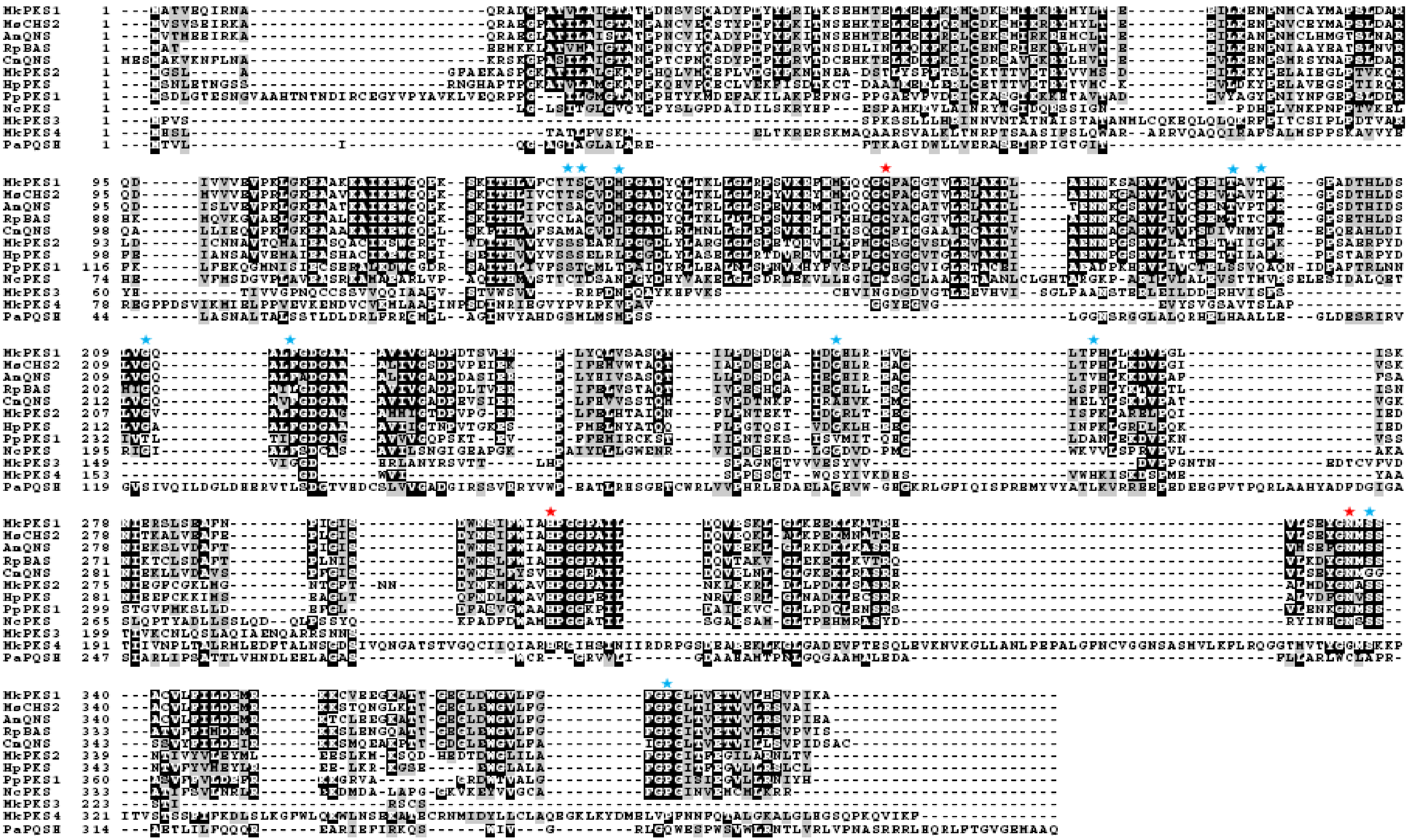

Figure S5. Multiple sequence alignment of full length MkPKSs with characterized PKSIII. AmQNS, *Aegle marmelos* quinolone aynthase (AGE44110); CmQNS, *C. microcarpa* quinolone synthase (BAO05328); HpPKS, *H. perforatum* polyketide synthase (ABP98922); MsCHS2, *Medicago sativa* Chalcone synthase 2 (P30074); NcPKS, *Neurospora crassa* type III Pentaketide Synthase (3EUQ); PaPQSH, *P. aeruginosa* 2-heptyl-3-hydroxy-4(1H)-quinolone synthase (Q9I0Q0); PpPKS1, *Physcomitrella patens* 2'-oxoalkylresorcinol synthase (ABU87504); RpBAS, *R. palmatum* benzalacetone synthase (Q94FV7). The conserved catalytic triad (Cys-164, His-303, and Asn-336) is star marked in red and the active site residues (132, 133, 137, 194, 197, 211, 215, 256, 338, and 375) are star marked in blue (numbering in *M. sativa* CHS)Alignment was built using MAFFT 7.271 and BOXSHADE 3.21.

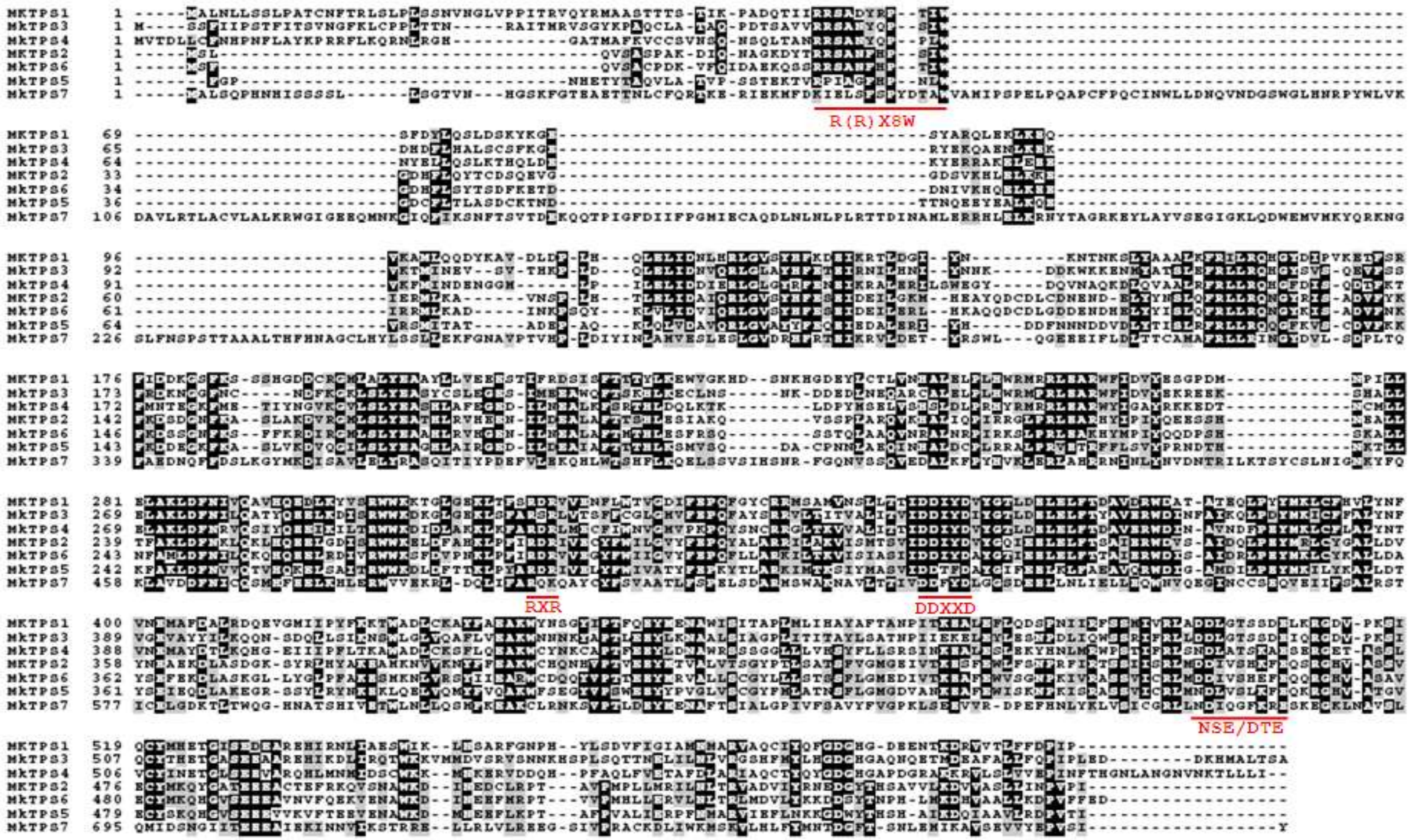

Figure S6. Multiple sequence alignment of full length MkTPSs. The conserved catalytic motifs are marked in red. Alignment was built using MAFFT 7.271 and BOXSHADE 3.21.

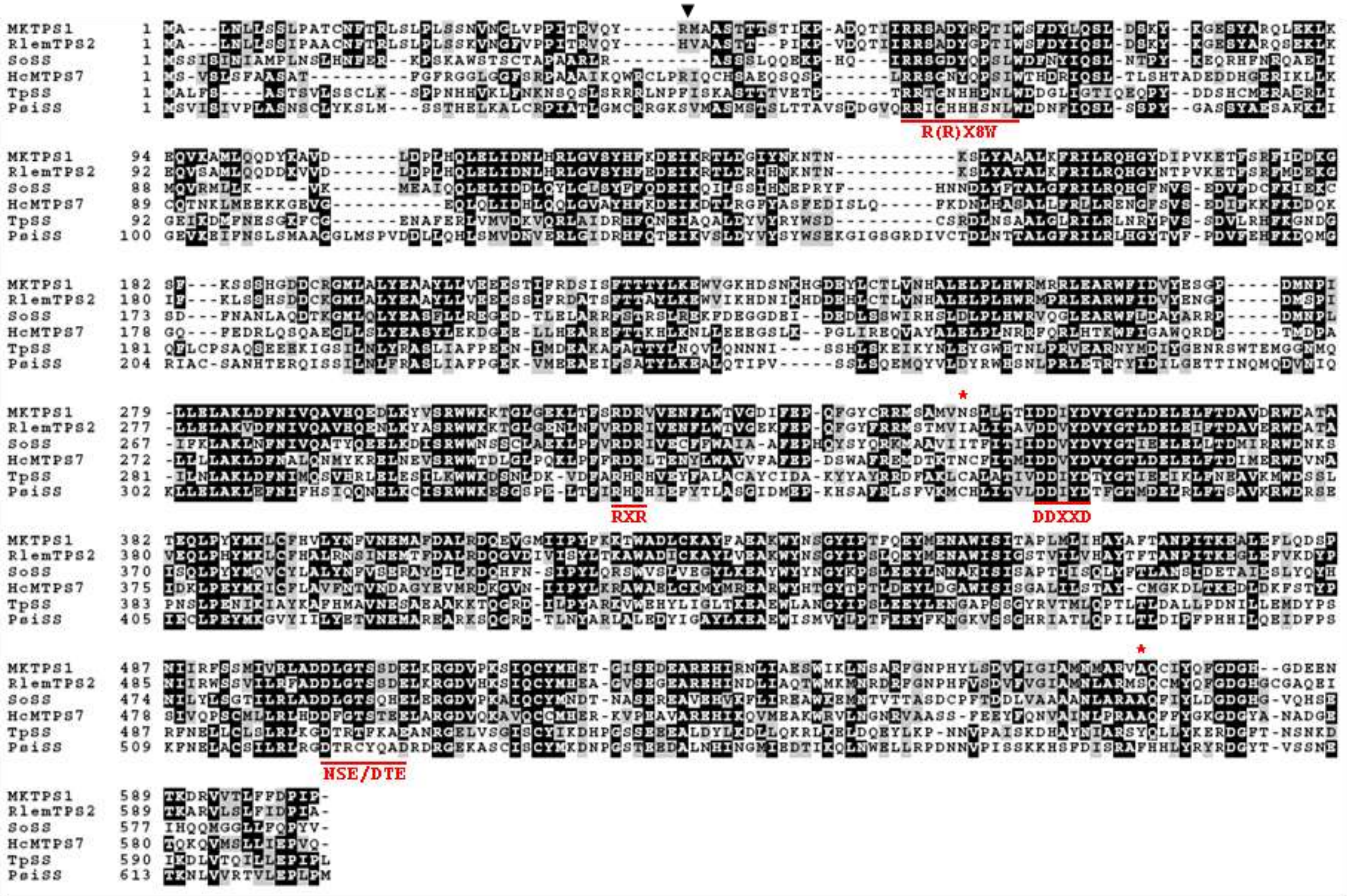
MKTPS1
RlemTPS2
SoSS
HcMTPS7
TpSS
PsiSS
R(R)X8W
RXR
DDXXD
NSE/DTE

Fig. S7. Amino acid alignment of MkTPS1 with other characterized sabinene synthases. Abbreviations with accession numbers: RlemTPS2, *Citrus jambhiri* (Rough lemon) sabinene synthase (BAF73933); HcMTPS7, *Hedychium coronarium* chloroplast monoterpene synthase (AHJ57305); PsSS, *Picea sitchensis* (+)-sabinene synthase (ADU85930);SoSS, *Salvia officinalis* sabinene synthase (O81193); TpSS, *Thuja plicata* sabinene synthase (AGO02736). The substrate binding, Asp-rich domain "DDXXD", Arg-rich "RXR" and "R(R)X8W" motif, as well as a NSE/DTE motif are underlined in red. Cleavage site is marked with arrowhead.

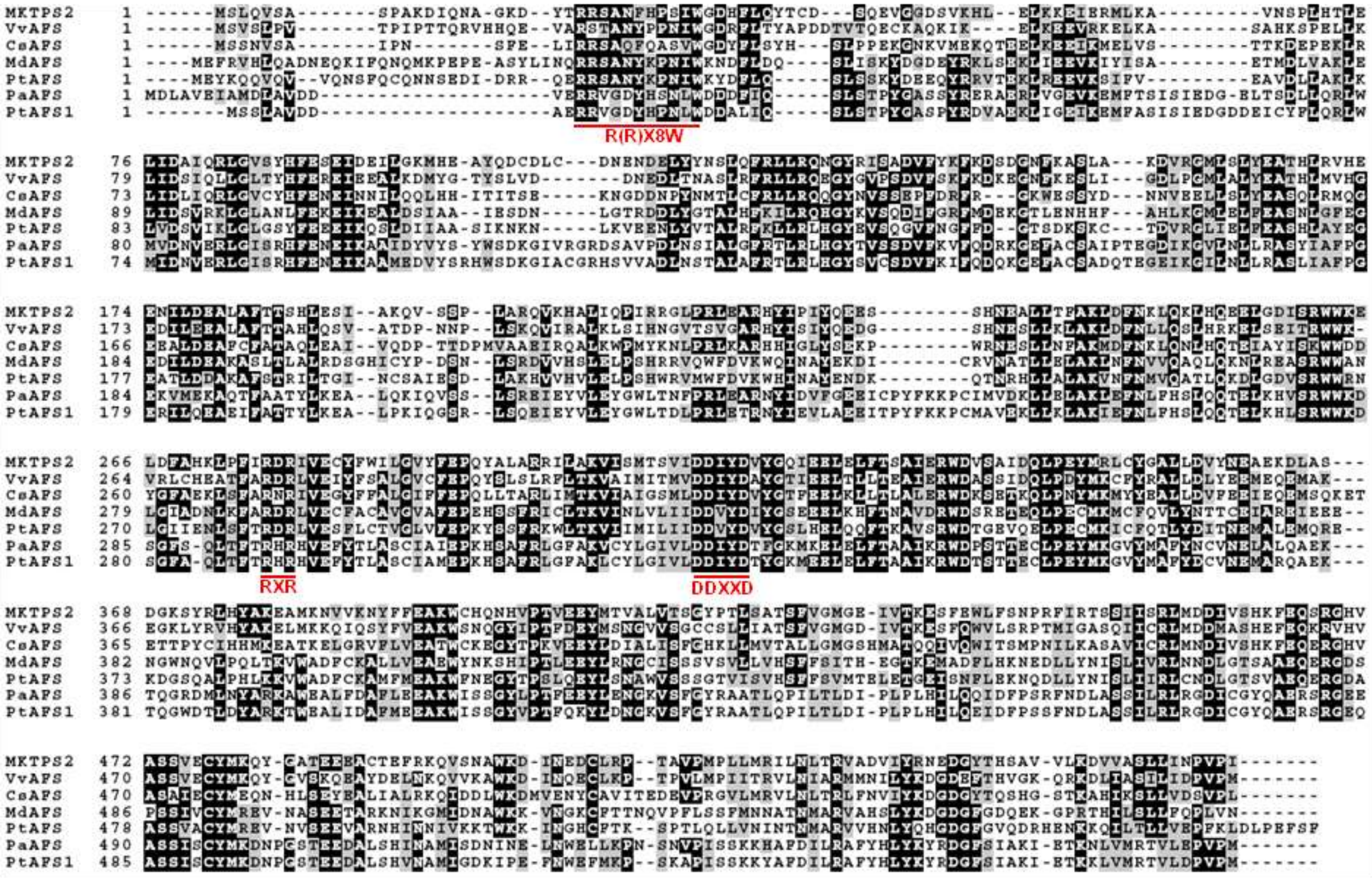
MKTPS2
VvAFS
CsAFS
MdAFS
PtAFS
PaAFS
PtAFS1
R(R)X8W
RXR
DDXXD

Fig. S8. Sequence relatedness of MkTPS2 with other characterized α-farnesene synthases. CsAFS, Cucumis sativus E,E-alpha-farnesene synthase (AAU05951); MdAFS, *Malus domestica (E,E)*-α-farnesene synthase (001280822);PtAFS, *Populus trichocarpa* α-farnesene synthase (AEI52902); PaAFS, *Picea abies (E,E)*-α-farnesene synthase (AAS47697); PtAFS1, *Pinus taeda* α-farnesene synthase (AAO61226)VvAFS1, *Vitis vinifera* α-farnesene synthase (ADR74198); VvAFS2, *Vitis vinifera (E,E)*α-farnesene synthase (ADR7419). Asp-rich domain "DDXXD", Arg-rich "RXR" and "R(R)X8W" motif are underlined in red.

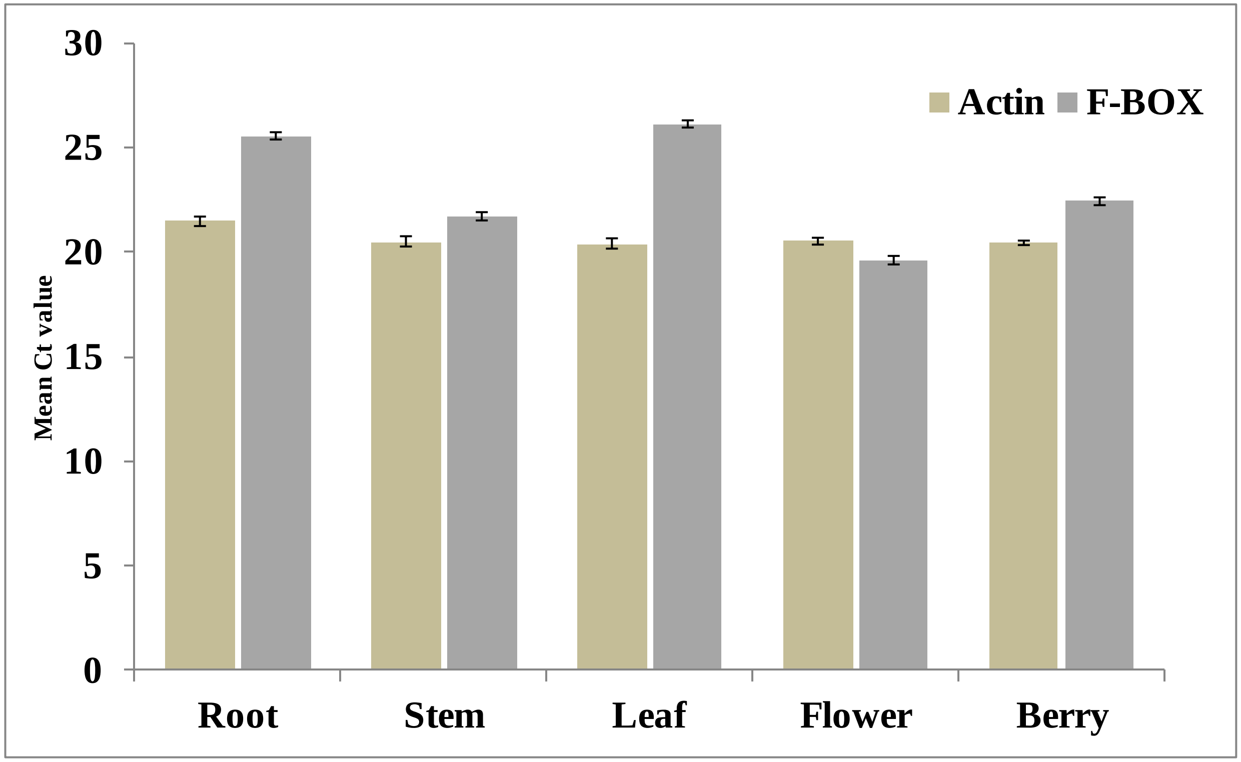

Fig. S9. Expression profiling of reference genes in *M. koenigii* - Mk Actin and Mk F-BOX were used to check the stability in different tissues of M. *koenigii*

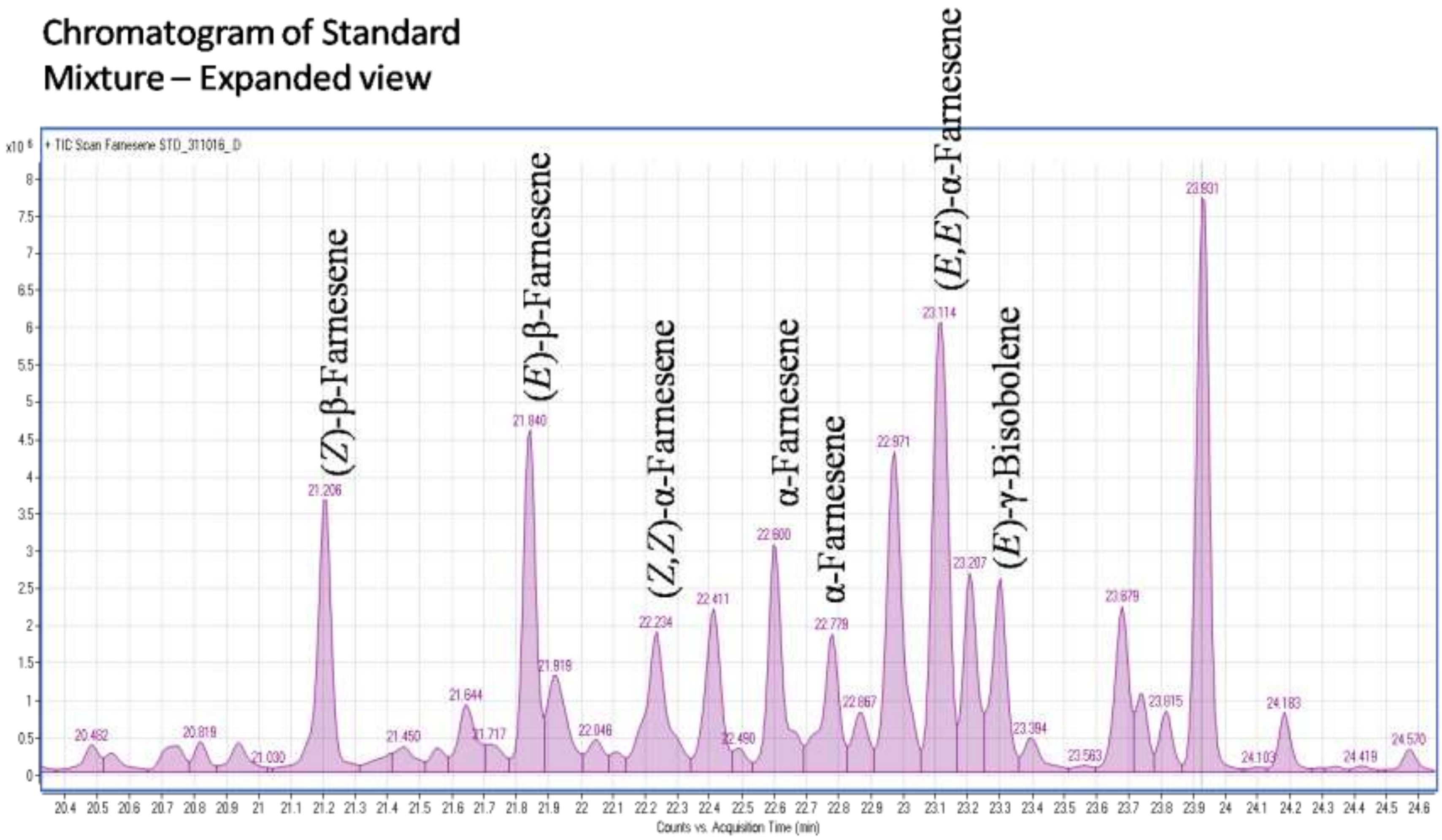

Fig. S10. GC-MS chromatogram of farnesene standard mixture and the respective mass spectra of individual geometric isomers.

(*Z*)-β-Farnesene

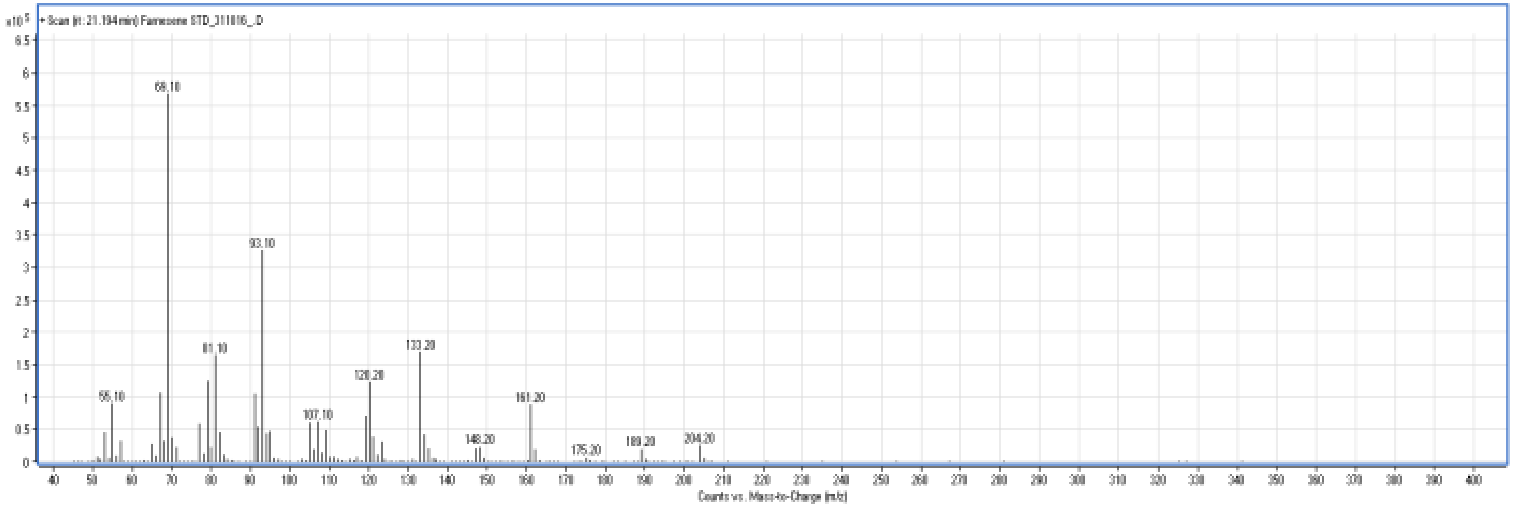

(*E*)-β-Farnesene

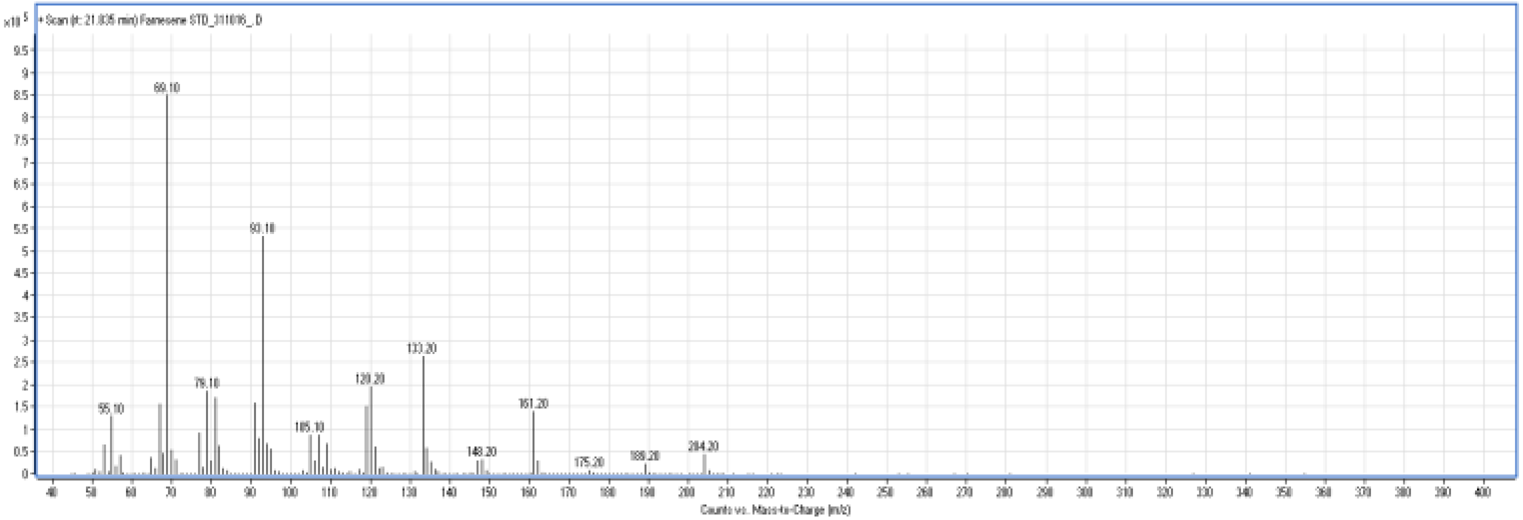

Fig. S10. Cont…

(*Z*,*Z*)-α-Farnesene

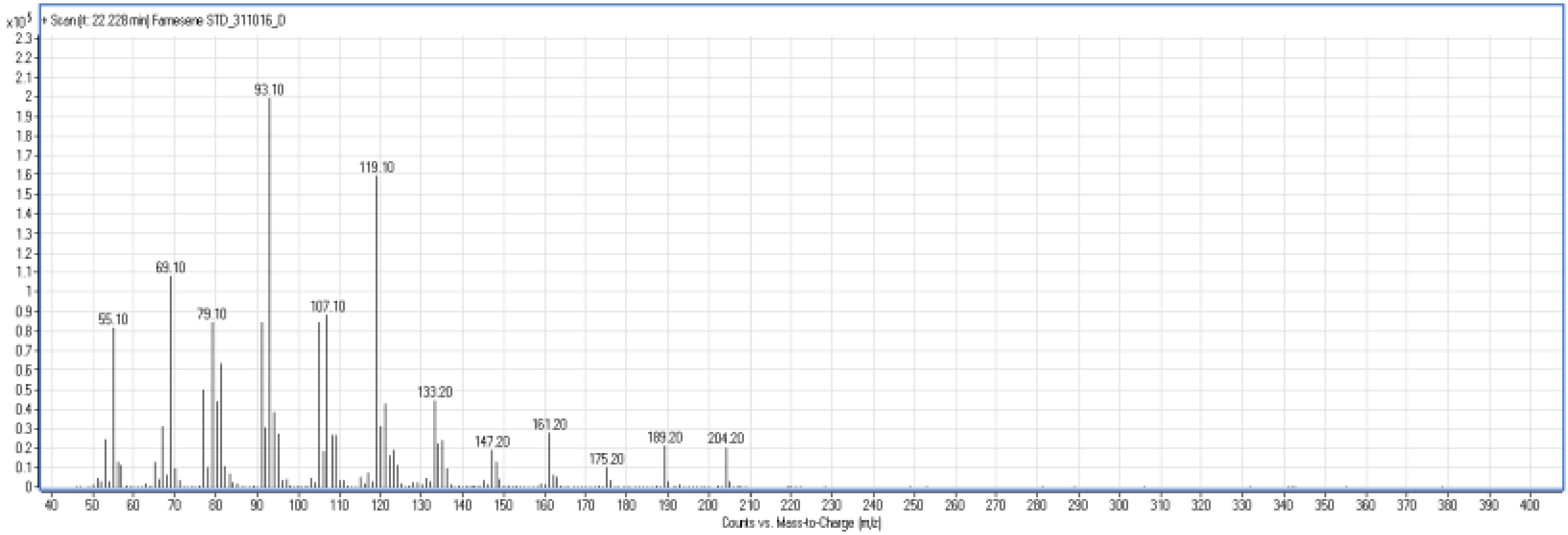

α-Farnesene

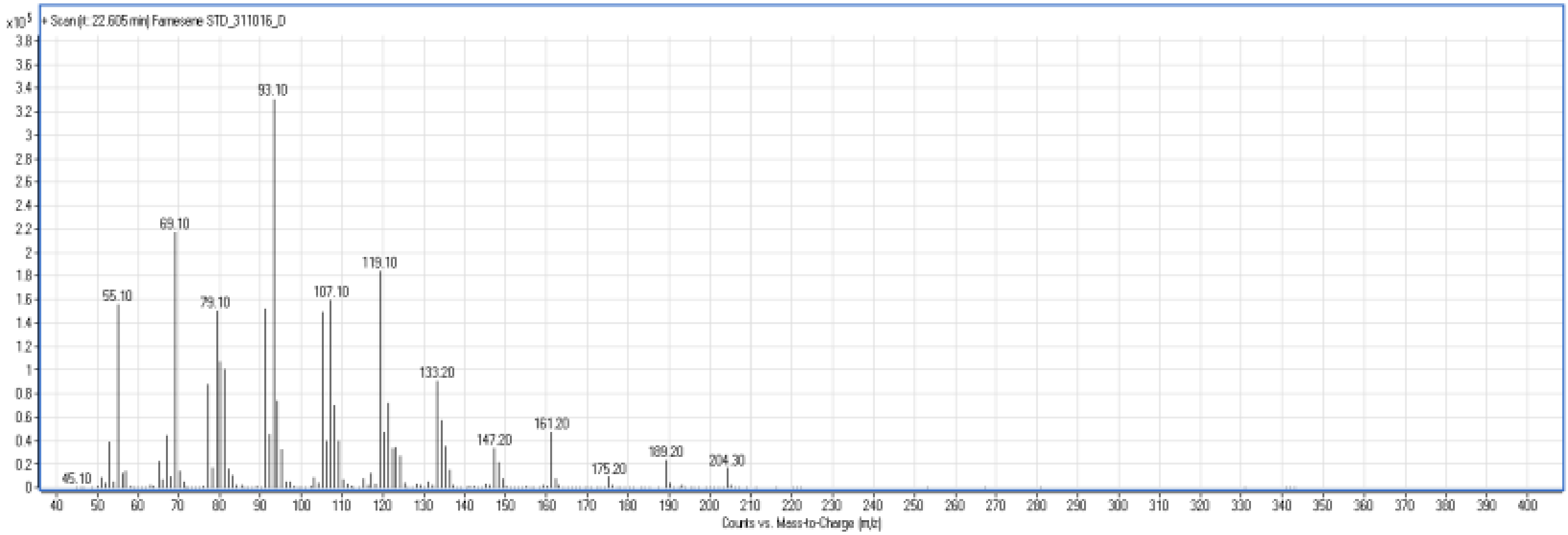

Fig. S10. Cont…

α-Farnesene

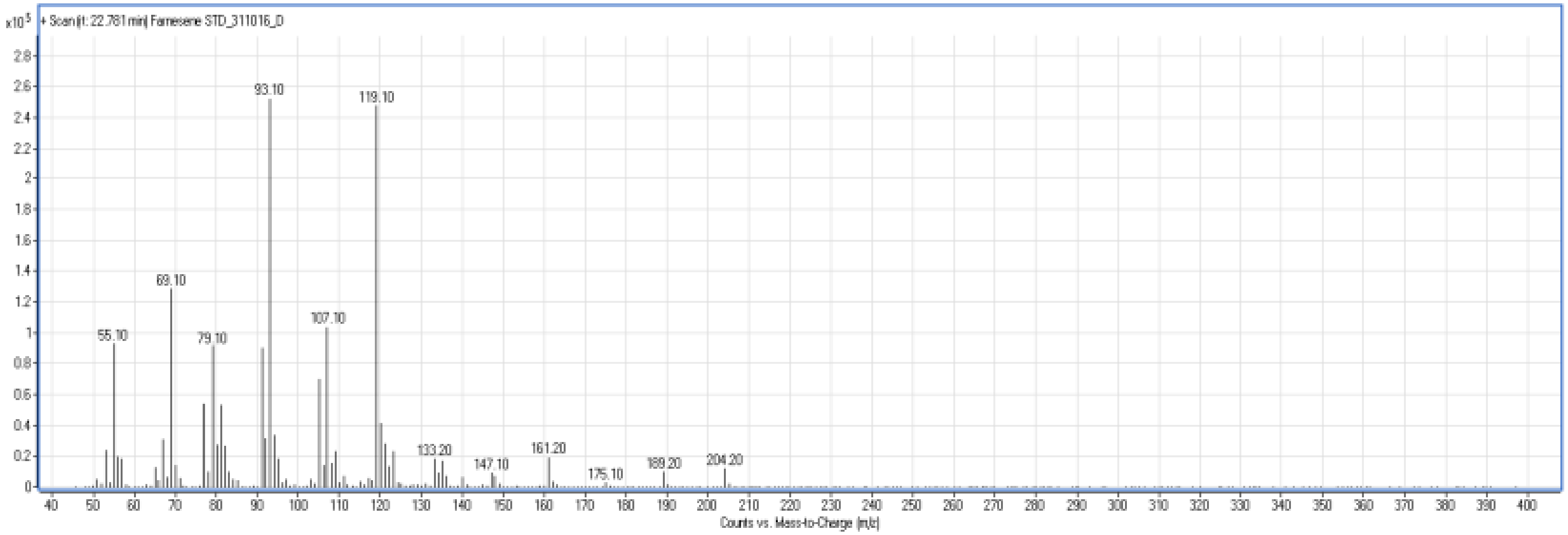

(*E*,*E*)-α-Farnesene

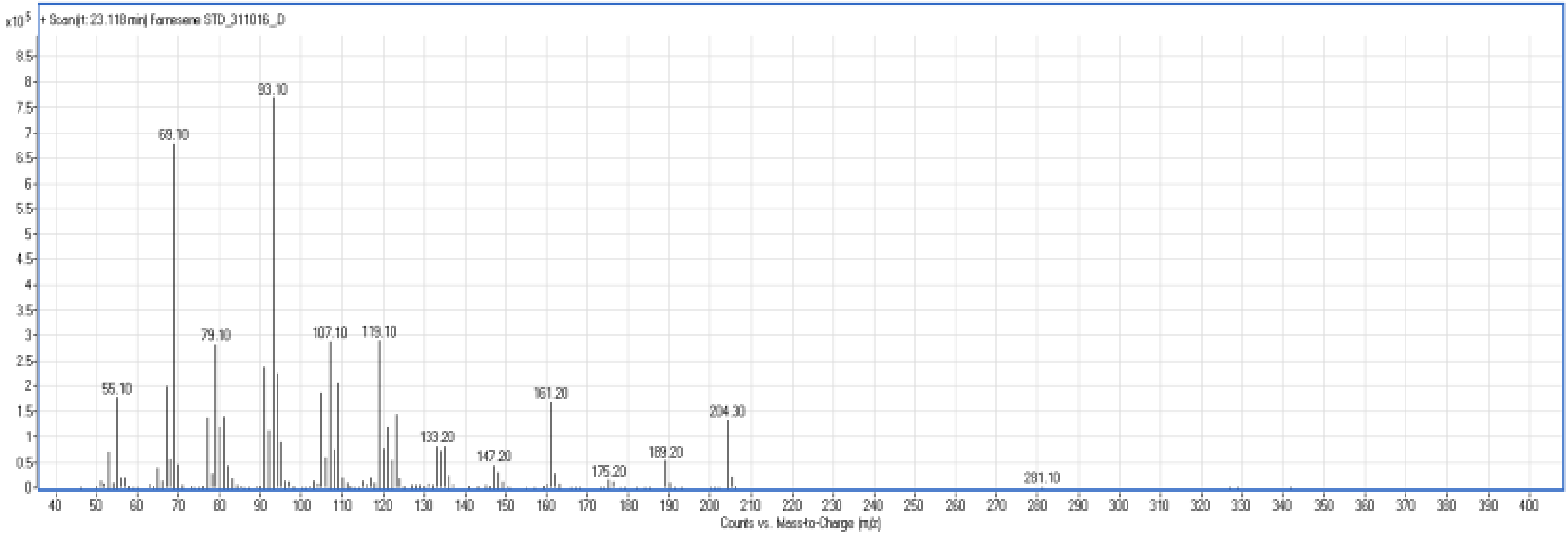

Fig. S10. Cont…

(*E*)-γ-Bisobolene

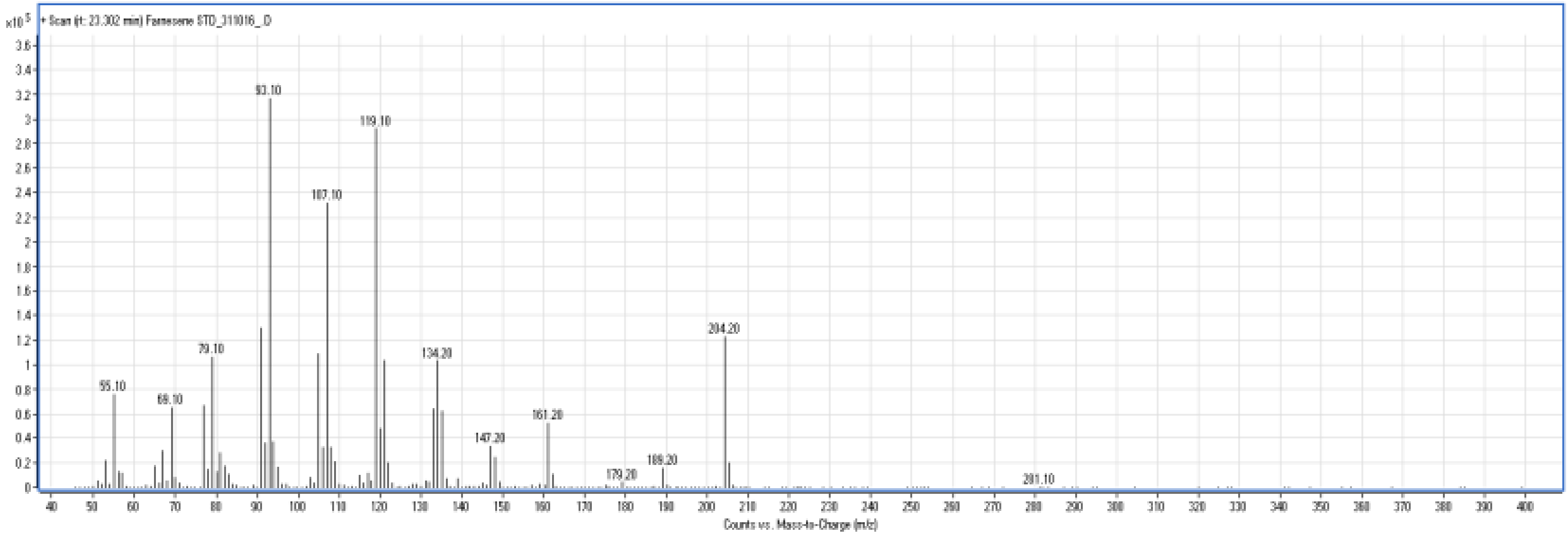

Fig. S10. Cont…

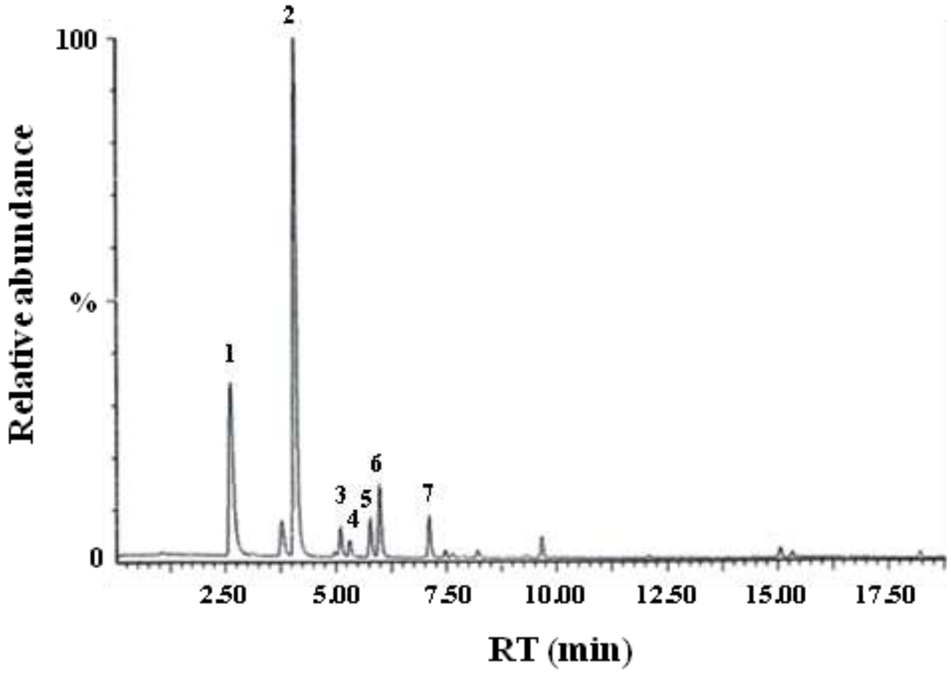

Fig. S11. Headspace volatile analysis of *M. koenigii* leaves by GC-MS. Peaks: 1, α-pinene; 2, sabinene; 3, β-myrcene; 4, α-terpinene; 5, limonene; 6, β-phellandrene; and 7, γ-terpinene.

Accession numbers used in Figure 3

AaOKS1, *Aloe arborescens* octaketide synthase 1 (Q3L7F5); AaPCS, *A. arborescens* pentaketide chromone synthase (Q58VP7); AbDpgA, *Amycolatopsis balhimycina* dihydroxyphenylacetic acid synthase (CAC48378); AhSTS, *Arachis hypogaea* stilbene synthase (BAA78617); AmQNS, *Aegle marmelos* quinolone aynthase (AGE44110); AtCHS, *Arabidopsis thaliana* chalcone synthase (AAA32771); BsNCHS, *Bacillus subtilis* naringenin-chalcone synthase (AAA96613); CsCHS, *Cannabis sativa* chalcone synthase (AAL92879); CmACS, *Citrus microcarpa* acridone synthase (BAO05327); CmQNS, *C. microcarpa* quinolone synthase (BAO05328); CsPKSIIIB, *C. sinensis* polyketide synthase type III B (XP_006487398); ClCHS, *Curcuma longa* chalcone synthase (BAQ35543); ClDCS, *C. longa* diketide CoA synthase (C0SVZ5); ClCURS1, *C. longa* curcumin synthase 1 (C0SVZ6); EcFABH, *Escherichia coli* K-12 beta-ketoacyl-ACP synthase III (P0A6R0); Gh2PS, *Gerbera hybrida* 2-pyrone synthase (P48391); GhCHS1, *G. hybrida* naringenin-chalcone synthase 1 (P48390); GmBPS, *Garcinia mangostana* benzophenone synthase (AEI27291); HaBPS, *Hypericum androsaemum* benzophenone synthase (Q8SAS8); HpOKS, *H. perforatum* octaketide synthase (ACF37207); HpPKS, *H. perforatum* polyketide synthase (ABP98922); HmSTS, *Hydrangea macrophylla* stilbene carboxylate synthase (AAN76182); HsPKS1, *Huperzia serrata* chalcone synthase-like polyketide synthase 1 (ABI94386); MpSTCS, *Marchantia polymorpha* stilbene carboxylate synthase 2 (AAW30010); MsCHS2, *Medicago sativa* Chalcone synthase 2 (P30074); MtPKS1, *Mycobacterium tuberculosis* phenolpthiocerol synthesis type-I polyketide synthase (CEZ67477); NcPKS, *Neurospora crassa* type III Pentaketide Synthase (3EUQ); OsCHS1, *Oryza sativa* Chalcone synthase 1 (A2ZEX7); OsCUS, *O. sativa* Curcuminoid synthase (Q8LIL0); PaPQSD, *Pseudomonas aeruginosa* 2-heptyl-4(1H)-quinolone synthase (P20582); PaPQSH, *P. aeruginosa* 2-heptyl-3-hydroxy-4(1H)-quinolone synthase (Q9I0Q0); PBBS, *Phalaenopsis sp.* bibenzyl synthase (CAA56276); PiPKS, *Plumbago indica* polyketide synthase (BAF44539); PnVPS, *Psilotum nudum* valerophenone synthase (Q9SLX9); PpPKS1, *Physcomitrella patens* 2'-oxoalkylresorcinol synthase (ABU87504); PsCHS, *Pisum sativum* chalcone synthase

(BAA01512); PsCHS, *Pinus strobus* chalcone synthase (CAA06077); PsSBS, *P. strobus* stilbene synthase (CAA87013); RgACS2, *Ruta graveolens* acridone synthase 2 (Q9FSC0); RgCHS1, *R. graveolens* chalcone synthase 1 (Q9FSB9); RpALS, *Rheum palmatum* aloesone synthase (AAS87170); RpBAS, *R. palmatum* benzalacetone synthase (Q94FV7); SaBIS, *Sorbus aucuparia* biphenyl synthase (ABB89212); SbARS1, *Sorghum bicolor* alkylresorcinol synthase 1 (XM_002449699); SbARS2, *S. bicolor* alkylresorcinol synthase 2 (XM_002441794); ScTHNS, *Streptomyces coelicolor* 1,3,6,8-tetrahydroxynaphthalene synthase (Q9FCA7); SgTHNS, *Streptomyces griseus* 1,3,6,8-tetrahydroxynaphthalene synthase (Q54240); VvTHS1, *Vitis vinifera* resveratrol synthase 1 (P28343); ZmCHS2, *Zea mays* chalcone synthase 2 (P24825).

Accession numbers used in Figure 4

AbLIMS, *Abies grandis* (-)-4*S*-limonene synthase (AAB70907); AaLIS, *Actinidia arguta* linalool synthase (ADD81294); AdGDS, *Actinidia deliciosa* germacrene-D synthase (AAX16121); *Antirrhinum majus* nerolidol/linalool synthase 1 (ABR24417); AtTPS02, *Arabidopsis thaliana* Terpene synthase 02 (POCJ43); AtTPS10, *A. thaliana* Terpene synthase10 (Q9ZUH4); AmNES/LIS-1,; CsαPINS, *Cannabis sativa* (+)-α-pinene synthase (ABI21838); CjGES, *Citrus jambhiri* geraniol synthase (BAM29049); RlemTPS2, *C. jambhiri* sabinene synthase (BAF73933); RlemTPS4, C. *jambhiri* δ-elemene synthase (BAP74389); CjβPINS, *C. jambhiri* β- pinene synthase (BAF73933); CjuβFS, *C. junos* (*E*)-β-farnesene synthase (Q94JS8); ClLIMS2, *C. limon* limonene synthase 2 (AAM53946); ClβPINS, *C. limon* (-)-β-pinene synthase (AAM53945); ClγTPNS, *C. limon* γ-terpinene synthase (Q8L5K4); CsiVLS, *C. sinensis* valencene synthase (NP_001275785); CudLIMS, *C. unshiu* limonene synthase (BAD27257); CuLIS, *C. unshiu* linalool synthase (BAP75559); CuβOCS, *C. unshiu* (*E*)-β-ocimene synthase (BAD91046); CuγTPNS, *C. unshiu* γ-terpinene synthase (BAD27259); CbLIS2, *Clarkia breweri* linalool synthase 2 (AAD19840); CcLIS, *C. concinna* linalool synthase (AAD19839); Cmα-FS1, *Cucumis melo* α-farnesene synthase (B2KSJ6); CsαFS, *C. sativus* (E,E)-α-farnesene synthase (AAU05951); CmCPS, *Cucurbita maxima* copalyl diphosphate sythase (AAD04292); GaδCDS, *Gossypium arboreum* (+)-δ-cadinene synthase (AAB41259); HcMTPS7,

Hedychium coronarium chloroplast monoterpene synthase (AHJ57305); MdαFS, *Malus domestica* (*E,E*)-α-farnesene synthase (Q84LB2); MpβFS, *Mentha piperita* β-farnesene synthase (O48935); ObGES, *Ocimum basilicum* geraniol synthase (Q6USK1); OsKSL10, *Oryza sativa Ent*-sandaracopimaradiene synthase (Q2QQJ5); OfTPS4, *Osmanthus fragrans* α-farnesene synthase (AMB57288); Pp*ent*-KS, *Physcomitrella patens* (-)-*ent*-kaurene synthase (BAF61135); PaαBS, *Picea abies E*-α-bisabolene synthase (AAS47689); PaLIS, *P. abies* (-)-linalool synthase (AAS47693); PaαFS, *P. abies E,E*-α-farnesene synthase (AAS47697); PsLIMS, *P. sitchensis* (-)-limonene synthase (ABA86248); PsPINS, *P. sitchensis* pinene synthase (AAP72020); PsSS, *P. sitchensis* (+)-sabinene synthase (ADU85930); *Pinus sylvestris E*-β-farnesene synthase (ADH29869); PtαFS Psβ-FS, *Pinus taeda* α-farnesene synthase (AAO61226); Ptα-FS, *P. taeda* α-farnesene synthase (Q84KL5); PaIPS, *Populus alba* isoprene synthase (Q50L36); PtIPS, *P. tremuloides* isoprene synthase (Q7XAS7); Rcα-FS, *Ricinus communis* α-farnesene synthase (B9RXW0); SoSS, *Salvia officinalis* (+)-sabinene synthase (O81193); SrCPS, *Stevia rebaudiana* copalyl pyrophosphate synthase (AAB87091); TpSS, *Thuja plicata* sabinene synthase (AGO02736); Vvα-FS1, *Vitis vinifera* α-farnesene synthase (ADR74198); VvαFS2, *V. vinifera* α-farnesene/β-ocimene synthase (ADR74207); VvVLS, *V. vinifera* valencene synthase (Q6Q3H2).
